# An Enigmatic Stramenopile Sheds Light on Early Evolution in Ochrophyta Plastid Organellogenesis

**DOI:** 10.1093/molbev/msac065

**Published:** 2022-03-28

**Authors:** Tomonori Azuma, Tomáš Pánek, Alexander K. Tice, Motoki Kayama, Mayumi Kobayashi, Hideaki Miyashita, Toshinobu Suzaki, Akinori Yabuki, Matthew W. Brown, Ryoma Kamikawa

**Affiliations:** 1 Graduate School of Human and Environmental Studies, Kyoto University, Yoshida nihonmatsu cho, Sakyo ku, Kyoto, Japan; 2 Department of Zoology, Faculty of Science, Charles University, Prague, Czech Republic; 3 Department of Biological Sciences, Mississippi State University, Mississippi State, MS, USA; 4 Graduate School of Science, Kobe University, Hyogo, Japan; 5 Japan Agency for Marine-Earth Science and Technology, Yokosuka, Japan; 6 Graduate School of Agriculture, Kyoto University, Kitashirakawa oiwake cho, Sakyo ku, Kyoto, Japan

**Keywords:** Actinophryidae, plastid evolution, aminoacyl-tRNA synthase, gene transfer, phylogenomics, organellar DNA

## Abstract

Ochrophyta is an algal group belonging to the Stramenopiles and comprises diverse lineages of algae which contribute significantly to the oceanic ecosystems as primary producers. However, early evolution of the plastid organelle in Ochrophyta is not fully understood. In this study, we provide a well-supported tree of the Stramenopiles inferred by the large-scale phylogenomic analysis that unveils the eukaryvorous (nonphotosynthetic) protist *Actinophrys sol* (Actinophryidae) is closely related to Ochrophyta. We used genomic and transcriptomic data generated from *A. sol* to detect molecular traits of its plastid and we found no evidence of plastid genome and plastid-mediated biosynthesis, consistent with previous ultrastructural studies that did not identify any plastids in Actinophryidae. Moreover, our phylogenetic analyses of particular biosynthetic pathways provide no evidence of a current and past plastid in *A. sol*. However, we found more than a dozen organellar aminoacyl-tRNA synthases (aaRSs) that are of algal origin. Close relationships between aaRS from *A*. *sol* and their ochrophyte homologs document gene transfer of algal genes that happened before the divergence of Actinophryidae and Ochrophyta lineages. We further showed experimentally that organellar aaRSs of *A. sol* are targeted exclusively to mitochondria, although organellar aaRSs in Ochrophyta are dually targeted to mitochondria and plastids. Together, our findings suggested that the last common ancestor of Actinophryidae and Ochrophyta had not yet completed the establishment of host–plastid partnership as seen in the current Ochrophyta species, but acquired at least certain nuclear-encoded genes for the plastid functions.

## Introduction

Photosynthetic plastids are responsible for the conversion of solar energy to biochemical energy, ATP, and NADPH, both of which are then utilized in biochemical reactions such as carbon fixation and biosynthesis of amino acids, fatty acids, and various prosthetic groups, for example, heme (Plaxton 1996; Kleffmann et al. 2004). The first photosynthetic eukaryote is suggested to have arisen through endosymbiosis between a heterotrophic protistan eukaryote and a cyanobacterium closely related to *Gloeomargarita lithophora* (Ponce-Toledo et al. 2017; Moore et al. 2019). That endosymbiotic event, called primary endosymbiosis, occurred at least 900 Ma in the common ancestor of Archaeplastida comprising land plants, green algae, red algae, glaucophytes, Rhodelphidia, and Picozoa (Gould et al. 2008; Parfrey et al. 2011; Shih and Matzke 2013; Gawryluk et al. 2019; Sibbald and Archibald 2020; Schön et al. 2021; Tice et al. 2021; Irisarri et al. 2022). Subsequently, multiple heterotrophic eukaryotes further acquired green alga-derived or red alga-derived plastids through eukaryote–eukaryote endosymbioses, which have given rise to green alga-derived plastids in chlorarachniophytes, euglenophytes, and green dinoflagellates, and red alga-derived plastids in dinoflagellates, apicomplexans, colpodelids, cryptophytes, haptophytes, and ochrophytes (Adl et al. 2019; Sibbald and Archibald 2020).

Ochrophyta is one of the most diverse groups of photosynthetic eukaryotes (Adl et al. 2019; Sibbald and Archibald 2020), including nonphotosynthetic species that have lost photosynthesis secondarily (Kamikawa et al. 2017; Dorrell et al. 2019; Kayama et al. 2020). The best-known Ochrophyta lineages are the unicellular diatoms (Bacillariophyceae) whose primary production in the ocean contributes almost 20% of the net global primary production (Field et al. 1998; Mann 1999) and the multicellular brown algae (Phaeophyceae) such as giant kelp, which support coastal ecosystems as habitats for aquatic animals (Bringloe et al. 2020). Ochrophyte species possess plastids derived from a red alga, obtained either through secondary endosymbiosis or from a red alga-derived plastid-bearing eukaryote by tertiary endosymbiosis, although the precise origin remains unclear (e.g., Burki et al., 2016; Strassert et al. 2021). Ochrophytes possess plastids bound by four membranes of which the outermost membrane is fused with the endoplasmic reticulum (ER) and the nuclear membrane (Andersen 2004). Despite the ecological and evolutionary importance of Ochrophyta, early plastid evolution in the group is not fully understood. A recently popularized hypothesis suggests that the plastid was acquired after the divergence of Ochrophyta from closely related lineages (Stiller et al. 2014; Burki et al., 2016; Dorrell et al. 2017; Strassert et al. 2021).

Ochrophyta is part of the Stramenopiles, a large eukaryotic assemblage that unites them together with Pseudofungi such as the pathogenic oomycetes (Derevnina et al. 2016), Sagenista, and Opalozoa (e.g., *Blastocystis* spp. that inhabit human intestines [Cavalier-Smith and Chao 2006; Tan 2008; Gentekaki et al. 2017]). In many previously reported phylogenetic trees, Ochrophyta is reconstructed as sister to Pseudofungi (e.g., Cavalier-Smith and Chao 2006; Derelle et al. 2016; Cenci et al. 2018). However, it remains unclear whether Pseudofungi is actually the extant closest relative to Ochrophyta, as phylogenetic positions of some lineages of the Stramenopiles have not yet been confirmed by genome- or transcriptome-based phylogenomic analyses. The addition of more Stramenopiles taxa to phylogenomic analyses would provide deeper insight into the closest living relative of Ochrophyta and its in-depth analyses would help us understand the early plastid evolution.

Actinophryidae includes enigmatic eukaryvorous, heterotrophic protists belonging to the Stramenopiles (Adl et al. 2019). Their spherical cells lack cilia, but possess a number of microtubule-supported, radiating axopodia (Ockleford and Tucker 1973; Suzaki et al. 1980; Sakaguchi et al. 1998; Mikrjukov and Patterson 2001; Cavalier-Smith and Scoble 2013). Ultrastructural studies have not detected any plastid or plastid-like structures. The phylogenetic position of Actinophryidae remains unclear, despite its detailed morphological characterization and molecular phylogenetic analyses based on genes for 18S rRNA and actin have been conducted (Ockleford and Tucker 1973; Suzaki et al. 1980; Sakaguchi et al. 1998; Mikrjukov and Patterson 2001; Nikolaev et al 2004; Cavalier-Smith and Scoble 2013).

In this study, we generated transcriptome data of *Actinophrys sol*, the type species of the genus *Actinophrys*, which unveiled previously unknown aspects of its evolution and cell biology. Our phylogenomic analysis employing a 239 protein-dataset comprising 75 taxa and 75,984 sites provides a well-resolved tree of the Stramenopiles and reveals *A. sol* is closely related to Ochrophyta, giving rise to the phylogenetic clade comprising Ochrophyta and Actinophryidae. Genome sequencing and transcriptome-based metabolic reconstruction provide no evidence of a current plastid, consistent with the previous transmission electron microscopic observation. Based on our finding that the nonphotosynthetic deep-branching *A. sol* possesses mitochondrial aminoacyl-tRNA synthase genes that share evolutionary origins with plastid-localized counterparts of photosynthetic Ochrophyta, we discuss the organellogenesis for the plastid acquisition of Ochrophyta.

## Results and Discussion

### The Close Relationship Between Actinophryidae and Ochrophyta

To obtain hundreds of conserved protein sequences of *A. sol* for phylogenomic analysis, we performed Illumina-based RNA sequencing of *A. sol* NIES-2497 cocultivated with the prey green alga *Chlorogonium capillatum* NIES-3374. From the total assembled contigs, those derived from the prey alga were removed (Supplementary fig. S1, Supplementary Material online). As a result, we obtained 39,797 contigs highly likely derived from *A. sol*. The quality of the transcriptome data was evaluated by BUSCO v2 (Simão et al. 2015) and we detected 279 of 303 “core eukaryotic genes.”

**Fig. 1.**
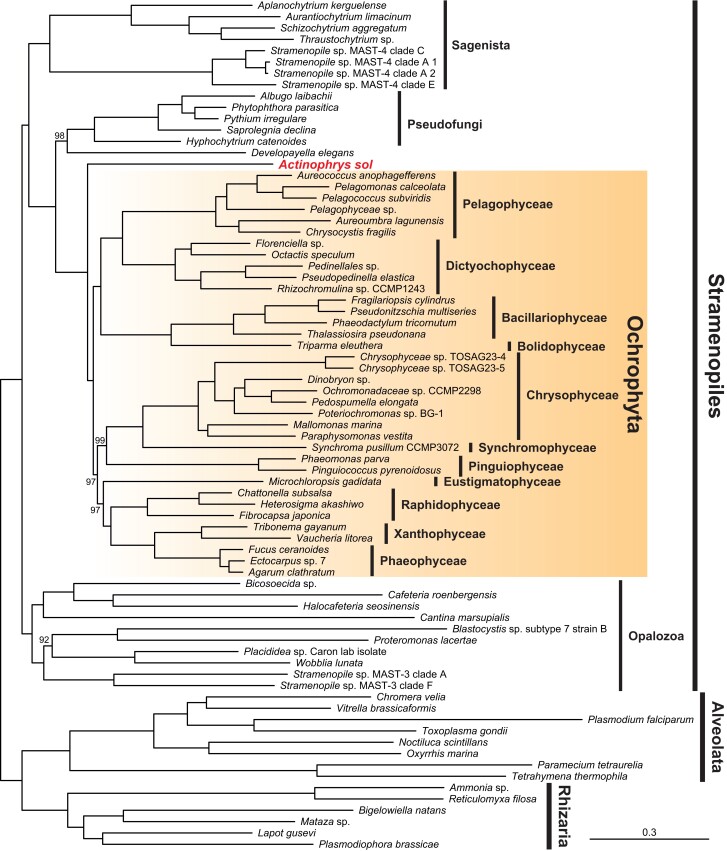
Phylogenomic tree of the Stramenopiles. Tree reconstruction was conducted by ML analysis with a dataset comprised of 75 taxa and 75,984 amino acid sites under LG + G4 + F + C60-PMSF model. Species of Alveolata and Rhizaria are regarded as outgroup taxa. The numbers on branches are the ML bootstrap values. If not shown, the nodes are fully supported.

We constructed a phylogenomic dataset containing *A. sol* and sampled Ochrophyta taxa more comprehensively than or comparable to previous studies (e.g., Derelle et al. 2016; Noguchi et al. 2016; Thakur et al. 2019; Di Franco et al. 2022). With the dataset comprised of 75 taxa and 75,984 amino acid sites, the maximum likelihood (ML) analysis under the site heterogenous LG + G4 + F + C60-PMSF (Wang et al. 2018) model provided a robust tree of the Stramenopiles. The phylogenomic analysis reconstructed four well-known clades of the Stramenopiles, comprising Pseudofungi, Sagenista, Opalozoa, and Ochrophyta (fig. 1). All the nodes in the Ochrophyta clade were supported by high bootstrap values (97–100%).

Ochrophyta has been taxonomically divided into two large groups—Diatomista and Chrysista: Bolidophyceae, Bacillariophyceae, Dictyochophyceae, Pinguiophyceae, and Pelagophyceae are subassemblages of the former, and Chrysophyceae, Synchromophyceae, Phaeophyceae, Xanthophyceae, Raphidophyceae, and Eustigmatophyceae of the latter (Adl et al. 2019). The current tree supports the above two large groups with exception of the position of Pinguiophyceae (Adl et al. 2019), which branches within Chrysista with 97% bootstrap support (fig. 1). Chrysista is further divided into two or more subgroups in our analysis. Together with Pinguiophyceae, Chrysophyceae and Synchromophyceae were reconstructed as monophyletic with 99% ML bootstrap support, and the other lineages, that is, Eustigmatophyceae, Raphidophyceae, Xanthophyceae, and Phaeophyceae, were also monophyletic with 97% bootstrap support (fig. 1). Note that some lineages such as Phaeothamniophyceae are still not included in this analysis and that phylogenetic positions of some of the lineages in this tree, such as Eustigmatophyceae (fig. 1), are inconsistent with previously reported phylogenomic studies; Eustigmatophyceae was reconstructed as more closely related to Chrysophyceae and Synchromophyceae (Ševčíková et al. 2015; Di Franco et al. 2022). Although the position of Pinguiophyceae close to Chrysophyceae and Synchromophyceae (fig. 1) is consistent with those of Dorrell et al. (2021) and Strassert et al. (2021), this relationship is not supported by the analysis in Di Franco et al. (2022).

The tree also resolves deeper relationships in the Stramenopiles (fig. 1). Opalozoa is the deepest branching lineage, and Sagenista is the second deepest in the current tree of the Stramenopiles (fig. 1), consistent with a previous study (Noguchi et al. 2016). Similarly, Pseudofungi is more closely related to Ochrophyta (fig. 1), as seen in previous studies (Cavalier-Smith and Chao 2006; Riisberg et al. 2009). Most importantly, *A. sol* was not nested in the above well-known clades of the Stramenopiles but branched sister to the clade of the photosynthetic lineages, Ochrophyta, with the full bootstrap support (fig. 1). Even after stepwise removal of fast-evolving sites and heterotachious sites, the topology of the tree (fig. 1) including the sister relationship between *A. sol* and Ochrophyta was supported by high ultrafast bootstrap values (Supplementary fig. S2, Supplementary Material online). Our coalescence-based phylogenomic analysis using ASTRAL-III (Zhang et al. 2018) also recovered the close relationship between *A*. *sol* and Ochrophyta with full support (100/1.0 astral bootstrap/local posterior probability), although the sister relationship of the two lineages is not well resolved (0.53/0.80) (Supplementary fig. S3, Supplementary Material online).

Although the sister relationship of Actinophryidae and Ochrophyta might require more systematic evaluation, the phagotrophic eukaryvorous lineage Actinophryidae is key to understanding the origin and evolution of Ochrophyta.

### No Evidence for Plastid DNA in *A. sol*

As *A. sol* is a eukaryvorous nonphotosynthetic protist and the closest relative of Ochrophyta, it might provide insight into the early evolution of the ochrophyte plastid. To investigate whether *A. sol* possesses a nonphotosynthetic plastid sharing the same origin with the ochrophyte plastid, we first performed DNA sequencing of *A. sol* cultivated with the prey green alga *C. capillatum*. We detected 58 contigs (247,952 bp in length in total) that showed nucleotide sequence similarity to either of partial chloroplast genome sequences of *C. capillatum* (GenBank accession number: KT625085–KT625091) and one contig (22,647 bp in length) with nucleotide sequence similarity to a partial mitochondrial genome of the green alga *C. elongatum* (GenBank accession number: Y13644). We additionally detected a 53,041 bp-long circularly mapping sequence, of which encoded protein sequences were homologous to those of mitochondrial genomes of the Stramenopiles such as the oomycete *Aphanomyces invadans* (GenBank accession number: KX405005), indicating that the contig is of *A. sol* mitochondria (deposited in DNA Data Bank of Japan under the accession number LC650202). The coverage of the *A. sol* mitochondrial DNA was ca. 11 (Supplementary table S1, Supplementary Material online). Then, we compared read coverages of mitochondrial DNAs and plastid DNAs of several nonphotosynthetic plastid-bearing ochrophytes (see Supplementary table S1, Supplementary Material online). Since coverages of both genomes were comparable to one another in each species, plastid DNA would be detected in *A. sol* if present. As there are no other contigs with sequence similarity to organellar DNAs, it is highly likely that *A. sol* lacks a plastid genome.

To support this possibility, we further searched plastid-targeted protein homologs responsible for organellar DNA replication, transcription, and translation from the *A. sol* transcriptome data (Supplementary dataset S1, Supplementary Material online). We detected transcripts for plant and protist organellar DNA polymerase, translation initiation factor, translation elongation factor, ribonuclease HII, organellar single-subunit RNA polymerase, 50S and 30S ribosomal proteins, ribosome recycling factor, peptide chain release factor, and aminoacyl-tRNA synthetases (aaRSs) of *A. sol*. However, these transcripts are rather homologous to mitochondrial-targeted proteins of other eukaryotes (Supplementary dataset S1, Supplementary Material online). Mitochondrial-targeting sequences were predicted in 42 from 66 transcripts for organellar DNA replication, transcription, and translation, whereas 24 of them are truncated or possess N-terminal extensions containing no detectable targeting signals (Supplementary dataset S1, Supplementary Material online). None of the detected sequences confidently possesses the N-terminal extensions including a signal peptide followed by a transit peptide-like region with phenylalanine, tyrosine, tryptophan, or leucine at the first position, which are typical plastid-targeting sequences of complex plastids including the ochrophyte plastid (Maier et al. 2015) (Supplementary dataset S1, Supplementary Material online).

### No Transcriptomic Evidence for Plastid-Type Biosynthetic Pathways in *A. sol*

Plastids in photosynthetic and nonphotosynthetic Ochrophyta play crucial roles for various metabolisms such as glycolysis/gluconeogenesis, biosynthesis of fatty acids, lipids, amino acids, heme, riboflavin, isoprenoids, Fe–S clusters, and the pentose phosphate pathway (Plaxton 1996; Kleffmann et al. 2004; Kamikawa et al. 2017; Dorrell et al. 2019; Kayama et al. 2020). Even in secondary heterotrophic ochrophytes that bear nonphotosynthetic plastids, transcriptomic analyses detect large numbers of transcripts for plastid functions and biogenesis (Kamikawa et al. 2017; Dorrell et al. 2019; Kayama et al. 2020). If *A. sol* possesses a metabolically active nonphotosynthetic plastid sharing the origin with plastids of Ochrophyta, at least some transcripts encoding proteins involved in the plastid biosynthesis should be detected. We surveyed homologs of 114 proteins that were involved in the above metabolisms by homology-based search (*E*-value < *e*^−5^) with photosynthetic diatom and land plant sequences as queries. The procedure detected 60 transcripts of which encoded protein sequences were homologous to the 38 query proteins. However, they were rather homologous to cytosolic or mitochondrial proteins of other eukaryotes (Supplementary dataset S2, Supplementary Material online). Indeed, there were no confidently recognizable plastid-targeting sequences at the N-termini of the detected homologs (Supplementary dataset S2, Supplementary Material online). Rather, some have N-terminal mitochondrial transit peptides, others possess only signal peptides or lack any N-terminal extension (Supplementary datasets S2, Supplementary Material online). In addition, we also surveyed homologs of transporters known to be localized in the plastid membranes. In the *A. sol* transcriptome data, there are no plastid-targeted homologs of translocators localized in the four membranes of the Ochrophyta plastid: plastid triose phosphate transporters (Moog et al. 2015, 2020), the symbiont-specific ER-associated degradation-like machinery, and translocons at the innermost and the second innermost envelope membrane of chloroplast (TOC/TIC) (Moog et al. 2011; Stork et al. 2013; Maier et al. 2015) (Supplementary dataset S3, Supplementary Material online). Any plastids would utilize at least some components of TIC/TOC to import nuclear-encoded plastid proteins across membranes. Although we detected the Sec61 complex protein sequences facilitating protein import across the outermost membrane of the Ochrophyta plastids, the detected transcripts do not necessarily indicate the presence of a plastid as the Sec61 complex also localizes in the ER membrane of plastid-lacking eukaryotes (Osborne et al. 2005).

An alternative survey employing the presence or absence of N-terminal plastid-targeting sequences, using ASAFind (Gruber et al. 2015), was also applied to the *A. sol* transcriptome data (see Materials and Methods). Although 48 *A. sol* deduced proteins with clear homology to proteins found in other eukaryotes were predicted to possess candidate N-terminal plastid-targeting sequences, none of the functions assigned to these proteins is known to be exclusively functional in any plastids (Supplementary dataset S4, Supplementary Material online). Thus, we did not consider the detected candidates as evidence of a plastid in *A. sol*.

**Fig. 2.**
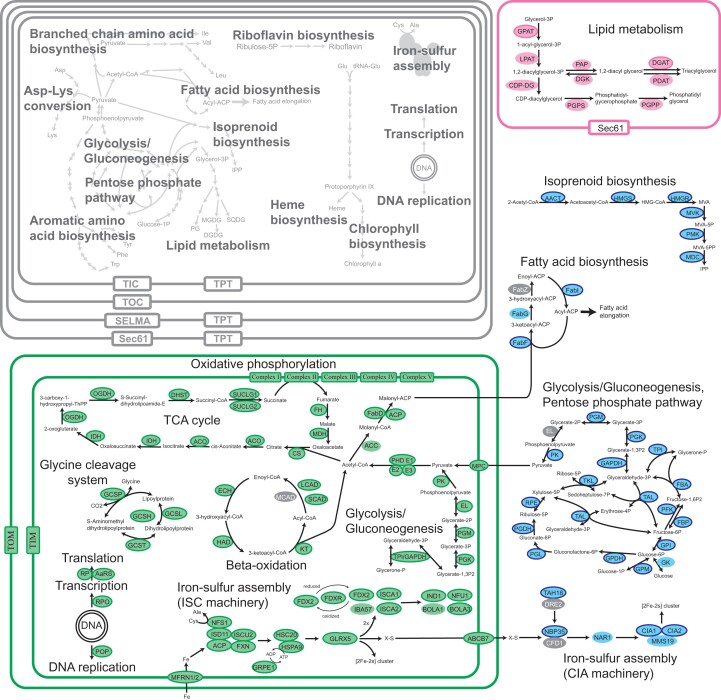
Representative metabolisms deduced from the transcriptome data of *Actinophrys sol*. Whereas gray lines and circles show undetected biochemical reactions and involved proteins, respectively, colored lines show those deduced from the transcriptome data. Light green circles enclosed by green lines show proteins with detectable mitochondria-targeting sequences. Light pink circles enclosed by pink lines show possible ER proteins with detectable signal peptides but lacking transit peptide-like regions and the ASAFAP motif. Light blue circles enclosed by purple lines show cytosolic proteins. Circles lacking enclosing lines show proteins that are N-terminally truncated. AACT, acetoacetyl-CoA thiolase; AaRS, aminoacyl-tRNA synthase; ACC, acetyl-CoA carboxylase; ACO, aconitate hydratase; ACP, acyl carrier protein; BOLA1, ISC targeting factor; BOLA3, ISC targeting factor; CDP-DAGS, CDP-diacylglycerol synthase; CFD1, iron–sulfur cluster assembly protein Cfd1CIA1, CIA targeting complex Cia1; CIA2, CIA targeting complex Cia2; CS, citrate synthase; DGAT, diacylglycerol acyltransferase; DGK, diacylglycerol kinase; DHST, dihydrolipoamide succinyltransferase; DRE2, electron carrier Dre2; ECH, enoyl-CoA hydratase; EL, enolase; FabD, malonyl-CoA:ACP transacylase; FabF, 3-oxoacyl-ACP synthase II; FabG, 3-oxoacyl-ACP reductase; FabI, enoyl-ACP reductase; FabZ, 3-hydroxyacyl-ACP dehydratase; FBA, fructose 1,6-bisphosphate aldolase Class I; FBP, fructose-1,6-bisphosphatase; FDX2, ferredoxin; FDXR, ferredoxin reductase; FH, fumarate hydratase, Class I; FXN, frataxin; GAPDH, glyceraldehyde 3-phosphate dehydrogenase; GCSH, glycine cleavage system H protein; GCSL, glycine cleavage system L protein; GCSP, glycine cleavage system P protein; GCST, glycine cleavage system T protein; GK, glucokinase; GLRX5, monothiol glutaredoxin; GPAT, glycerol-3-phosphate O-acyltransferase; GPDH, glucose-6-phosphate 1-dehydrogenase; GPI, glucose-6-phosphate isomerase; GPM, phosphoglucomutase; GRPE1, nucleotide exchange factor; HAD, 3-hydroxyacyl-CoA dehydrogenase; HMGR, hydroxymethylglutaryl-CoA reductase; HMGS, hydroxymethylglutaryl-CoA synthase; HSC20, J-type cochaperone; HSPA9, Hsp70 chaperone; IBA57, ISC protein; IDH, isocitrate dehydrogenase; IND1, [4Fe–4S] cluster-binding protein; ISCA1, ISC protein; ISCA2, ISC protein; ISCU2, scaffold protein; ISD11, cysteine desulfurase; KT, 3-ketoacyl-CoA thiolase; LCAD, long-chain-acyl-CoA dehydrogenase; LPAT, lysophosphatidate acyltransferase; MCAD, medium-chain-acyl-CoA dehydrogenase; MDC, diphosphomevalonate decarboxylase; MDH, malate dehydrogenase; MMS19, CIA targeting complex Mms19; MVK, mevalonate kinase; NAR1, iron–sulfur cluster assembly protein Nar1NBP35, CIA scaffold protein Nbp35; NFS1, cysteine desulfurase; NFU1, [4Fe–4S] cluster-binding protein; OGDH, 2-oxoglutarate dehydrogenase E1 component; PAP, phosphatidic acid phosphatase; PDAT, phospholipid:diacylglycerol acyltransferase; PDH E1a, pyruvate dehydrogenase E1 subunit alpha protein; PDH E1b, pyruvate dehydrogenase E1 subunit beta protein; PDH E2, pyruvate dehydrogenase E2 subunit; PDH E3, pyruvate dehydrogenase E3 subunit; PFK, phosphofructokinase; PGDH, 6-phosphogluconate dehydrogenase; PGK, phosphoglycerate kinase; PGL, 6-phosphogluconolactonase; PGM, phosphoglycerate mutase; PGPP, phosphatidylglycerophosphatase; PGPS, phosphatidylglycerophosphate synthase; PK, pyruvate kinase; PMK, phosphomevalonate kinase; POP, plant and protist organellar DNA polymerase; RP, ribosomal protein; RPE, ribulose-phosphate 3-epimerase; RPO, RNA polymerase; SCAD, short-chain-acyl-CoA dehydrogenase; Sec61, Sec61 complex; SELMA, symbiont-specific ER-associated degradation (ERAD)-like machinery; SDH, succinate dehydrogenase; SUCLG1, succinyl-CoA synthetase alpha subunit; SUCLG2, succinyl-CoA synthetase beta subunit; TAH18, diflavin reductase Tah18; TAL, transaldolase; TIC/TOC, translocon at the inner/outer envelope membrane of chloroplasts; TKL, transketolase; TPI, triose phosphate isomerase; TPT, triose phosphate transporter.

Lack of the molecular evidence for retained plastids in *A. sol* due to insufficient transcriptome quality is unlikely as we have detected many mitochondrial-targeted protein homologs that are responsible for representative mitochondrial metabolisms. By homology-based survey, 132 sequences were detected as homologs to 97 of the 156 query proteins for mitochondrial metabolism and translocons. Of the 132 sequences, 15 sequences were of those encoded in the *A. sol* mitochondrial genome, and 83 sequences were predicted to possess mitochondrial-targeting sequences at the N-termini (Supplementary dataset S5, Supplementary Material online). The detected mitochondrial sequences could reconstruct the major mitochondrial metabolic pathway of *A. sol* (fig. 2). Representative metabolisms in the cytosolic and ER compartments were also reconstructed by the transcriptome data of *A. sol* (fig. 2; Supplementary datasets S2–S3, S5, Supplementary Material online). These mitochondrial, cytosolic, and/or ER metabolisms included the mevalonate (MVA) pathway of isoprenoid biosynthesis, FASII fatty acid biosynthesis, and lipid biosynthesis. However, no pathway was identified in *A. sol* for the biosynthesis of heme and riboflavin, both of which are major metabolites synthesized in plastids of plastid-bearing organisms. In addition, homologs for the biosynthesis of lysine, aromatic amino acids, and branched-chain amino acids, usually localized in plastids in photosynthetic eukaryotes, were not detected. It would be worth noting that the *A. sol* transcriptome data contain transcripts predicted as homologs for the biosynthesis of methionine from cysteine and of proline from arginine, all of which are highly likely localized in cytosol or mitochondria (Supplementary dataset S5, Supplementary Material online). We did not detect homologs for the biosynthesis of arginine, asparagine, and histidine in our data. Instead, we could detect transcripts for proteins involved in heme maturation, and for heme attachment to cytochrome *c* as well as cytochrome *c*-mediated mitochondrial respiration complexes (Supplementary datasets S5 and S6, Supplementary Material online), strongly suggesting heme is required for *A. sol*. Similarly, we detected transcripts for riboflavin conversion to flavin mononucleotide (FMN) and flavin adenine dinucleotide (FAD) as well as FMN- and FAD-dependent protein sequences (Supplementary datasets S5 and S6, Supplementary Material online). *A. sol* might be capable of effectively acquiring the metabolites from the prey. Dependence on extracellular heme, riboflavin, and particular amino acids is not uncommon in eukaryotes, such as the free-living nematode (Rao et al. 2005; Payne and Loomis 2006; Subramanian et al. 2011; Biswas et al. 2013).

As described above, *A. sol* possesses cytosolic, mitochondrial, and ER-localized pathways (fig. 2), homologous to the cytosolic pathways for isoprenoids (MVA pathway), cytosolic and mitochondrial glycolysis and pentose phosphate pathway, and the ER-localized lipid metabolisms, that the ochrophytes possess together with the plastid-localized pathways (e.g., Tanaka et al. 2015; Kamikawa et al. 2017). We detected the FASII fatty acid biosynthesis pathway transcripts for proteins, FabD, FabB/F, FabG, and FabI, which highly likely localize in the cytosol or mitochondria (fig. 2), in the *A. sol* transcriptome data. Although the phylogenetic analysis shows the *A. sol* FabD is grouped with plastid-targeted homologs of photosynthetic eukaryotes including Ochrophyta species, the bootstrap support for the relationship is low. In contrast, the other genes for fatty acid biosynthesis in *A*. *sol*, that is, the FabB/F, FabG, and FabI, are not specifically related to plastid-targeted homologs from Ochrophyta and other lineages with red alga-derived plastids (Supplementary figs. S4–S7, Supplementary Material online). As the FASII fatty acid biosynthesis in Ochrophyta has been replaced by endosymbiont-derived plastid-localized pathway (e.g., Kamikawa et al. 2017), the phylogenetic analyses suggest that the fatty acid biosynthesis in *A. sol* has never been replaced by the plastid counterpart.

### Gene Transfers of Organellar Aminoacyl-tRNA Synthase Genes

**Fig. 3.**
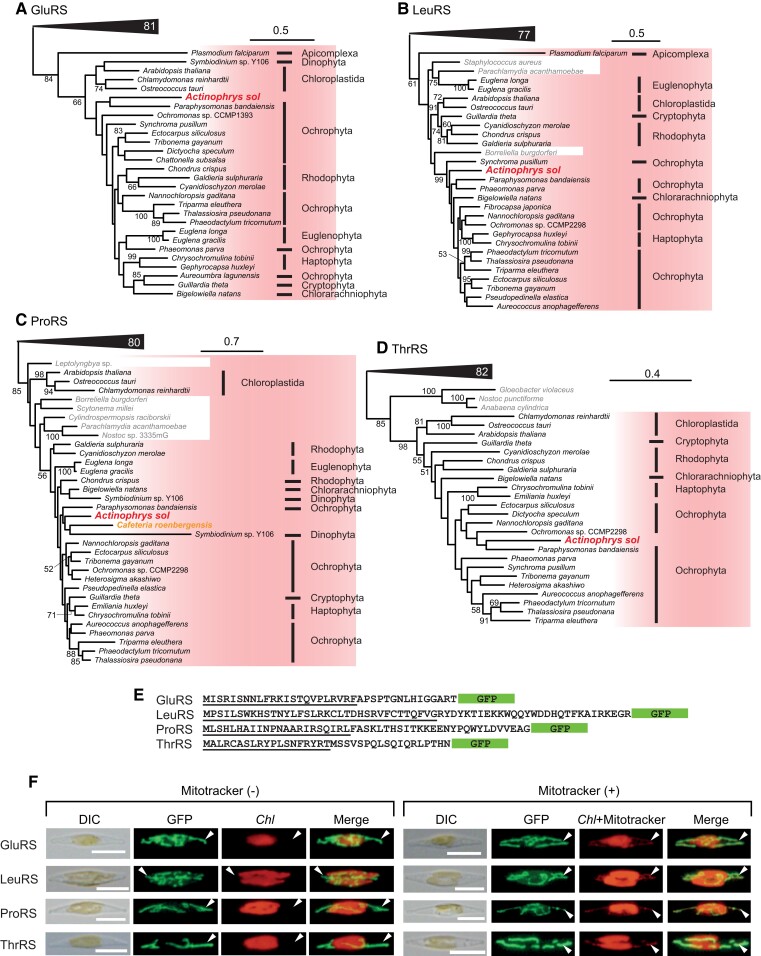
Evolution and localization of organellar aminoacyl-tRNA synthases in *A. sol*. (*A*) ML tree of GluRS. The “plastid clade” comprising plastid-bearing species and *A. sol* is highlighted. The numbers on branches represent bootstrap values. Only bootstrap values ≥50 are shown. Clades outside of our interest are collapsed as closed triangles and the numbers of taxa are indicated. Prokaryotic sequences are in light gray. Note that *Paraphysomonas* possesses a nonphotosynthetic plastid that does not contain DNA and its aaRSs of the PL-clade target to the mitochondria (Dorrell et al. 2019). (*B*–*D*) ML trees for LeuRS, ProRS, and ThrRS. *Cafeteria roenbergensis*, Opalozoa, is highlighted in orange in (*C*). Other details are as same as (*A*). (*E*) N-terminal amino acid sequences of *A. sol* aaRSs, C-terminally tagged by GFPs. The underlined regions are mitochondrial transit peptides predicted by the *in silico* analyses. (*F*) Localization of the recombinant GFPs. Arrowheads indicate compartments, in which the GFP recombinant proteins localize. GFP fluorescence is not colocalized with chlorophyll fluorescence in “Mitotracker (−)”, but colocalized with Mitotracker fluorescence in “Mitotracker (+)”, indicative of mitochondrial localization. DIC, differential interference contrast; Chl, chlorophyll autofluorescence; GFP, GFP fluorescence; Merge, a merged image of chlorophyll and GFP fluorescence. Scale bars in the pictures show 10 µm.

To further evaluate a past plastid in Actinophryidae, we reconstructed single-protein trees for the 158 protein sequences detected in the *A*. *sol* transcriptome during the survey of plastid proteins (see above). Although the phylogenetic positions of most of those sequences were placed in clades of cytosolic or mitochondrial homologs of other eukaryotes (e.g., see Supplementary figs. S8–S17, Supplementary Material online) or remained unclear due to low bootstrap supports, the seven trees show monophyletic groups exclusively comprising *A. sol*, Ochrophyta, and some other photosynthetic eukaryotes (hereafter referred to as “PL-clades”) with modest to high bootstrap supports (fig. 3; Supplementary figs. S18–S24, Supplementary Material online). These are not likely random gene transfers, but rather there appears to have been a certain selective pressure for *A. sol* to have gained and retained these genes, all of which code a single enzymatic group, organellar aaRSs such as AspRS, GluRS, GlyRS, LeuRS, SerRS, ThrRS, and ValRS. The organellar aaRSs in photosynthetic eukaryotes including those of Ochrophyta are reported to be dually targeted to both, plastids and mitochondria (Duchêne et al. 2005; Rokov-Plavec et al. 2008; Hirakawa et al. 2012; Gile et al. 2015; Dorrell et al. 2017). As the PL-clades are comprised of unrelated photosynthetic organisms and the eukaryvorous *A. sol*, they have highly likely been shaped by lateral/endosymbiotic gene transfers. Some of the PL-clades contain Chloroplastida, Rhodophyta, lineages with plastid derived from green alga, that is, chlorarachniophytes and euglenophytes, and lineages possessing plastid derived from red alga, although internal relationships in all the PL-clades are not well resolved due to low bootstrap supports. Given the organellar aaRSs of PL-clades present in the sister lineages, *A. sol* (Actinophryidae) and Ochrophyta, the genes might have been present in their last common ancestor, based on parsimony logic.

We then carefully checked the other trees for aaRSs. In AsnRS, *A. sol*, Ochrophyta, and some other photosynthetic eukaryotes were also reconstructed as monophyletic but the bootstrap support was low (Supplementary fig. S25, Supplementary Material online). Phylogenetic analyses of six aaRS, namely ArgRS, IleRS, LysRS, MetRS, TrpRS, and TyrRS, show that sequences from *A. sol*, Ochrophyta, some other photosynthetic eukaryotes, and bacteria form a clade with low to high bootstrap values (Supplementary figs. S26–S31, Supplementary Material online). We did not detect CysRS and PheRS members of PL-clades in the *A. sol* transcriptome data, but instead, *A. sol* possesses the genuine mitochondrial-targeted counterparts (Supplementary figs. S32 and S33, Supplementary Material online), consistent with the genuine FASII pathway genes (Supplementary figs. S4–S7, Supplementary Material online). We detected second copies of AlaRS and HisRS in addition to the cytosolic homologs (Supplementary figs. S34 and S35, Supplementary Material online), but their phylogenetic positions remain unclear. Although we conducted in-depth survey for the aaRS homologs of the PL-clades in the heterotrophic lineages of the Stramenopiles including Pseudofungi, no candidate was identified but only genuine mitochondrial aaRSs distantly related to the “PL-clades” were detected (Supplementary figs. S18–S35, Supplementary Material online). We did detect one exception, the ProRS of the deep-branching heterotrophic stramenopile *Cafeteria roenbergensis* groups in the PL-clade (fig. 3*C*; Supplementary fig. S36, Supplementary Material online).

In contrast to the dual-targeted organellar aaRSs of Ochrophyta (Gile et al. 2015), *A. sol* homologs were predicted to possess N-terminally mitochondrial-targeting sequences unless truncated (table 1; Supplementary dataset S1, Supplementary Material online); there is no detectable plastid-targeting sequences in those protein sequences as discussed above (fig. 3*E*; table 1; Supplementary dataset S1, Supplementary Material online). To examine their mitochondrial localization, the GFPs N-terminally tagged with the N-terminal region of each for GluRS, ProRS, LeuRS, and ThrRS were expressed in cells of the photosynthetic diatom *Phaeodactylum tricornutum*. We observed GFP fluorescence colocalized with the mitotracker signals outside the chlorophyll signals (figs 3*E* and *F*), strongly suggesting that the *A. sol* organellar aminoacyl-tRNA synthases have the potential to be exclusively targeted to mitochondria.

**Table 1.** Summary of the aaRS Gene Distribution in *Actinophrys sol* Detected from the Transcriptome Data.

aaRSs	Cyt-clade	Mt-clade	Unknown type	PL-clade	Localization^a^
**Ala**	+	ND	+	ND	−
**Arg**	+	ND	ND	+	Mt
**Asn**	+	ND	ND	+	5′ truncated
**Asp**	+	ND	ND	+	5′ truncated
**Cys**	+	+	ND	ND	−
**Gln**	+	ND	ND	ND	−
**Glu**	+	ND	ND	+	Mt
**Gly**	+	ND	ND	+	5′ truncated
**His**	+	ND	+	ND	−
**Ile**	+	ND	ND	+	5′ truncated
**Leu**	+	ND	ND	+	Mt
**Lys**	+	ND	ND	+	Mt
**Met**	+	ND	ND	+	5′ truncated
**Phe (alpha)**	+	+	ND	ND	−
**Phe (beta)**	+	ND	ND	ND	−
**Pro**	+	ND	ND	+	Mt
**Ser**	+	ND	ND	+	5′ truncated
**Thr**	+	ND	ND	+	Mt
**Trp**	+	ND	ND	+	5′ truncated
**Tyr**	+	ND	ND	+	Mt
**Val**	+	ND	ND	+	5′ truncated

note.—+, sequence included in the clade was detected; −, not analyzed; ND, not detected; Mt, mitochondrial-targeted. Mt underlined indicates the localization was experimentally confirmed.

a Predicted localization of the PL-clade sequences.

### Hypotheses of Early Plastid Evolution of Ochrophyta

**Fig. 4.**
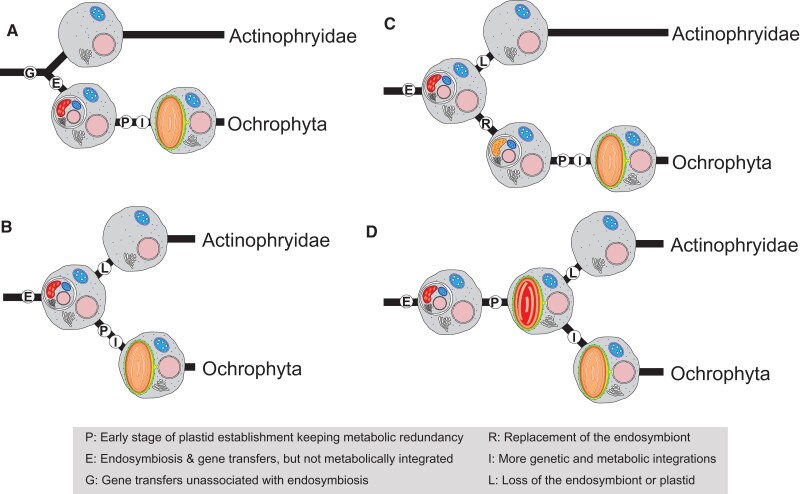
Possible scenarios for early plastid evolution in Ochrophyta. (*A*) Detected plastid-related genes (e.g., aaRS) found in *A. sol* are derived from independent lateral gene transfers not associated with endosymbiosis of a photosynthetic eukaryote. After divergence from Actinophryidae, an ancestor of Ochrophyta has obtained a plastid. (*B*) Plastid-related genes (e.g., aaRS) found in *A. sol* are derived from endosymbiotic gene transfers occurred in the common ancestor of Actinophryidae and Ochrophyta. The endosymbiont gave rise to the fully integrated ochrophyte plastid. In contrast, the ancestor of Actinophryidae has lost the endosymbiont. (*C*) This is the similar scenario with (*B*). However, the endosymbiont has been replaced by a new one that gave rise to current ochrophyte plastid. (*D*) The common ancestor of Actinophryidae and Ochrophyta has possessed a plastid, followed by loss of a plastid in the evolution of Actinophryidae. However, the host cell in the common ancestor of Actinophryidae and Ochrophyta has retained both host-derived genuine genes and metabolic pathways in addition to their corresponding plastid-derived metabolic pathways and genes.

Together, our findings suggest the following four evolutionary scenarios for the establishment of Ochrophyta plastid. A certain portion of genes related to plastid biogenesis such as aaRSs were acquired in the last common ancestor of Actinophryidae and Ochrophyta. If Actinophryidae is sister to Ochrophyta as indicated by our phylogenomic analysis (fig. 1), the gene transfers might have been derived from lateral gene transfers not associated with endosymbiosis (fig. 4*A*) or by endosymbiotic transfers from a photosynthetic endosymbiont (fig. 4*B* and *C*). Even if a photosynthetic endosymbiont as a source of genes such as genes for certain aaRSs (fig. 3) was present inside the host cell of the last common ancestor of Actinophryidae and Ochrophyta, it remains unclear whether the endosymbiont was of the same origin as the current Ochrophyta plastids (fig. 4*B*) or was replaced by another one in the last common ancestor of Ochrophyta (fig. 4*C*). The later scenario is consistent with previous reports that many plastid-bearing eukaryotes possess nuclear-encoded genes for plastid functions and biogenesis that do not share an origin with the current plastid (Moustafa et al. 2009; Maruyama et al. 2011; Burki et al. 2012). This is rationalized by the “shopping bag hypothesis” (Larkum et al. 2007; Howe et al. 2008) and/or the “red-carpet hypothesis” (Ponce-Toledo et al. 2019), which propose that such genes have been transferred from the ancient endosymbiont discarded later or were results of lateral gene transfers not associated with endosymbiosis.

Otherwise, the last common ancestor of Actinophryidae and Ochrophyta might have possessed a plastid followed by its loss in an ancestor of Actinophryidae (fig. 4*D*). This could explain why many mitochondrial aaRS genes of *A. sol* are of PL-type, and not genuine mitochondrial ones. However, in addition to the algal genes, we have detected the genuine host-derived genes for FASII and some organellar aaRS in *A. sol* (figs. 2 and 3; Supplementary figs. S4–S7, S32, and S33, Supplementary Material online), genes of which Ochrophyta homologs have been replaced by plastid-targeted ones. This observation can be rationalized by metabolic and genetic redundancy kept in the last common ancestor of Actinophryidae and Ochrophyta, redundancy which has been eliminated in the current plastids of Ochrophyta (Kroth et al. 2008; Prihoda et al. 2012). A similar assumption was previously made for the plastid evolution in Archaeplastida and Picozoa (Schön et al. 2021). Thus, if true, *A*. *sol* might be the second free-living candidate that has lost a plastid, following Picozoa (Schön et al. 2021), because all other eukaryotes that have lost a plastid are parasitic (Abrahamsen et al. 2004; Gornik et al. 2015; Janouškovec et al. 2019). It is worth noting that the last scenario (fig. 4*D*) stands regardless of whether Actinophryidae is sister to or is nested within Ochrophyta. Our implication does not directly rule out the possibility that some of the PL-type aaRS genes were acquired even before divergence of Ochrophyta, Actinophryidae, and Pseudofungi, given the ProRS of PL-clade in the opalozoan *C. roenbergensis*. Besides, some genes in Pseudofungi are phylogenetically related to photosynthetic organisms (Tyler et al. 2006). Nevertheless, it would be worth noting that the “algal genes” in Pseudofungi are phylogenetically distantly related to those of Ochrophyta and not directly related to the plastid functions (Stiller et al. 2009; Wang et al. 2017). Therefore, the gene transfers so far found in Pseudofungi are not directly related to the acquisition of the Ochrophyta plastids (Wang et al. 2017).

Our findings in this study of *A. sol* demonstrate that acquisition of algal genes related to plastid biogenesis does not necessarily result in establishment and/or retention of a plastid. In addition to *A. sol*, plastid-lacking eukaryotes closely related to other plastid-bearing lineages might have similar evolutionary backgrounds, for example, Picozoa and Alveolata (Waller et al. 2016; Schön et al. 2021). There are numerous eukaryotes that have not been studied genomically, including eukaryotes known only from environmental DNA sequences. Gathering more transcriptomic and genomic data from such eukaryotes would provide more information regarding the principles of plastid organellogenesis and expand our knowledge on plastid evolution that began more than a billion years ago and has subsequently underpinned the global ecology of this planet.

## Materials and Methods

Details of materials and methods are described in supplementary materials, Supplementary Material online.

## Supplementary Material

msac065_Supplementary_Data

## Data Availability

DNA sequence reads and RNA sequence reads of the *A. sol* culture were deposited to DNA Data Bank of Japan (DDBJ) under the accession numbers PRJDB12218 and PRJDB12217, respectively. RNA sequence reads of *C. capillatum* and mitochondrial DNA sequence of *A. sol* were also deposited to DDBJ under the accession numbers PRJDB12219 and LC650202, respectively. We have also deposited the phylogenomic trees in Newick format, the trimmed and untrimmed alignments ortholog alignments, the phylogenomic matrix, and the single ortholog trees onto figshare (https://doi.org/10.6084/m9.figshare.19514266.v2).
