# Supplementary material for: An Enigmatic Stramenopile Sheds Light on Early Evolution in Ochrophyta Plastid Organellogenesis
Source: Mol Biol Evol. 2022 Mar 28;39(4):msac065. doi: 10.1093/molbev/msac065 (PMC9004409; doi:10.1093/molbev/msac065)
Supplement: msac065_Supplementary_Data [file msac065_supplementary_data.zip › SI_MBE_220121.pdf]

Supplementary information

Materials and methods

Isolation, cultivation and extraction of DNA and RNA

A. sol NIES-2497 was cultivated in medium containing 46.2 mM NaCl, 0.9 mM KCl, 0.8 mM $CaCl_2 \cdot 2H_2O$, 2.3 mM $MgCl_2 \cdot 6H_2O$, 0.6 mM $NaHCO_3$, 1.22 mM sodium acetate, 0.01% polypeptone, 0.02% tryptone, and 0.02% yeast extract, with the prey green alga *Chlorogonium capillatum* NIES-3374 under the laboratory conditions at room temperature. DNA of the *A. sol* culture was extracted with Plant DNA extraction kit (Jena Biosciences) according the manufacturer's instruction. RNA of the *A. sol* culture was extracted with Trizol (Thermo Fisher Scientific) and Phasemaker (Thermo Fisher Scientific) according to the manufacturer's instructions. *C. capillatum* was also cultivated independently in medium containing 12.19 mM sodium acetate, 0.1% polypeptone, 0.2% tryptone, 0.2% yeast extract, and 0.01 mg/ml $CaCl_2 \cdot 2H_2O$, under the laboratory conditions at room temperature under the 12 hours light: 12 hours dark conditions. RNA was extracted from *C. capillatum* as described above.

DNA sequencing, assembly, and detection of organellar DNA contigs

DNA was sent to Hokkaido System Science Co. (Japan) for the library preparation followed by sequencing with HiSeq2500 (Illumina), resulting in 44.5 million paired-end reads. Adapter trimming and quality filtering were performed with FASTX-Toolkit (http://hannonlab.cshl.edu/fastx_toolkit/). In quality filtering, reads with quality scores > 20 for at least 75% of their length were retained if longer than 50 bp in length, resulting in 30.7 million paired-end reads. The paired-end reads were assembled with SPAdes 3.10.0 (Bankevich et al. 2012) with the default settings. Organellar DNA-derived contigs were surveyed with the homology-based search (Altschul et al. 1997) under the e^{-5} e-value criterion using the plastid and mitochondrial DNA sequences of the diatom *Phaeodactylum tricornutum* (GenBank accession number: NC_008588 and NC_016739) and the green algae *Chlorogonium* spp. (GenBank accession numbers: KT625085-KT625091, Y07814, and Y13644) as queries. Detected contigs were then confirmed to be actually organellar DNA-derived contigs by blastN searches (Altschul et al. 1997) against the GenBank nucleotide database. The single contig with ca. 53 kb in length and with

higher nucleotide sequence identity to the stramenopile mitochondrial DNAs was identified as the *A. sol* mitochondrial DNA. PCR and Sanger sequencing were conducted for the *A. sol* mitochondrial DNA to fill the gap between the 5’ and 3’ termini of the contig and to re-sequence nucleotide sequences of “N”s in the contig, resulting in the 53,041 bp-long circularly mapping sequence (DDBJ accession no. LC650202). Protein-coding genes and RNA genes were identified with Mfannot (Burger et al. 2013). Other organellar DNA-derived contigs showing high nucleotide identity to the green algal organellar DNAs were identified as plastid and mitochondrial DNA-derived sequences of the prey green alga. DNA sequence reads were deposited to DDBJ (PRJDB12218).

RNA sequencing, assembly, and removal of sequences from the prey alga

Total RNA of the *A. sol* culture was sent to Bioengineering Lab (Japan) for sequencing with NextSeq500 (Illumina), resulting in 20.2 million paired-end reads. Adapter trimming and quality filtering were performed with FASTX-Toolkit as described above, resulting in 15.4 million paired-end reads. The paired-end reads were assembled with Trinity v.2.8.5 (Haas et al. 2013) with the default settings. As the assembled contigs include sequences from both *A. sol* and the prey alga, we removed the prey alga-derived contigs. For preparation of the transcriptome data of the prey alga *Chlorogonium capillatum*, RNA extracted from the *Chlorogonium* culture was sent to Bioengineering Lab (Japan) for sequencing with NextSeq500 (Illumina), resulting in 24.0 million paired-end reads. Removal of adapter sequences and quality filtering were performed as described above, resulting in 18.5 million paired-end reads. The paired-end reads were assembled as for *A. sol*. We then performed blastN (Altschul et al. 1997) for each contig of the transcriptome data of the *A. sol* culture against the contigs of *Chlorogonium* transcriptome data. Contigs with more than 95% nucleotide identity to any of the *Chlorogonium* contigs were regarded as sequences from the prey alga. After removal of the prey algal sequences from the *A. sol* culture transcriptome data, 39,835 contigs remained. When the cut-off criterion was changed to 90% nucleotide identity, the same set of contigs was retained. We confirmed that the same procedures with the 95% nucleotide identity cut-off for the transcript sequences of the model diatom *P. tricornutum* (https://genome.jgi.doe.gov/portal/) against the contigs of *Chlorogonium*

removed no *P. tricornutum* transcripts. After the blast-based removal, we further checked if any contigs derived from *Chlorogonium* are still contaminated. We mapped the filtered reads of *Chlorogonium* onto the 39,835 contigs. Thirty-eight contigs were those mapped by the *Chlorogonium* reads and their blastx top 5 hits against the GenBank nr database were green algae. We regarded the 38 contigs were of *Chlorogonium* and removed them from the assembly, resulting in 39,797 contigs. GC contents of those contigs were calculated by SeqKit (Shen et al. 2016). For the following analyses, we used the transcriptome data from which the prey algal contigs were removed. Quality of the *A. sol* transcriptome data was assessed by BUSCO v2. (Simão et al. 2015). The RNA sequence reads of *A. sol* and *C. capillatum* were deposited to DDBJ (PRJDB12217 and PRJDB12219, respectively).

Transcriptome-based reconstruction of metabolic pathways and their localization

Plastid functions in Ochrophyta include glycolysis/gluconeogenesis, pentose phosphate pathway, assimilation of nitrite and sulfate, biosynthesis of aromatic amino acids, branched chain amino acids, iron-sulfur clusters, heme, chlorophyll, fatty acids, lipids, and vitamins. Sequences of proteins involved in the above functions in the diatom *P. tricornutum* and the land plant *Arabidopsis thaliana* were used as queries, and homology-based search was conducted by tblastN (Altschul et al. 1997) against the transcriptome contigs of *A. sol* with the e^{-5} e-value criterion. We also surveyed protein sequences of biogenesis, translation, and transcription of plastid DNA, plastid membrane transporters, and protein translocons as described above. We checked whether the detected sequences were actually homologues of the queries by blastP (Altschul et al. 1997) against the GenBank nr database. Amino acid sequences homologous to the Ochrophyta plastid proteins were then subjected to SignalP3.0 (Bendtsen et al. 2004), SignalP4.1 (Petersen et al. 2011), TargetP1.1 (Emanuelsson et al. 2000), Predotar 1.04 (Small et al. 2004), and Mitofates (Fukasawa et al. 2015) to investigate N-terminal signal peptides and/or mitochondrial-targeting transit peptides. Only sequences from which signal peptides and/or mitochondrial-targeting transit peptides were detected by two and more of the above algorithms were regarded as possessing the targeting peptide. Sequences with the N-terminal extensions containing signal peptides were additionally analyzed by ASAFind

(Gruber et al. 2015) to check whether they possess the bipartite Ochrophyta plastid-targeting sequence comprised of a signal peptide followed by a transit peptide-like region and the ASAFAP motif at the cleavage site as high confidence. We manually checked whether the N-terminal extensions containing signal peptides also possess transit peptide-like regions with phenylalanine, tryptophan, tyrosine, or leucine at the first position. Note that in this process, no sequence was confidently regarded as plastid-targeted sequences. All the detected sequences possessing only signal peptides lacking transit peptide-like regions and the ASAFAP motif in the above analysis were thus regarded as protein sequences functioning in the endoplasmic reticulum. Especially aaRSs in the PL-clades were additionally subjected to iPSORT (Bannai et al. 2002), WoLF PSORT (https://wolfpsort.hgc.jp/) and MitoProt II v1.101 (Claros and Vincens 1996).

For the mitochondrial metabolisms, the *A. sol* transcriptome data were subjected to the analyses by using the KEGG Automatic Annotation Server (Moriya et al. 2007) followed by the KEGG mapper (Kanehisa et al. 2012). We surveyed sequences involved in biogenesis, translation, and transcription of mitochondrial DNA, Tricarboxylic acid cycle, iron sulfur cluster assembly, fatty acid β-oxidation, glycine cleavage, mitochondrial protein translocons (TIM/TOM), and oxidative phosphorylation as representative mitochondrial functions. Detected sequences were also checked whether they are actually homologues of the mitochondrial proteins by blastP (Altschul et al. 1997) against the GenBank nr database. Mitochondrial homologues were subjected to TargetP1.1 (Emanuelsson et al. 2000), Predotar 1.04 (Small et al. 2004), and Mitofates (Fukasawa et al. 2015) to detect the N-terminal mitochondrial targeting transit peptide. If there were missing proteins in the detected mitochondrial pathways by the above procedure, their homologues in *P. tricornutum*, *A. thaliana*, and the yeast *Saccharomyces cerevisiae* were used as queries for tblastN (Altschul et al. 1997) against the *A. sol* transcriptome data under the e^{-5} e-value criterion. In the same procedure, major metabolic pathways in the cytosol and the endoplasmic reticulum were also surveyed. Sequences with neither signal peptide nor mitochondrial transit peptide but with homology to cytosolic proteins were identified as cytosolic protein sequences.

Additional survey of plastid sequences

Protein sequences encoded in the assembled transcriptome contigs were predicted by TransDecoder equipped in Trinity, resulting in 27,099 predicted proteins. At first, ASAFind (Gruber et al. 2015) was applied to those protein sequences, and 174 and 759 sequences were identified as sequenced possessing N-terminal bipartite plastid targeting sequences with “high confidence” and “low confidence,” respectively. All the 174 “high confidence” sequences possess phenylalanine at the +1 position from the predicted signal peptide cleavage site. However, of the 759 “low confidence” sequences only 284 possess phenylalanine, leucine, tryptophan, or tyrosine at the +1 position from the predicted signal peptide cleavage site. Thus, we only considered the 174 and 284 sequences with “high confidence” and “low confidence,” respectively. Then those possible plastid protein sequences were checked i) whether those sequences possess homologues in other organisms, ii) whether they do not lack N-terminal regions, and iii) whether internal regions between signal peptides and mature regions with $\geq$5 amino acids corresponding transit peptide-like regions are present, by comparing homologues of other organisms. The above procedures resulted in 43 and 78 of “high confidence” and “low confidence” plastid targeted sequences left, respectively. Although KEGG functional annotation, as performed above, assigned functions to 13 and 35 of “high confident” and “low confident” plastid targeted sequences, respectively, the assigned functions are not known for plastid metabolic pathways and biogenesis, suggesting they are unlikely of true plastid proteins but rather likely of artefactually detected sequences (Supplementary Dataset S4).

5’ RACE analyses

For obtaining 5’ terminal sequences coding the N-terminal regions, 5’-RACE analyses for *A. sol* mRNAs of FabD, FabF, and organellar tRNA synthases (GluRS, LeuRS, ProRS, ThrRS, LysRS, and HisRS) were conducted using a 5’ RACE system for rapid amplification of cDNA ends kit (Invitrogen), following the manufacturer’s instructions using the primers shown in Supplementary Dataset S7. Those N-terminal sequences were analyzed as described above.

Localization of organellar aminoacyl-tRNA synthases

A. sol nucleotide sequences coding the N-terminal regions including mitochondrial transit peptides of aminoacyl-tRNA synthases (aaRSs) for Glutamate, Leucine, Proline, and Threonine were amplified by RT-PCR assays. The expression vector pPHA-NR for each of the *A. sol* sequence C-terminally tagged with eGFP gene was constructed by EZ-Clone Kit following the manufacturer's instruction. Transformation to the diatom *Phaeodactylum tricornutum* was performed by the electroporation with NEPA21 (NEPAGENE) as described previously (Miyahara et al. 2013; Dorrell et al. 2019). *P. tricornutum* transformants growing in a zeocin-based selection medium were observed with an Olympus BX51 fluorescent microscope (Olympus) equipped with an Olympus DP72 CCD color camera (Olympus). Mitochondrial localizations were observed using MitoTracker Orange with a 575 nm filter by 530 to 550 nm excitation. GFP fluorescence was detected with a 510 to 550 nm filter by 470 to 495 nm excitation, and chlorophyll autofluorescence was detected with a 575 nm filter by 530 to 550 nm excitation. For the organellar LysRS, 5' RACE and additional PCRs followed by the Sanger sequencing were performed with the primers shown in Supplementary Dataset S7 as described above to obtain the complete or the almost complete sequences and investigate their N-terminal targeting sequences.

Phylogenomic dataset construction

The orthologs that comprise the final datasets used for the phylogenomic analyses were collected from the predicted proteome of *A. sol* and subsequently processed via tools provided in the software package PhyloFisher (Tice et al. 2021). Briefly, the predicted proteome of *A. sol* was searched for the presence of the 240 orthologs found in the provided starting database of PhyloFisher using the default route and settings of the *fisher.py* tool. These putative orthologs collected from the proteome of *A. sol* were then added to their corresponding homolog alignments found in PhyloFisher's provided starting database via the tool *working_dataset_constructor.py* with default settings. Homolog trees were constructed, and short sequences were eliminated using *sgt_constructor.py* with default settings. The resulting homolog trees were manually inspected using the tools *forest.py* and *ParaSorter* to insure correct ortholog selection and removal of any remaining contaminants. A final set of 239 genes (75,984 amino acid

sites) and 75 taxa was created using the tools *select_taxa.py*, *select_orthologs.py*, and *prep_final_dataset.py*. The final matrix used for concatenation-based phylogenomic analyses was created using *matrix_constructor.py* with default settings.

Phylogenomic analyses

An initial ML tree was inferred from the final phylogenomic matrix using IQ-TREE (v2.0-rc1) under the LG+C20+G4 model. This initial tree was then used as an input to infer a Posterior Means Site Frequency model in IQ-TREE using the ML model LG+C60+G4+PMSF. The ML tree has 100 Real Bootstrap replicates mapped onto the nodes inferred under the same model.

To test the effect of fast evolving and on the topology of our final ML tree the fastest evolving sites were removed in a stepwise fashion (step size = 10,000 amino acids) creating a new matrix after each step using the tool *fast_site_remover.py*. This was done to exhaustion creating 8 sub-matrices from the original. Maximum likelihood trees were inferred from each submatrix via IQ-TREE again with ML model LG+C60+G4+PMSF informed by an independent LG+C20+G4 model tree inferred using IQ-TREE per each dataset and 1000 ultra-fast bootstraps optimized using the -*bnni* option. Support for bipartitions of interest were examined via *examine_bipartitions.py*. A similar strategy was used to assess the effect of the most heterotachious sites on the topology of our final ML tree with the only difference being that calculations were performed, sites were removed, and submatrices were created using the tool *heterotachy.py*. Trees were inferred as above per each dataset.

The final set of ortholog-only alignments generated from the above phylogenomic dataset construction was used in coalescence-based phylogenomic analyses. Single gene trees were built using *sgt_constructor.py* with default settings. The final set of 239 ortholog-only trees were used as input for *astral_runner.py* with default settings.

Reconstruction of single-protein trees

The sequences of *A. sol* detected as homologous to the ochrophyte plastid proteins in the above procedures were aligned with eukaryotic and bacterial counterparts, including cytosolic or mitochondrial homologues using MAFFT with the L-INS-i method (Katoh

and Standley 2013) followed by manual removal of ambiguously aligned sites with BioEdit (Hall 1999). Sequences used for the datasets were retrieved from the GenBank nr database, the JGI database, MMETSP (Keeling et al. 2014) and EukProt (Richter et al. 2021). Each of the single protein datasets was subjected to the maximum likelihood phylogenetic analysis with IQ-TREE (Nguyen et al. 2015) v. 1.6.7 with 100 bootstrap analyses under the LG + F + G4 model. Prior to the phylogenetic analyses, as the sequences of the PL-clade AlaRS, ArgRS, AspRS, AsnRS, CysRS, GlyRS, LeuRS, MetRS, TrpRS, and TyrRS were fragmented, PCR and the Sanger sequencing were performed to gain continuous sequences for each of the genes. The primers used are shown in Supplementary Dataset S7.

Table S1 Coverages of organellar DNAs in *Actinophrys* and non-photosynthetic species of Ochrophyta

		**Coverages**		
**lineages**	Species	Mitochondrial DNA	Plastid DNA	References
**Actinophryidae**	*Actinophrys sol*	10.8	ND	This study
**Bacillariophyceae**	*Nitzschia* sp. NIES-3581	98.2	88.2	Kamikawa et al. 2015
**Chrysophyceae**	*“Spumella”* sp. NIES-1846	35.9	19.5	Dorrell et al. 2019
**Dictyochophyceae**	*Pteridomonas* sp. NIES-3357	196.2	180.4	Kayama et al. 2020

ND: not detected

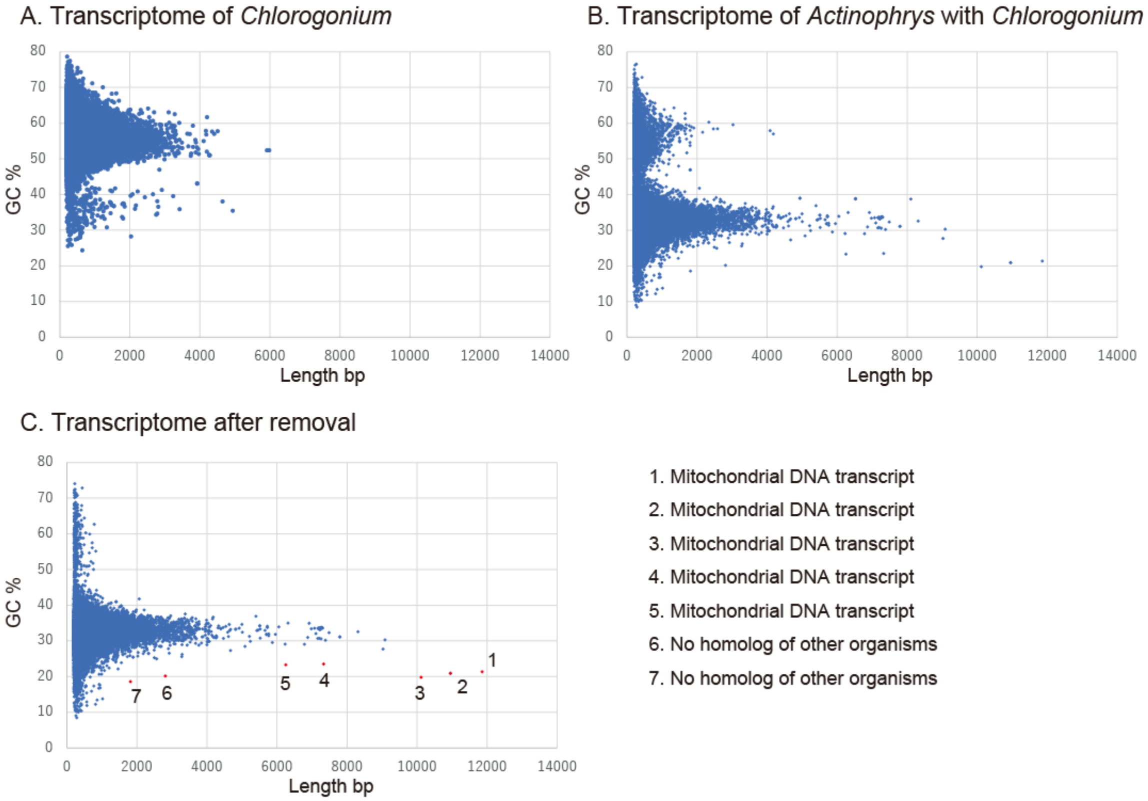

Fig. S1. GC contents for assembled contigs. A. Transcriptome of *Chlorogonium*. Each blue plot represents each assembled contig. B. Transcriptome of *Actinophrys* with *Chlorogonium*. C. Transcriptome after removal of contigs derived from *Chlorogonium*. We found contigs highlighted in red were of mitochondrial DNA-derived transcripts (no. 1-5) or unknown origins (no. 6 and 7).

Supplementary figures

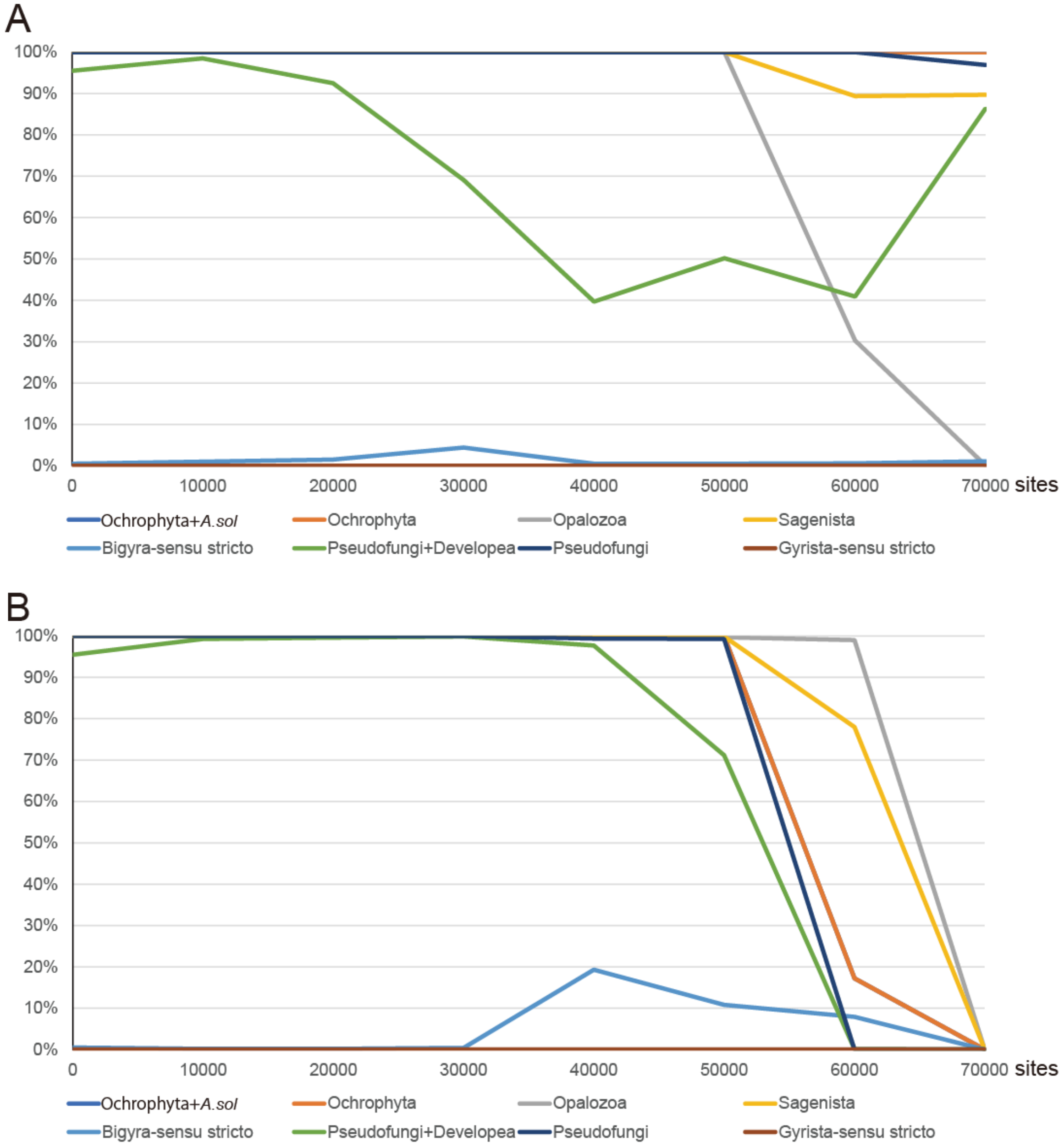

Fig. S2. Fluctuations in ultrafast bootstrap values for the monophyly of Ochrophyta and *A. sol*, the monophyly of Ochrophyta, the monophyly of Opalozoa, the monophyly of Sagenista, the monophyly of Pseudofungi, the monophyly of Pseudofungi and Developea, and other alternative relationships as a function of the proportion of heterotachious sites (A) and fast-evolving sites (B) removed from the phylogenomic dataset. Bigyra-sensu stricto comprises Opalozoa and Sagenista, and Gyrista-sensu stricto comprises Pseudofungi and Ochrophyta. Note that the line for the monophyly of Ochrophyta and *A. sol* and that for the monophyly of Ochrophyta completely overlap each other in both (A) and (B).

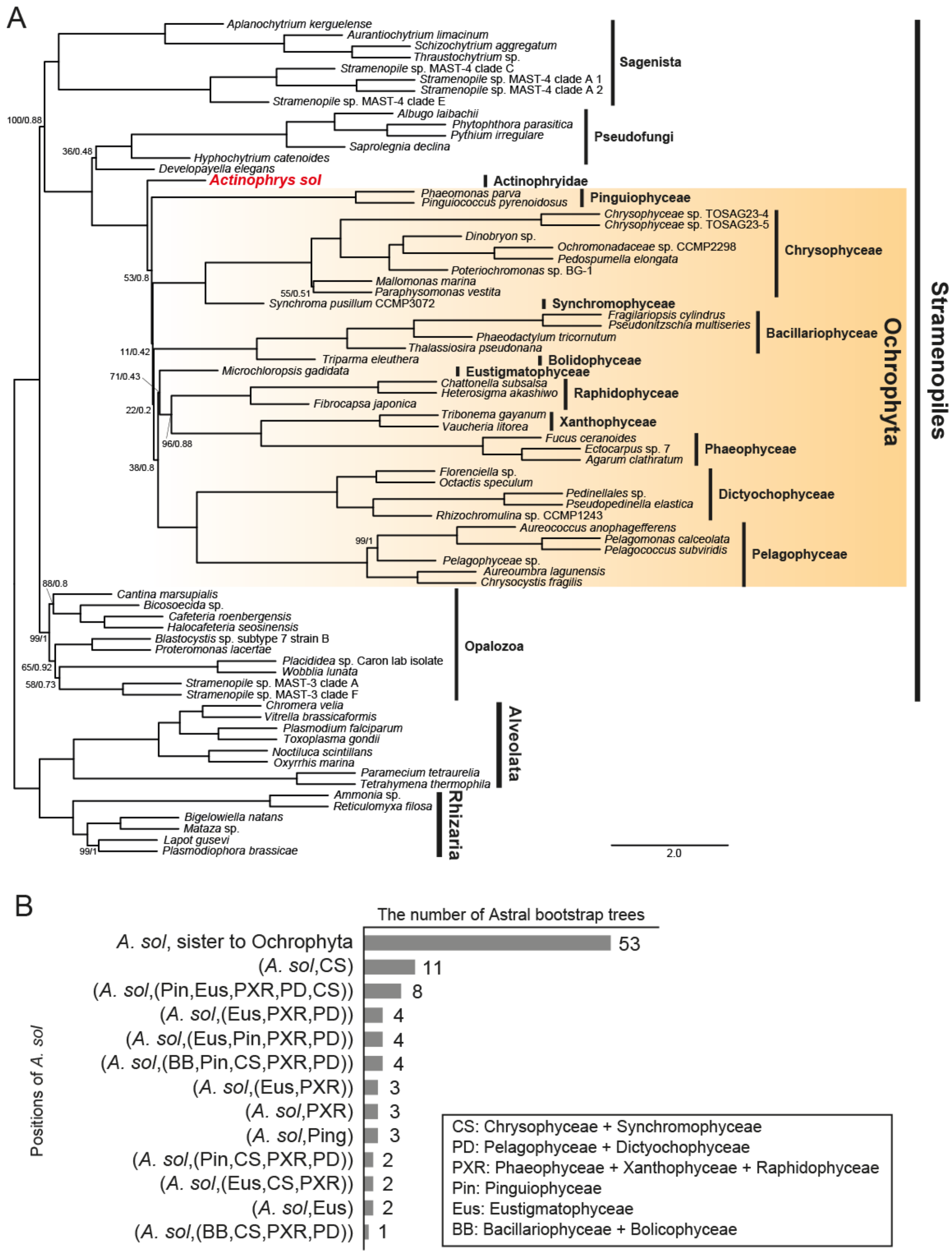

Fig. S3. Coalescence-based phylogenomic tree. A. Tree reconstructed by Astral. The numbers on branches are the bootstrap values (left) and the local posterior probabilities (right). If not shown, the nodes are fully supported. B. Bootstrap values of positions for *A. sol*.

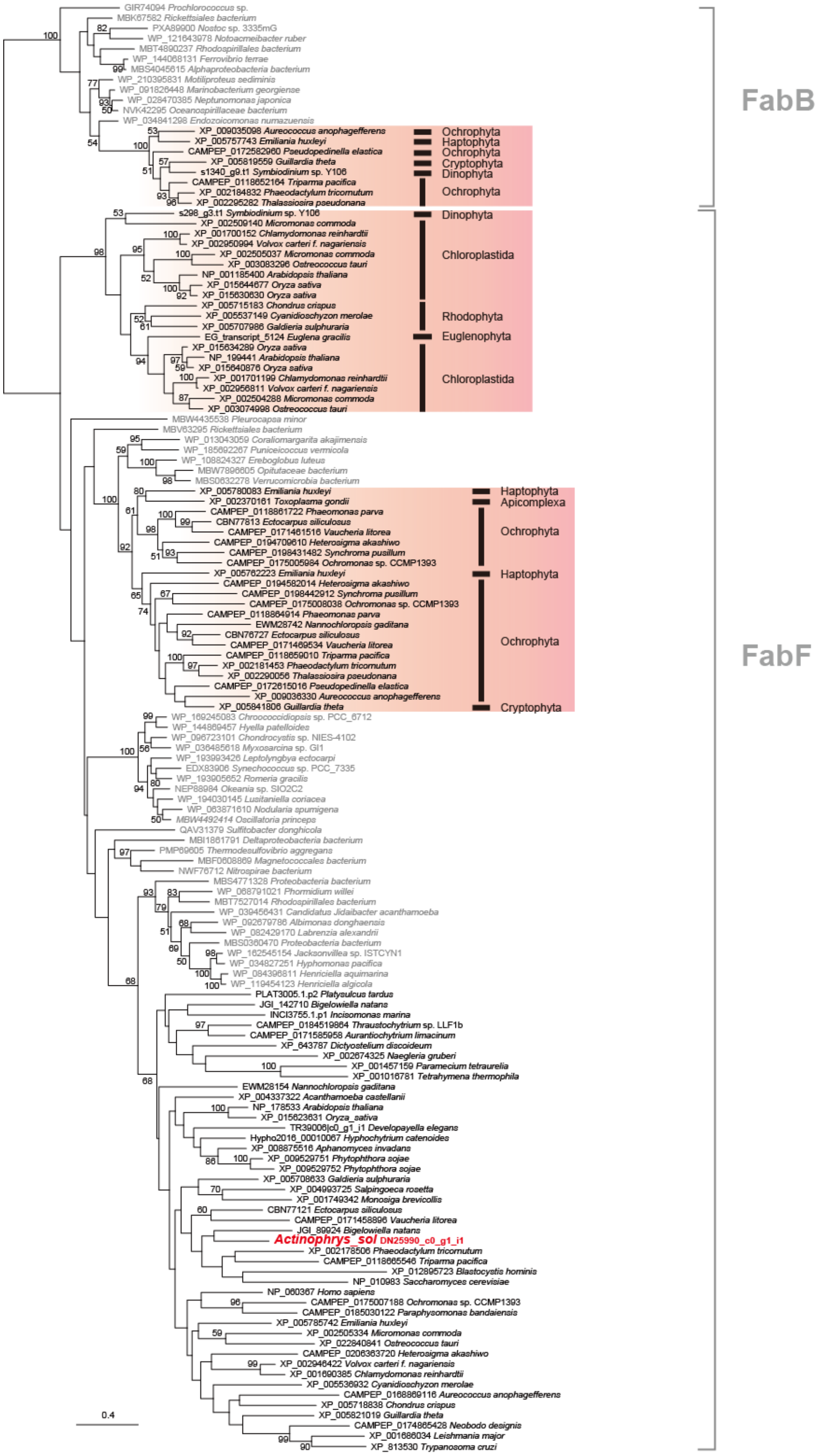

Fig. S4. Maximum likelihood tree of FabB and FabF. Maximum likelihood bootstrap values > 50% are shown on each node. Light red highlight indicates the plastid-targeted clade. Prokaryotic and eukaryotic taxa are in grey and black, respectively. *Actinophrys sol* is highlighted in red.

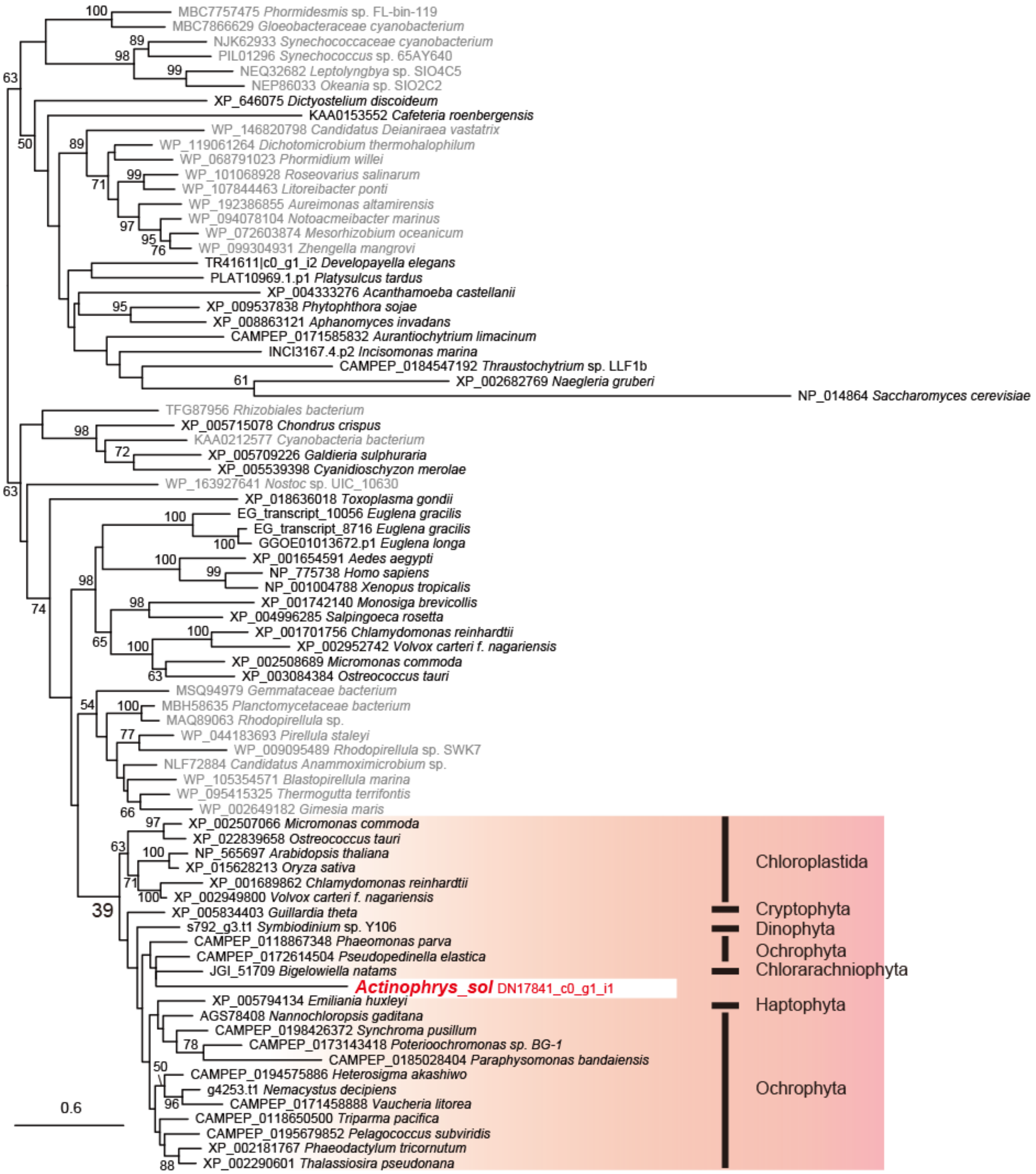

Fig. S5. Maximum likelihood tree of FabD. Maximum likelihood bootstrap values ≥ 50% are shown on each node. Light red highlight indicates the plastid-targeted clade. Prokaryotic and eukaryotic taxa are in grey and black, respectively. *Actinophrys sol* is highlighted in red.

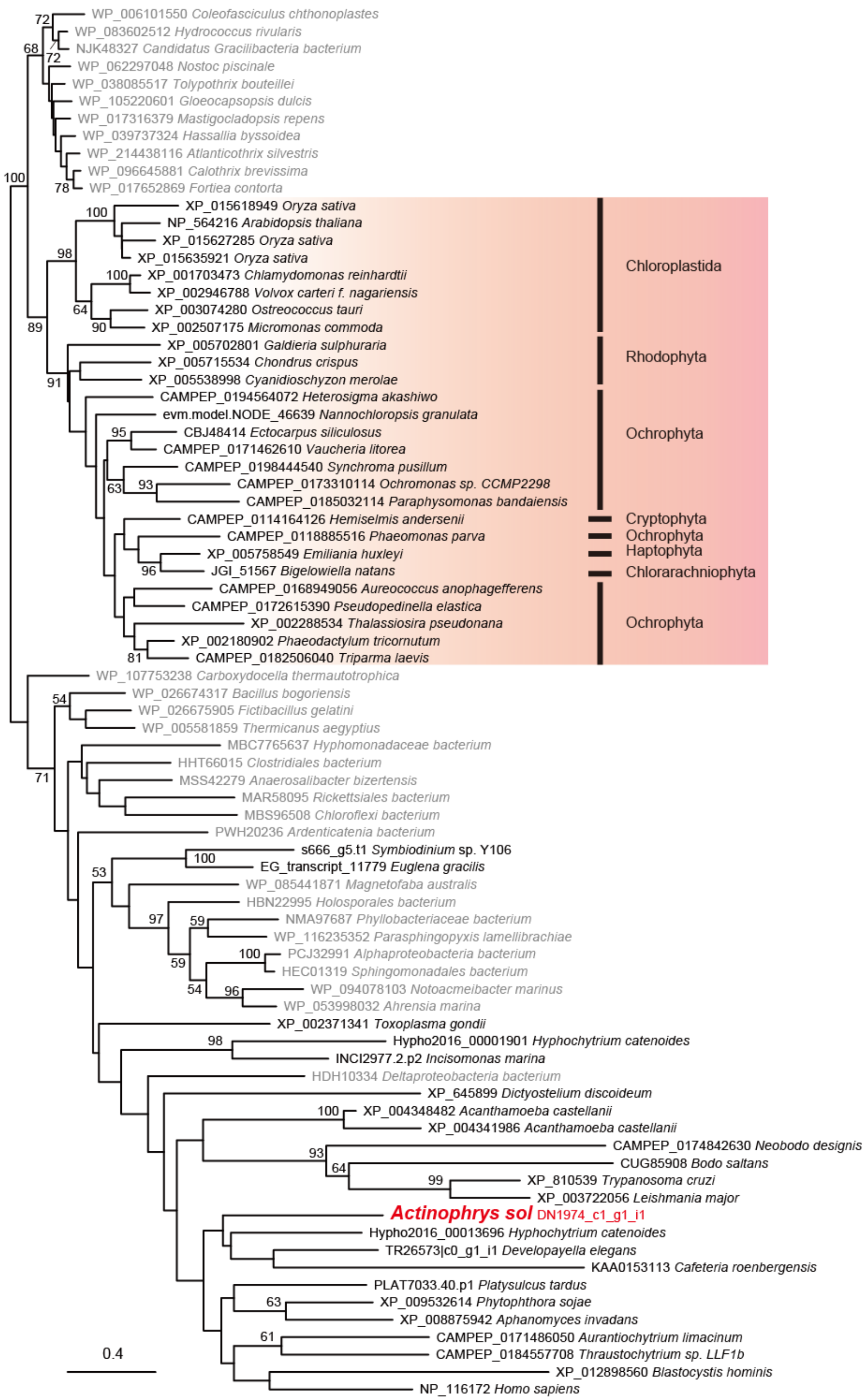

Fig. S6. Maximum likelihood tree of FabG. Maximum likelihood bootstrap values ≥ 50% are shown on each node. Light red highlight indicates the plastid-targeted clade. Prokaryotic and eukaryotic taxa are in grey and black, respectively. *Actinophrys sol* is highlighted in red.

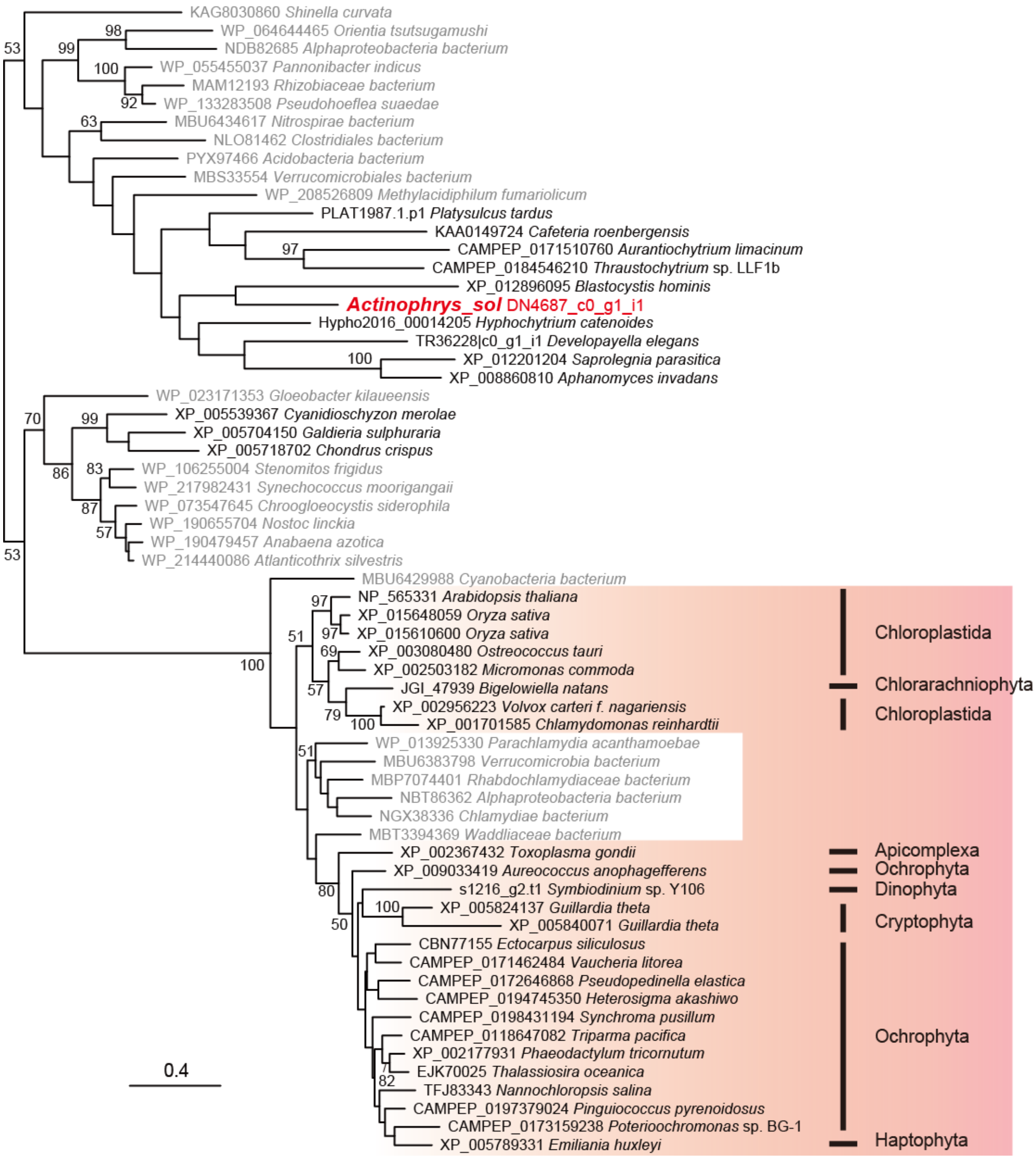

Fig. S7. Maximum likelihood tree of FabI. Maximum likelihood bootstrap values ≥ 50% are shown on each node. Light red highlight indicates the plastid-targeted clade. Prokaryotic and eukaryotic taxa are in grey and black, respectively. *Actinophrys sol* is highlighted in red.

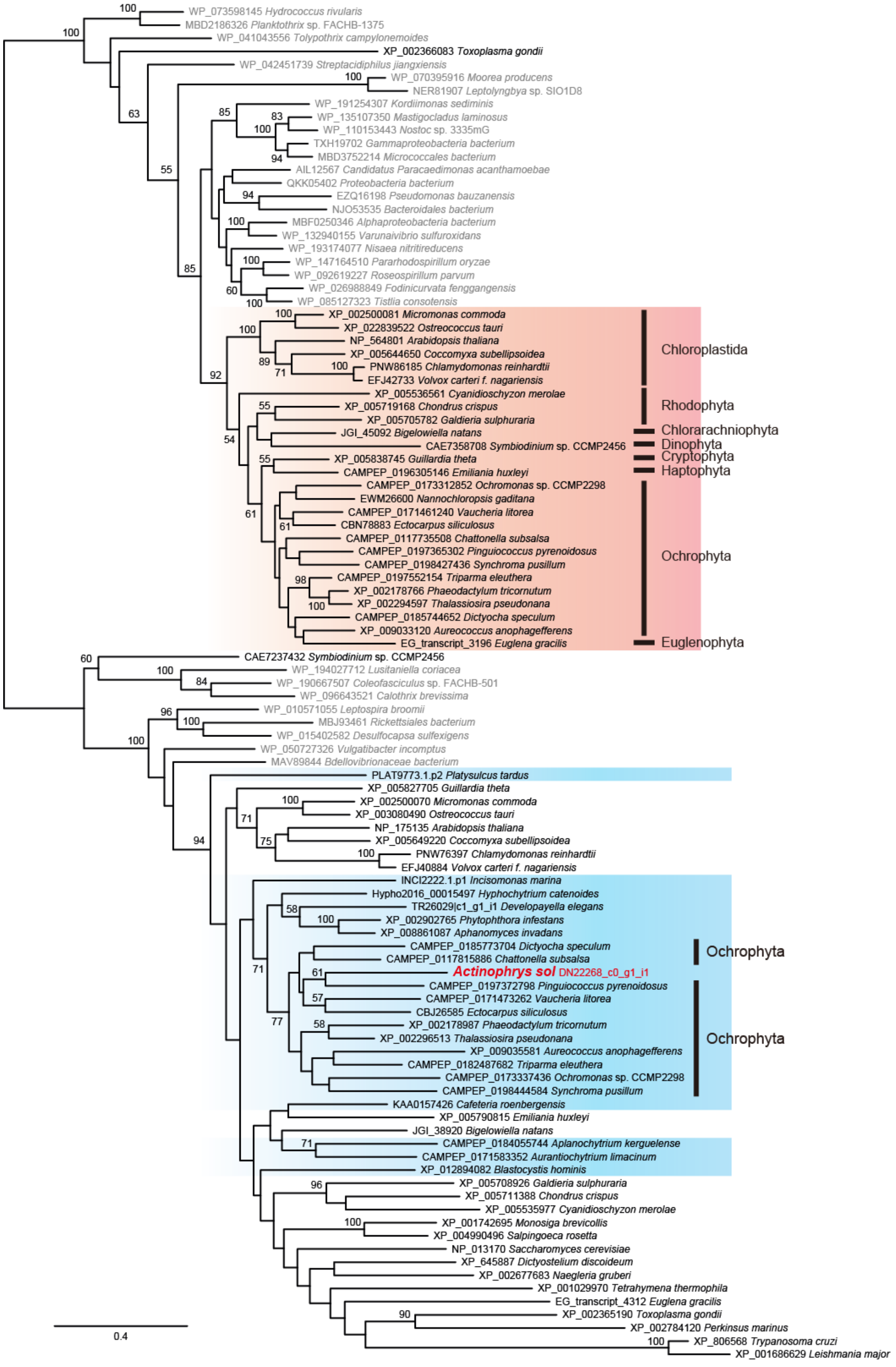

Fig. S8. Maximum likelihood tree of EF-G. Maximum likelihood bootstrap values ≥ 50% are shown on each node. Light red and light blue highlights indicate the plastid-targeted and mitochondrial-targeted sequences of the stramenopiles, respectively. Prokaryotic and eukaryotic taxa are in grey and black, respectively. *Actinophrys sol* is highlighted in red.

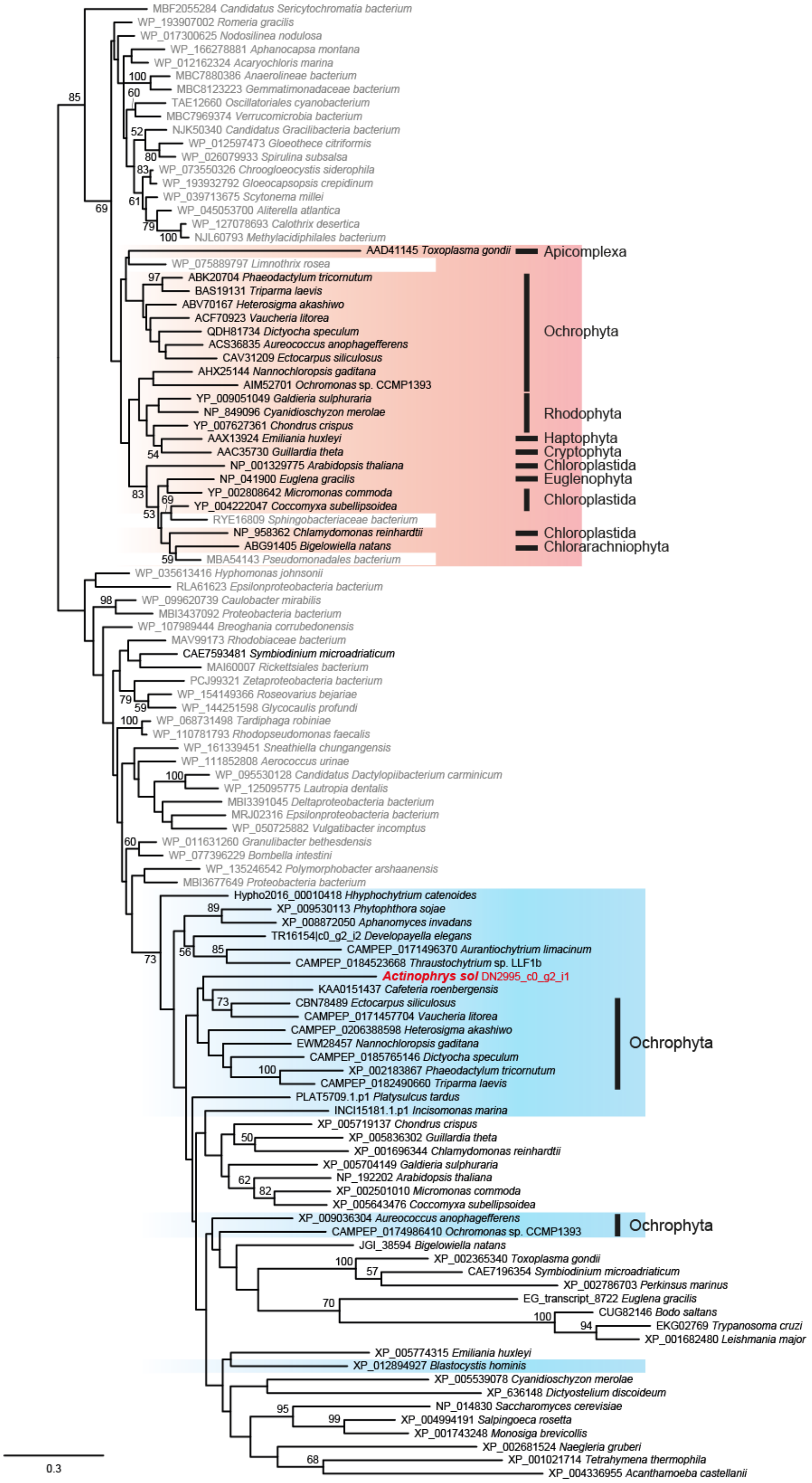

Fig. S9. Maximum likelihood tree of EF-Tu. Maximum likelihood bootstrap values ≥ 50% are shown on each node. Light red and light blue highlights indicate the plastid-targeted and mitochondrial-targeted sequences of the stramenopiles, respectively. Prokaryotic and eukaryotic taxa are in grey and black, respectively. *Actinophrys sol* is highlighted in red.

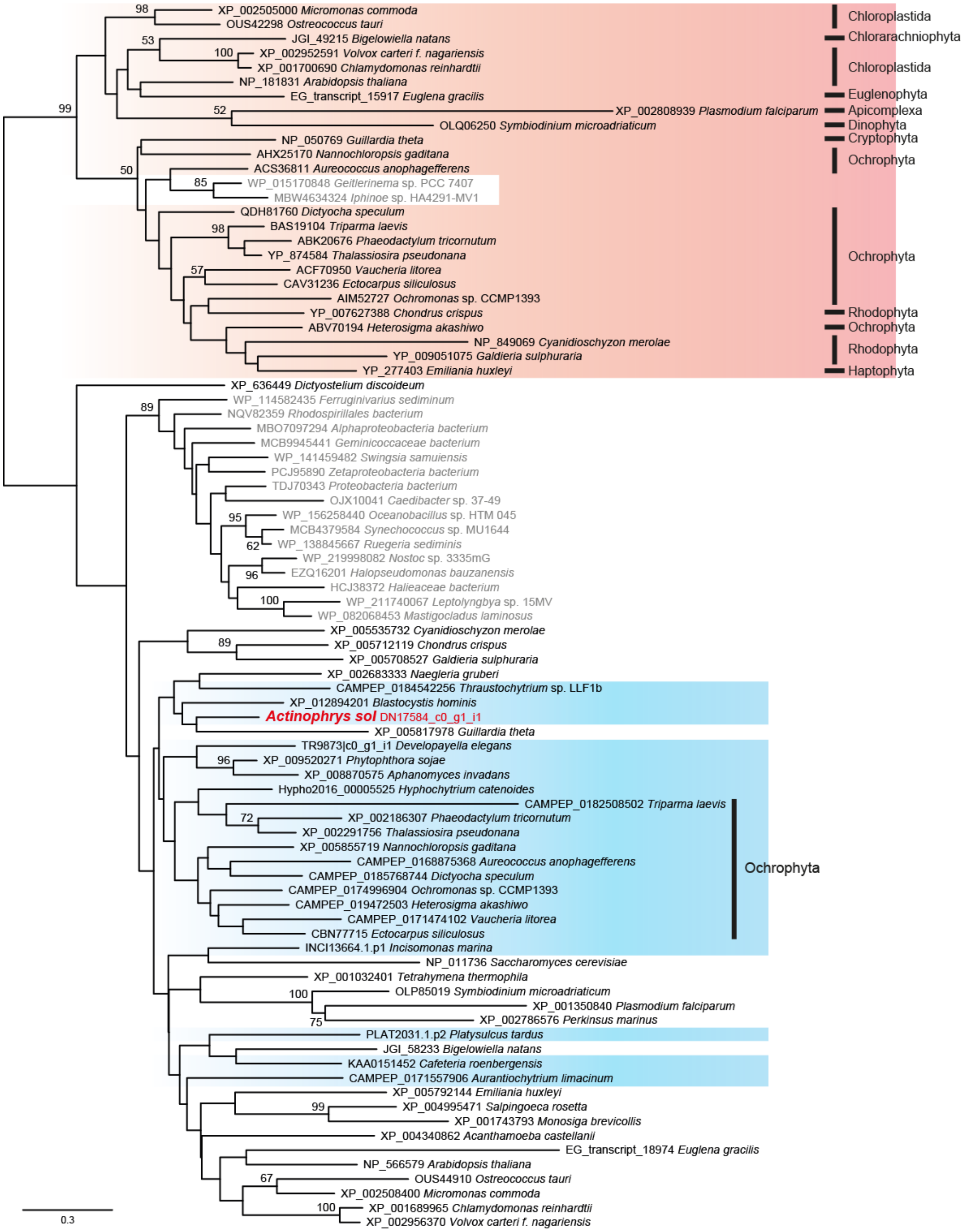

Fig. S10. Maximum likelihood tree of RPL3. Maximum likelihood bootstrap values > 50% are shown on each node. Light red and light blue highlights indicate the plastid-targeted and mitochondrial-targeted sequences of the stramenopiles, respectively. Prokaryotic and eukaryotic taxa are in grey and black, respectively. *Actinophrys sol* is highlighted in red.

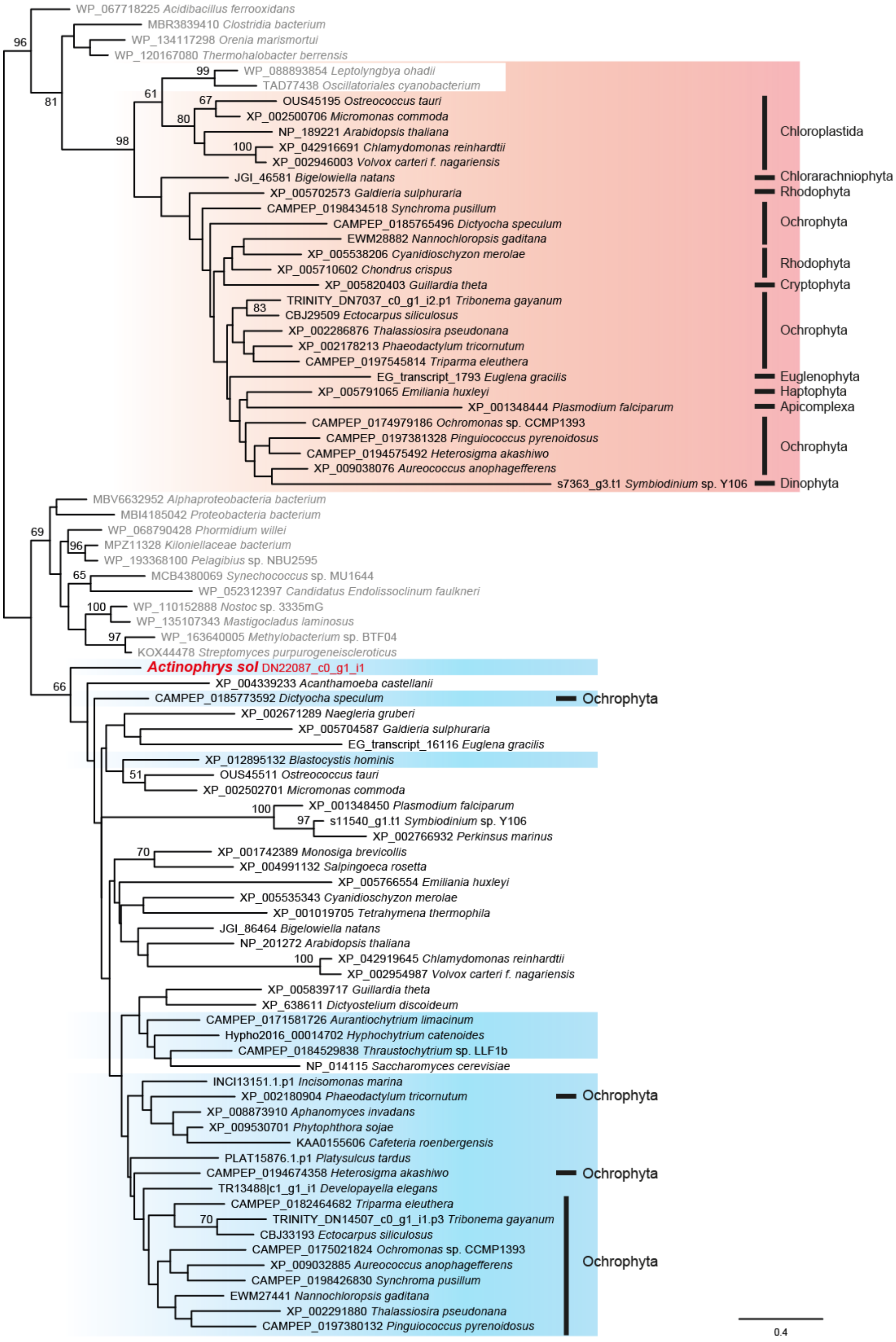

Fig. S11. Maximum likelihood tree of RPL15. Maximum likelihood bootstrap values $\geq$ 50% are shown on each node. Light red and light blue highlights indicate the plastid-targeted and mitochondrial-targeted sequences of the stramenopiles, respectively. Prokaryotic and eukaryotic taxa are in grey and black, respectively. *Actinophrys sol* is highlighted in red.

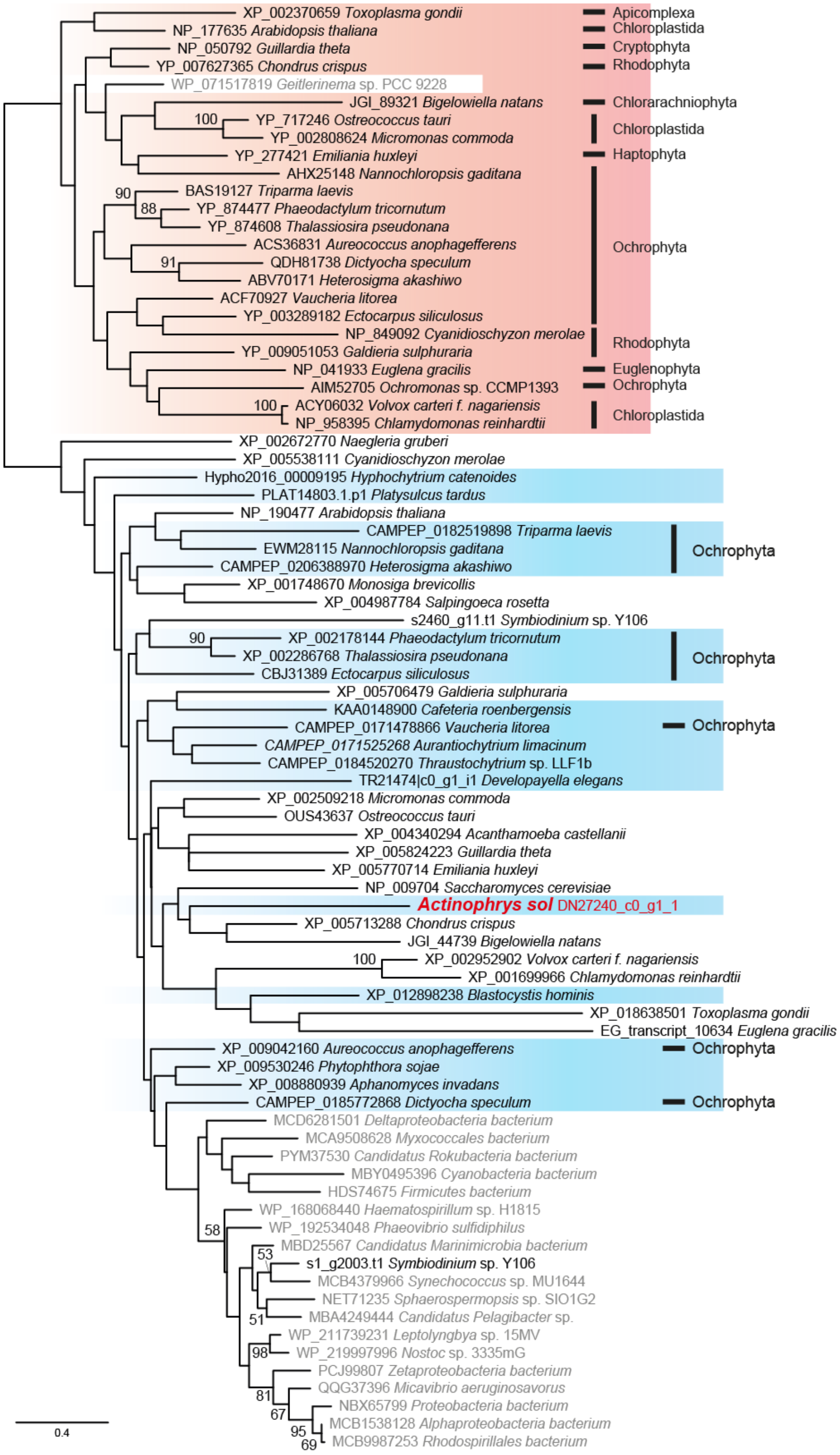

Fig. S12. Maximum likelihood tree of RPS9. Maximum likelihood bootstrap values ≥ 50% are shown on each node. Light red and light blue highlights indicate the plastid-targeted and mitochondrial-targeted sequences of the stramenopiles, respectively. Prokaryotic and eukaryotic taxa are in grey and black, respectively. *Actinophrys sol* is highlighted in red.

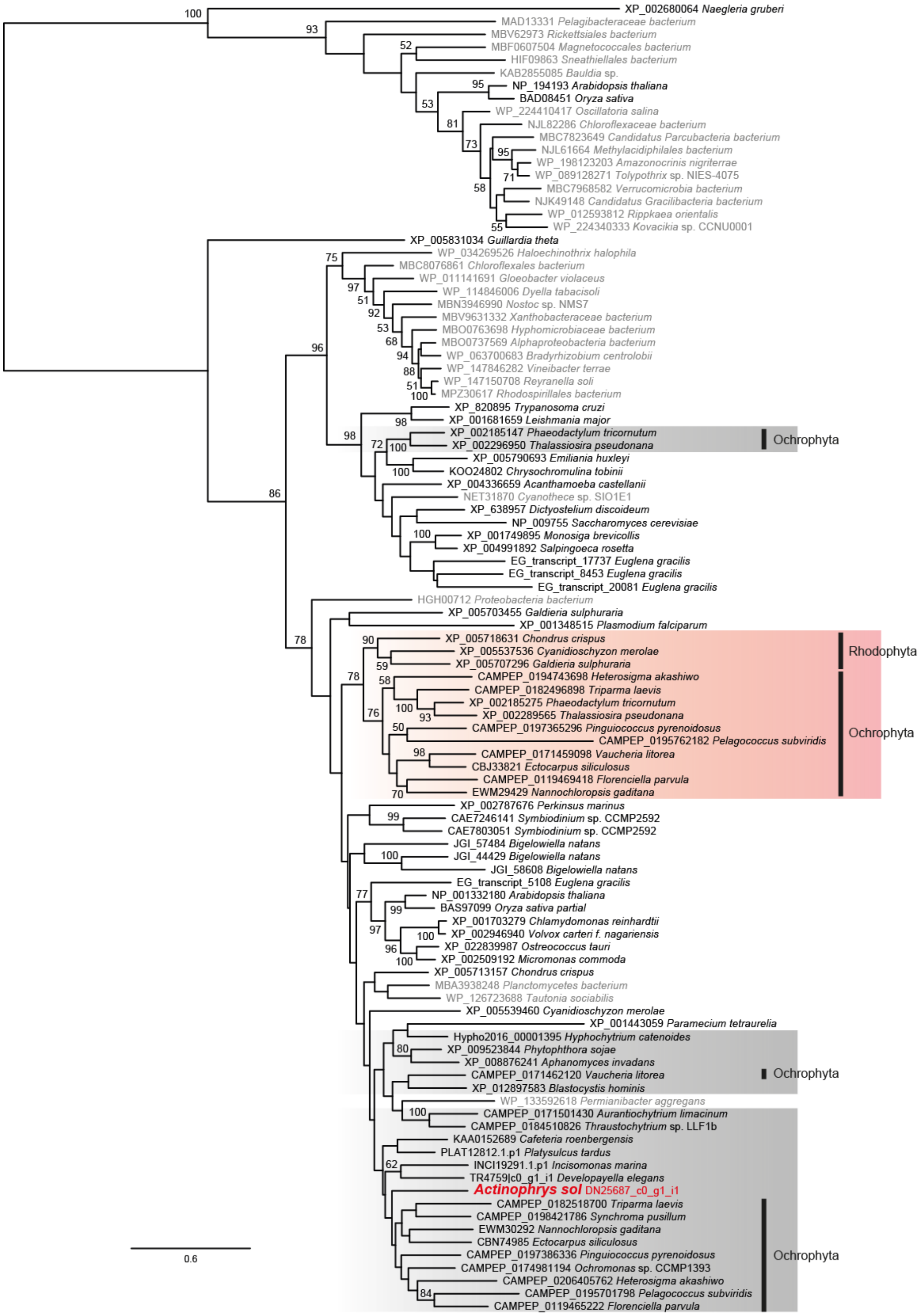

Fig. S13. Maximum likelihood tree of GPI. Maximum likelihood bootstrap values > 50% are shown on each node. Light red and light grey highlights indicate the plastid-targeted and cytosolic sequences of the stramenopiles, respectively. Prokaryotic and eukaryotic taxa are in grey and black, respectively. *Actinophrys sol* is highlighted in red.

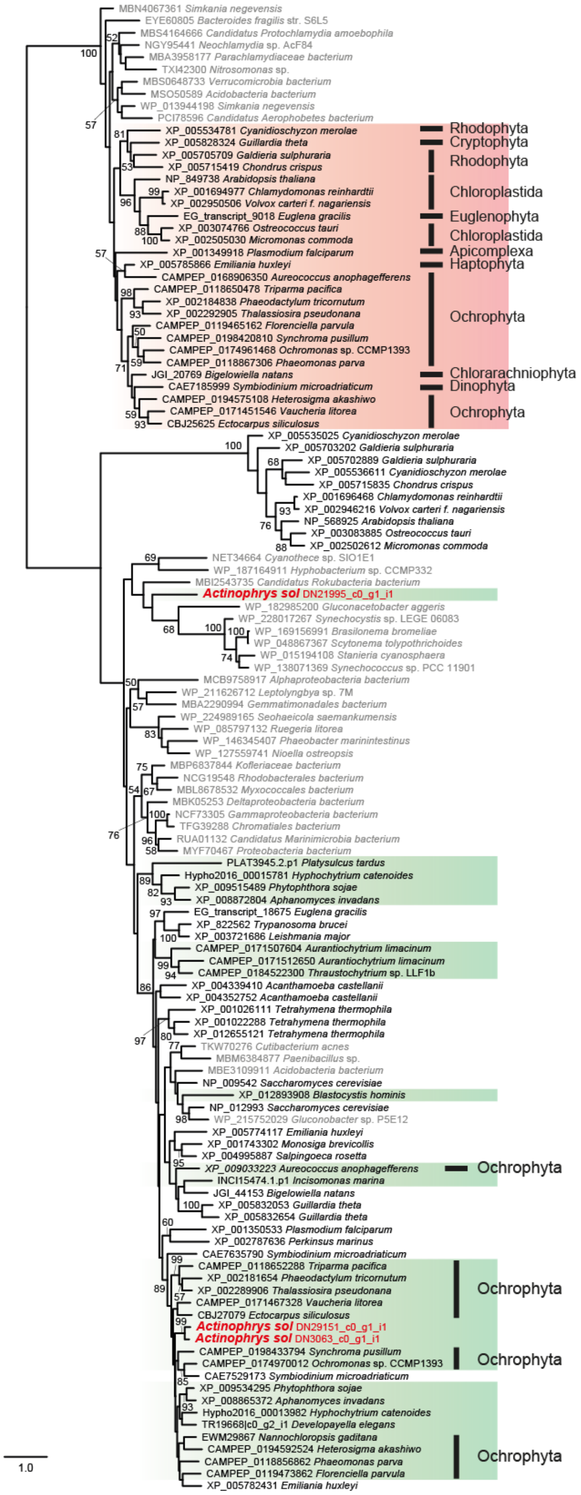

Fig. S14. Maximum likelihood tree of GPAT. Maximum likelihood bootstrap values $\geq$ 50% are shown on each node. Light red and light green highlights indicate the plastid-targeted and ER targeted sequences of the stramenopiles, respectively. Prokaryotic and eukaryotic taxa are in grey and black, respectively. *Actinophrys sol* is highlighted in red.

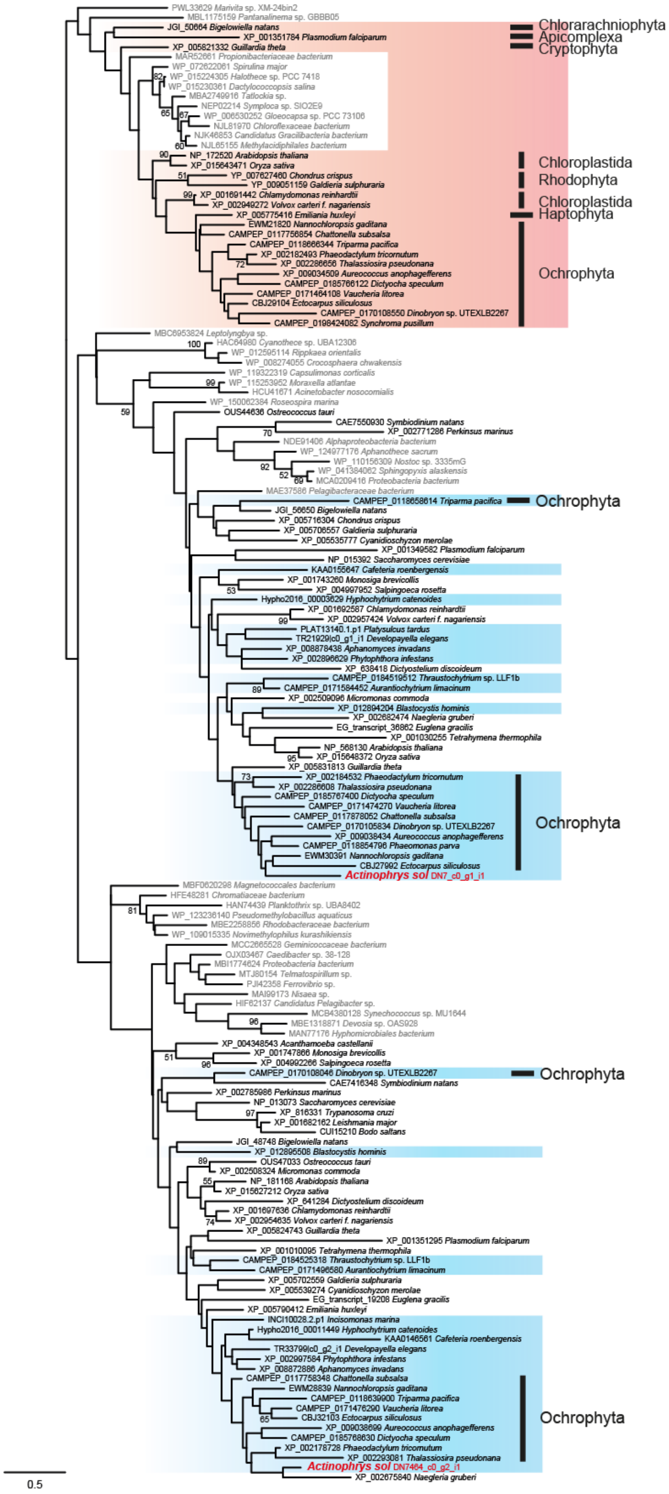

Fig. S15. Maximum likelihood tree of ISCA. Maximum likelihood bootstrap values ≥ 50% are shown on each node. Light red and light blue highlights indicate the plastid-targeted and mitochondrial targeted sequences of the stramenopiles, respectively. Prokaryotic and eukaryotic taxa are in grey and black, respectively. *Actinophrys sol* is highlighted in red.

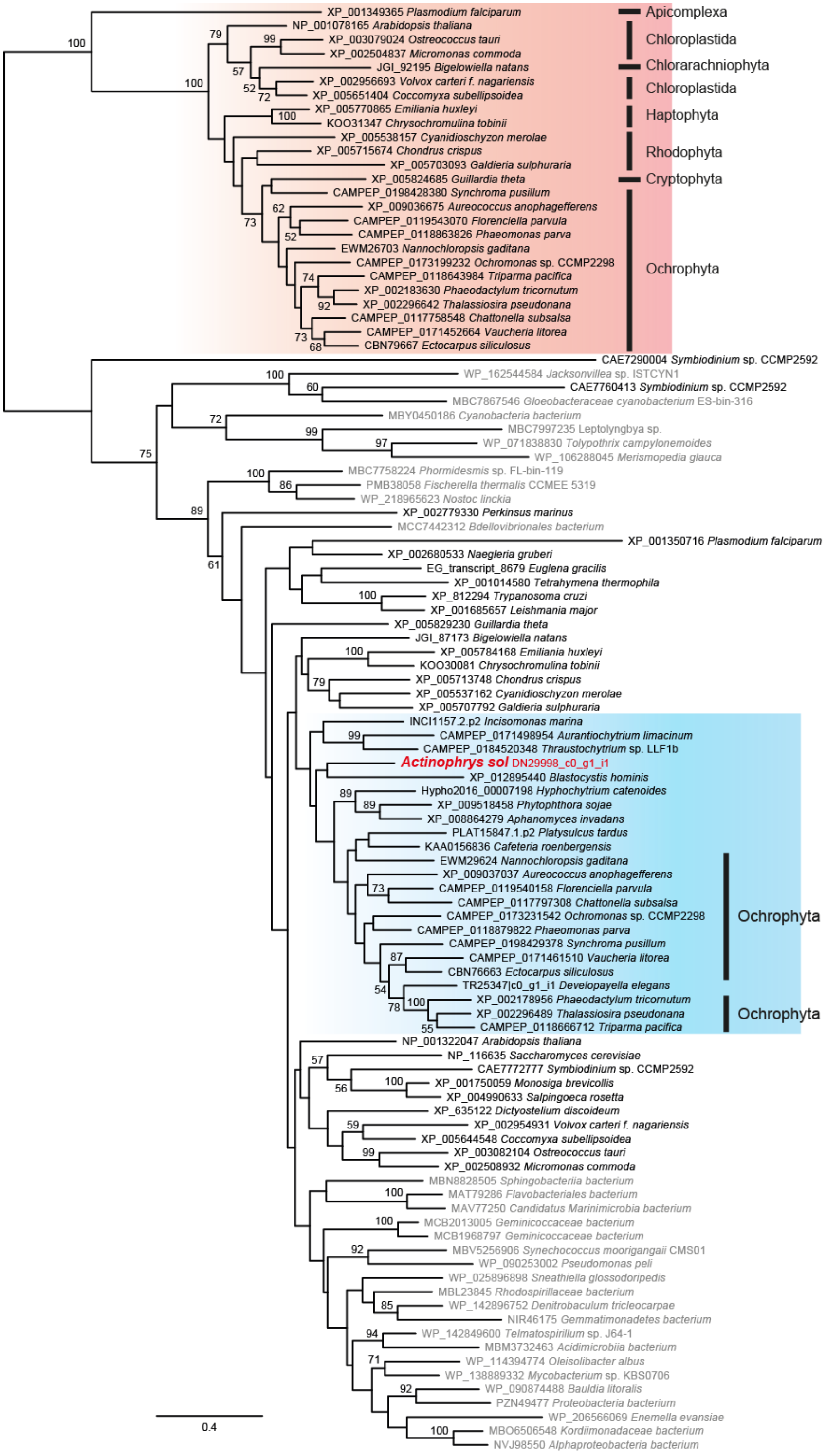

Fig. S16. Maximum likelihood tree of PDHE3. Maximum likelihood bootstrap values ≥ 50% are shown on each node. Light red and light blue highlights indicate the plastid-targeted and mitochondrial targeted sequences of the stramenopiles, respectively. Prokaryotic and eukaryotic taxa are in grey and black, respectively. *Actinophrys sol* is highlighted in red.

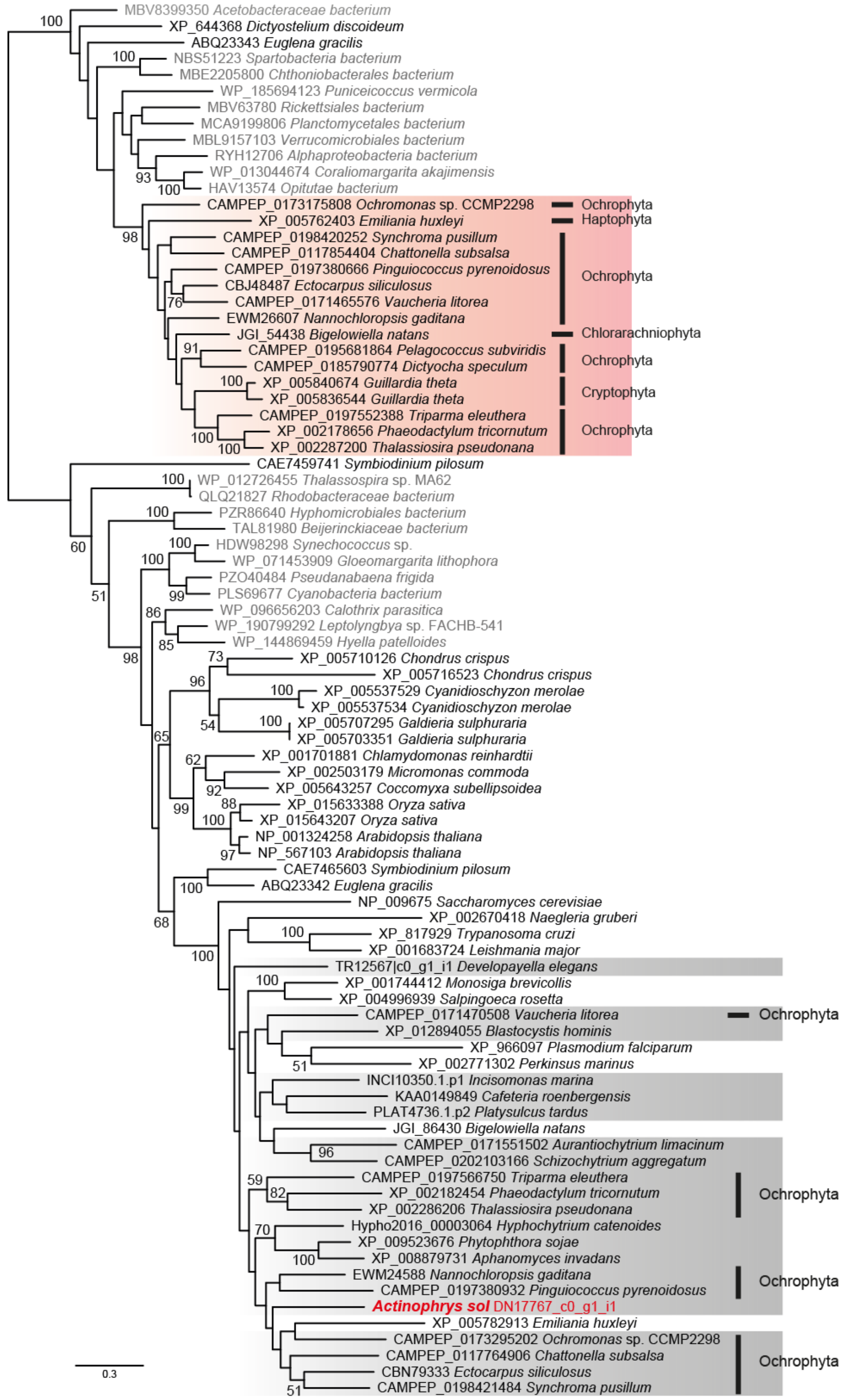

Fig. S17. Maximum likelihood tree of TKL. Maximum likelihood bootstrap values ≥ 50% are shown on each node. Light red and light grey highlights indicate the plastid-targeted and cytosolic sequences of the stramenopiles, respectively. Prokaryotic and eukaryotic taxa are in grey and black, respectively. *Actinophrys sol* is highlighted in red.

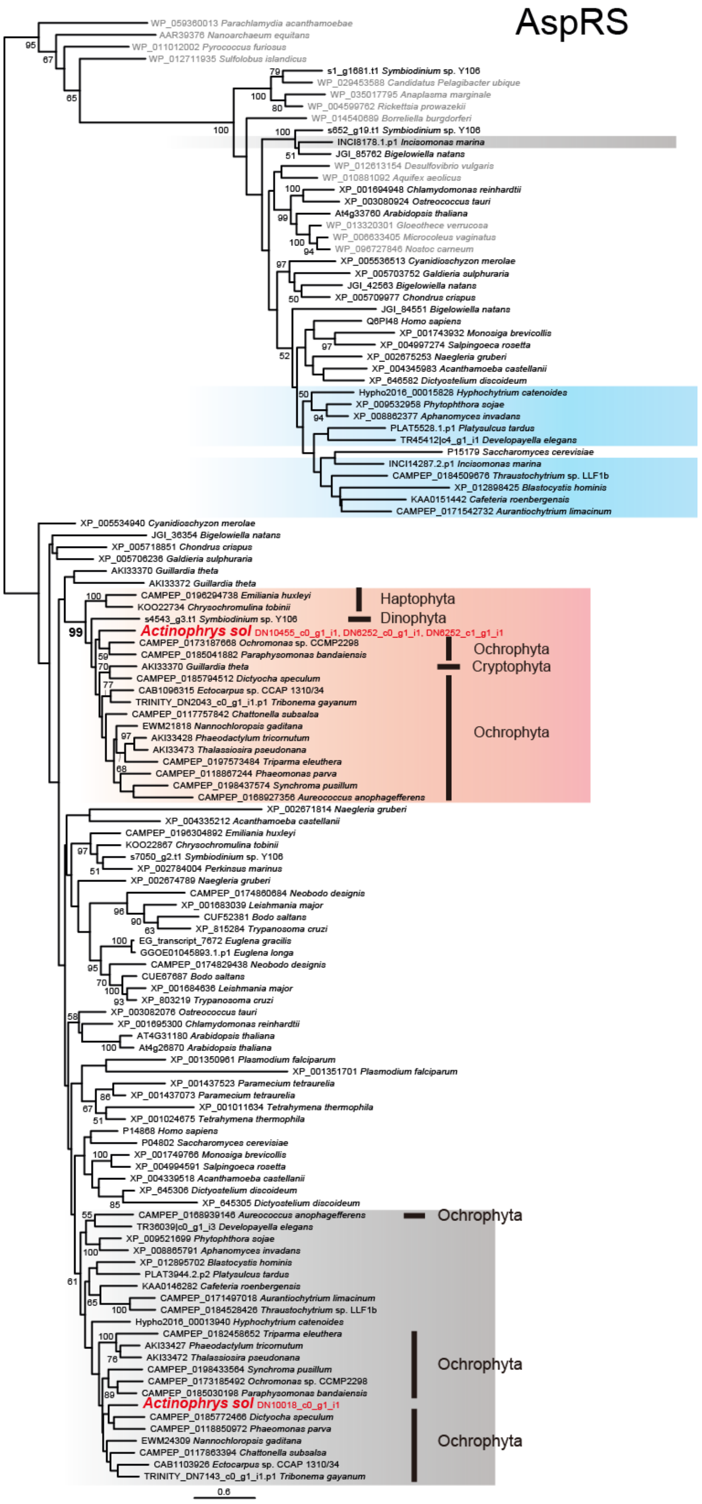

Fig. S18. Maximum likelihood tree of tRNA synthase for aspartate (AspRS). Maximum likelihood bootstrap values $\geq$ 50% are shown on each node. Light red, light blue, and light grey highlights indicate the PL-clade, mitochondrial sequences of the stramenopiles, and cytosolic sequences of the stramenopiles, respectively. Prokaryotic and eukaryotic taxa are in grey and black, respectively. *Actinophrys sol* is highlighted in red.

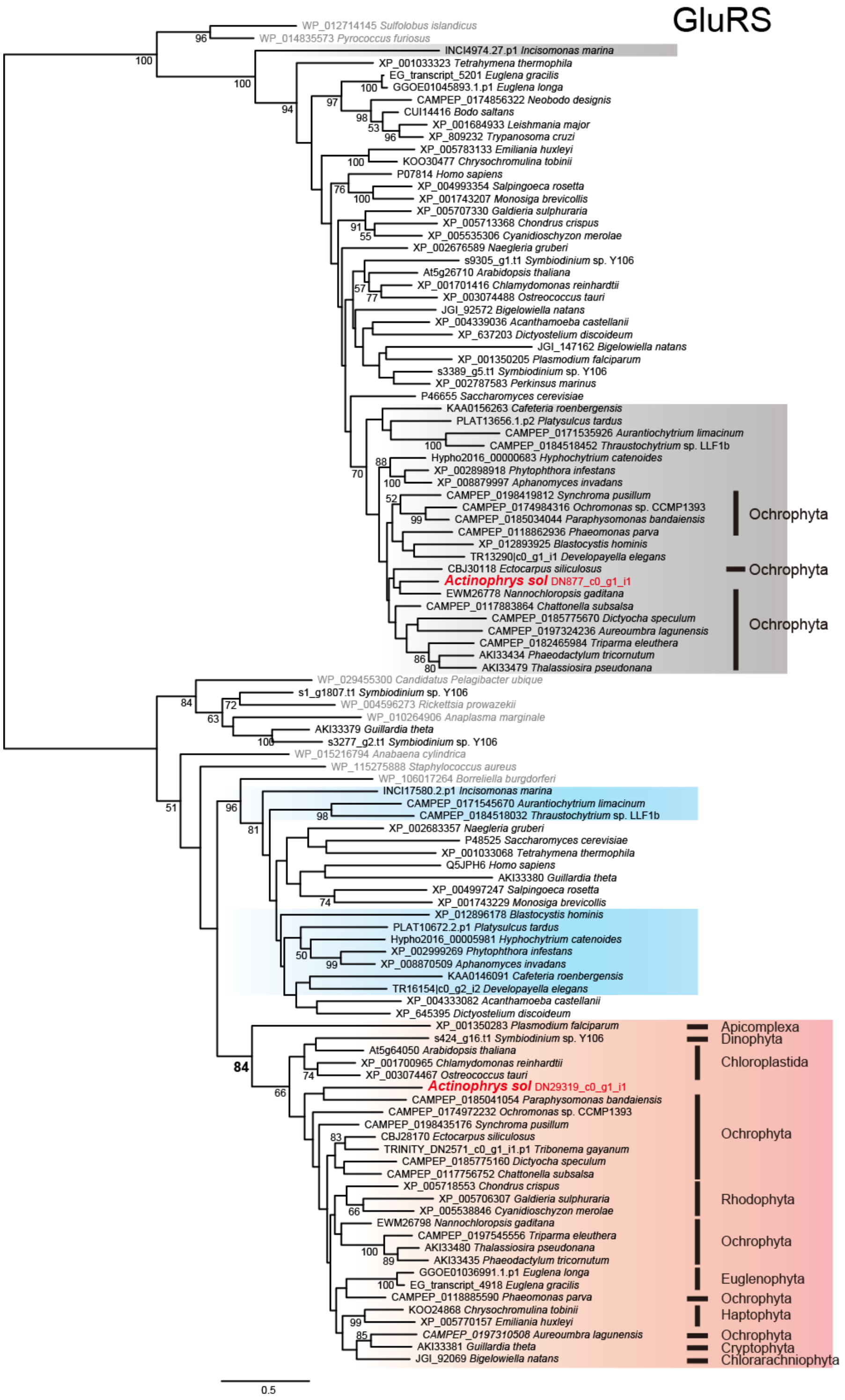

Fig. S19. Maximum likelihood tree of tRNA synthase for glutamate (GluRS). Maximum likelihood bootstrap values $\geq$ 50% are shown on each node. Light red, light blue, and light grey highlights indicate the PL-clade, mitochondrial sequences of the stramenopiles, and cytosolic sequences of the stramenopiles, respectively. Prokaryotic and eukaryotic taxa are in grey and black, respectively. *Actinophrys sol* is highlighted in red.

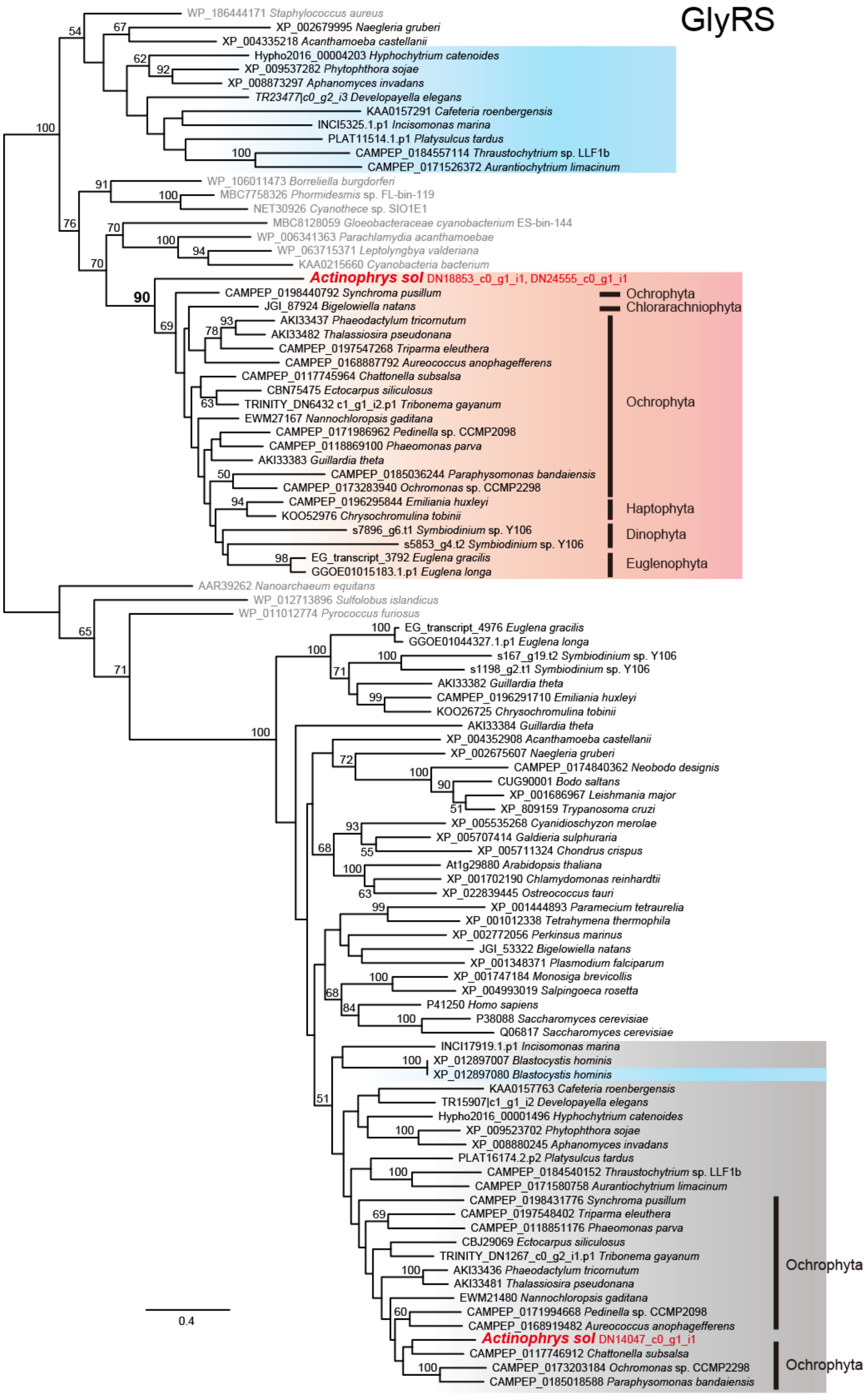

Fig. S20. Maximum likelihood tree of tRNA synthase for glycine (GlyRS). Maximum likelihood bootstrap values ≥ 50% are shown on each node. Light red, light blue, and light grey highlights indicate the PL-clade, mitochondrial sequences of the stramenopiles, and cytosolic sequences of the stramenopiles, respectively. Prokaryotic and eukaryotic taxa are in grey and black, respectively. *Actinophrys sol* is highlighted in red.

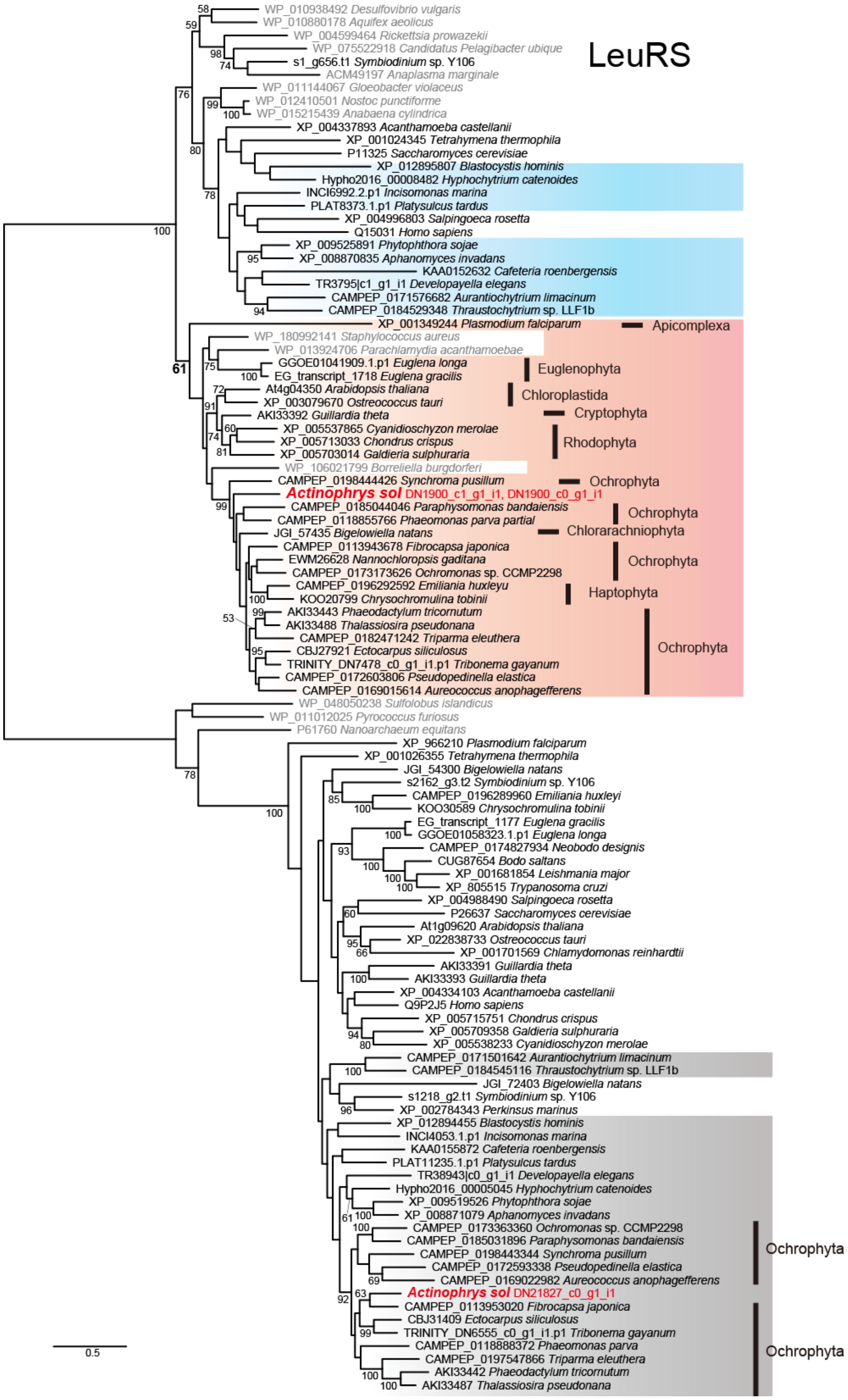

Fig. S21. Maximum likelihood tree of tRNA synthase for leucine (LeuRS). Maximum likelihood bootstrap values ≥ 50% are shown on each node. Light red, light blue, and light grey highlights indicate the PL-clade, mitochondrial sequences of the stramenopiles, and cytosolic sequences of the stramenopiles, respectively. Prokaryotic and eukaryotic taxa are in grey and black, respectively. *Actinophrys sol* is highlighted in red.

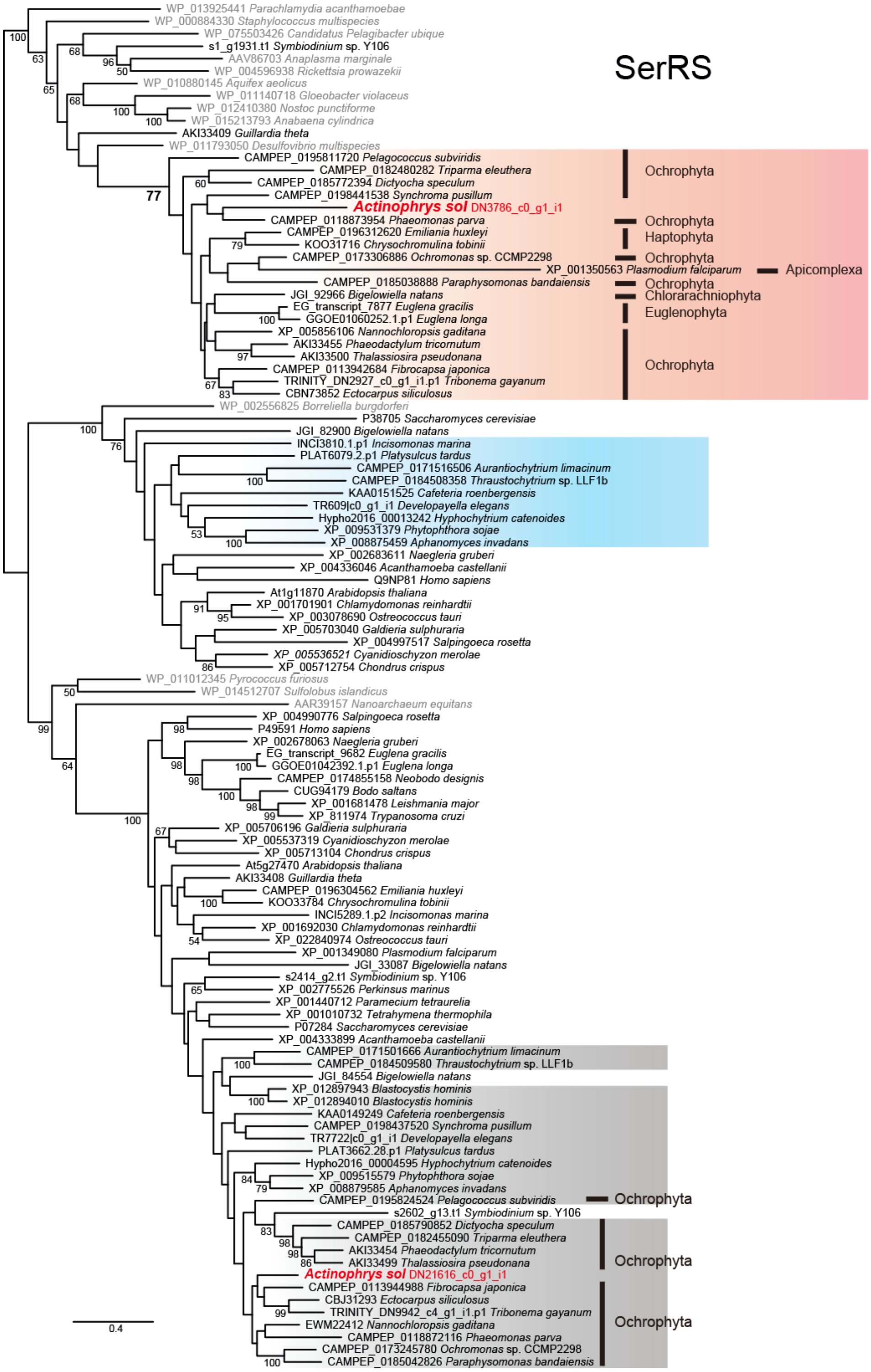

Fig. S22. Maximum likelihood tree of tRNA synthase for serine (SerRS). Maximum likelihood bootstrap values ≥ 50% are shown on each node. Light red, light blue, and light grey highlights indicate the PL-clade, mitochondrial sequences of the stramenopiles, and cytosolic sequences of the stramenopiles, respectively. Prokaryotic and eukaryotic taxa are in grey and black, respectively. *Actinophrys sol* is highlighted in red.

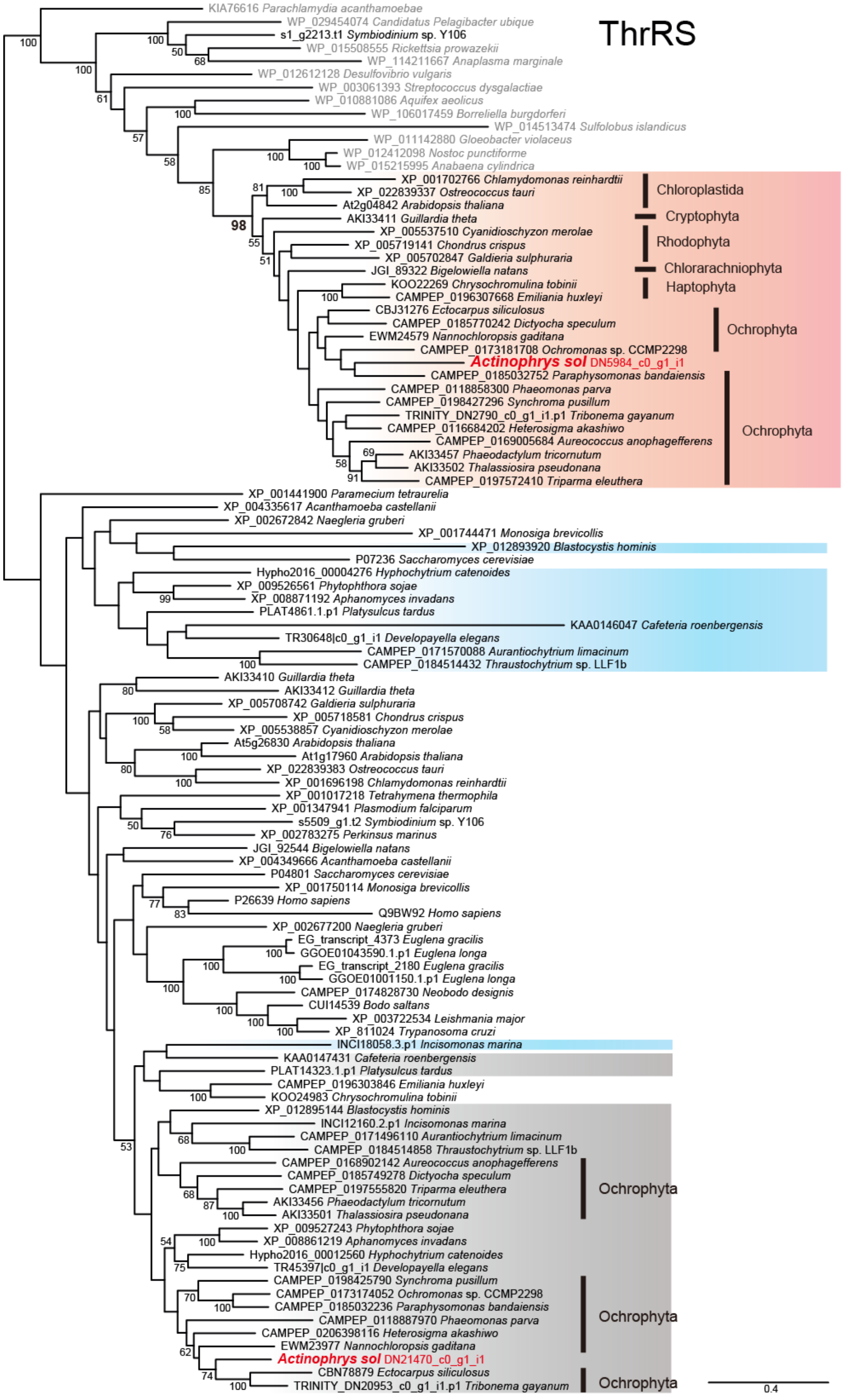

Fig. S23. Maximum likelihood tree of tRNA synthase for threonine (ThrRS). Maximum likelihood bootstrap values $\geq$ 50% are shown on each node. Light red, light blue, and light grey highlights indicate the PL-clade, mitochondrial sequences of the stramenopiles, and cytosolic sequences of the stramenopiles, respectively. Prokaryotic and eukaryotic taxa are in grey and black, respectively. *Actinophrys sol* is highlighted in red.

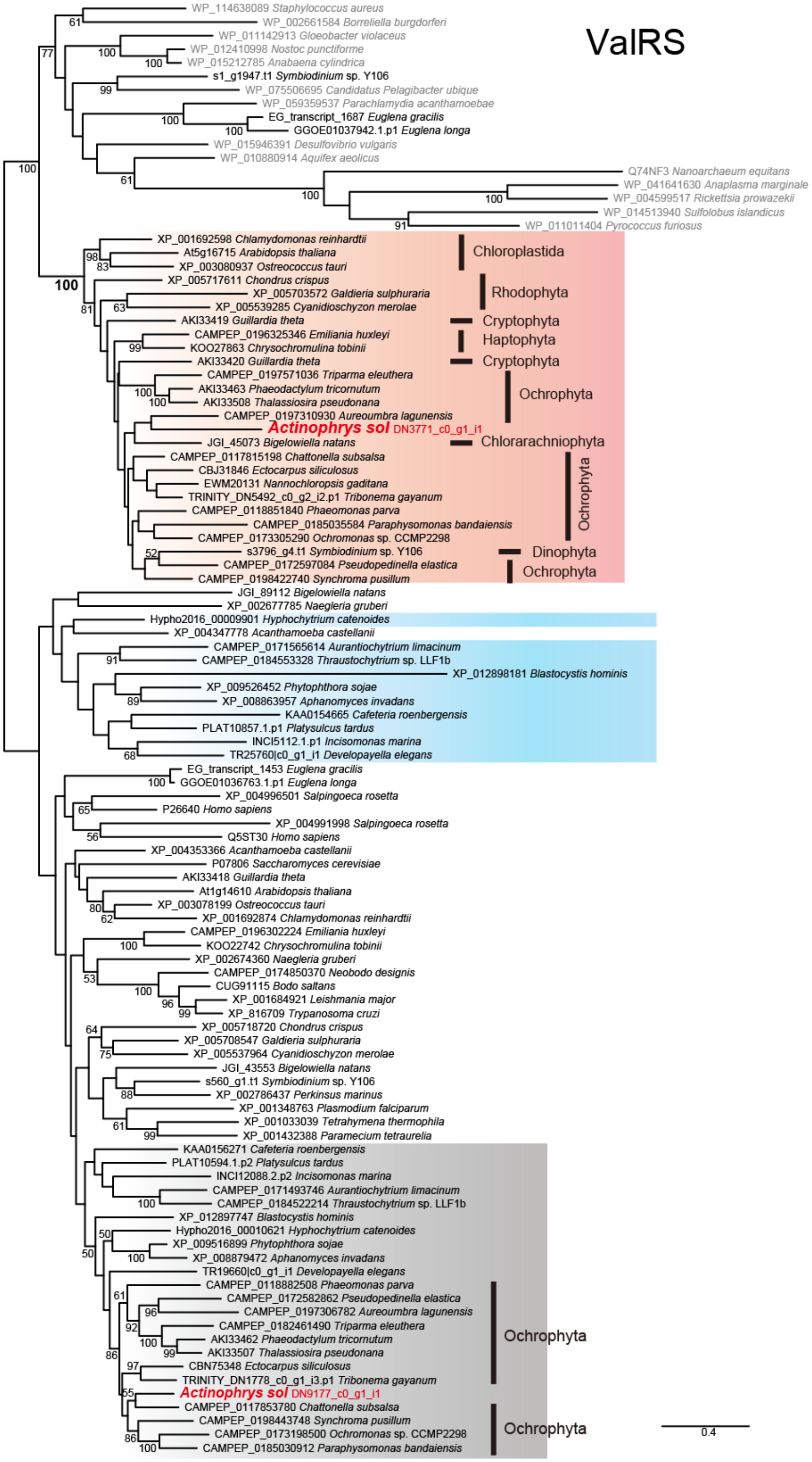

Fig. S24. Maximum likelihood tree of tRNA synthase for valine (ValRS). Maximum likelihood bootstrap values > 50% are shown on each node. Light red, light blue, and light grey highlights indicate the PL-clade, mitochondrial sequences of the stramenopiles, and cytosolic sequences of the stramenopiles, respectively. Prokaryotic and eukaryotic taxa are in grey and black, respectively. *Actinophrys sol* is highlighted in red.

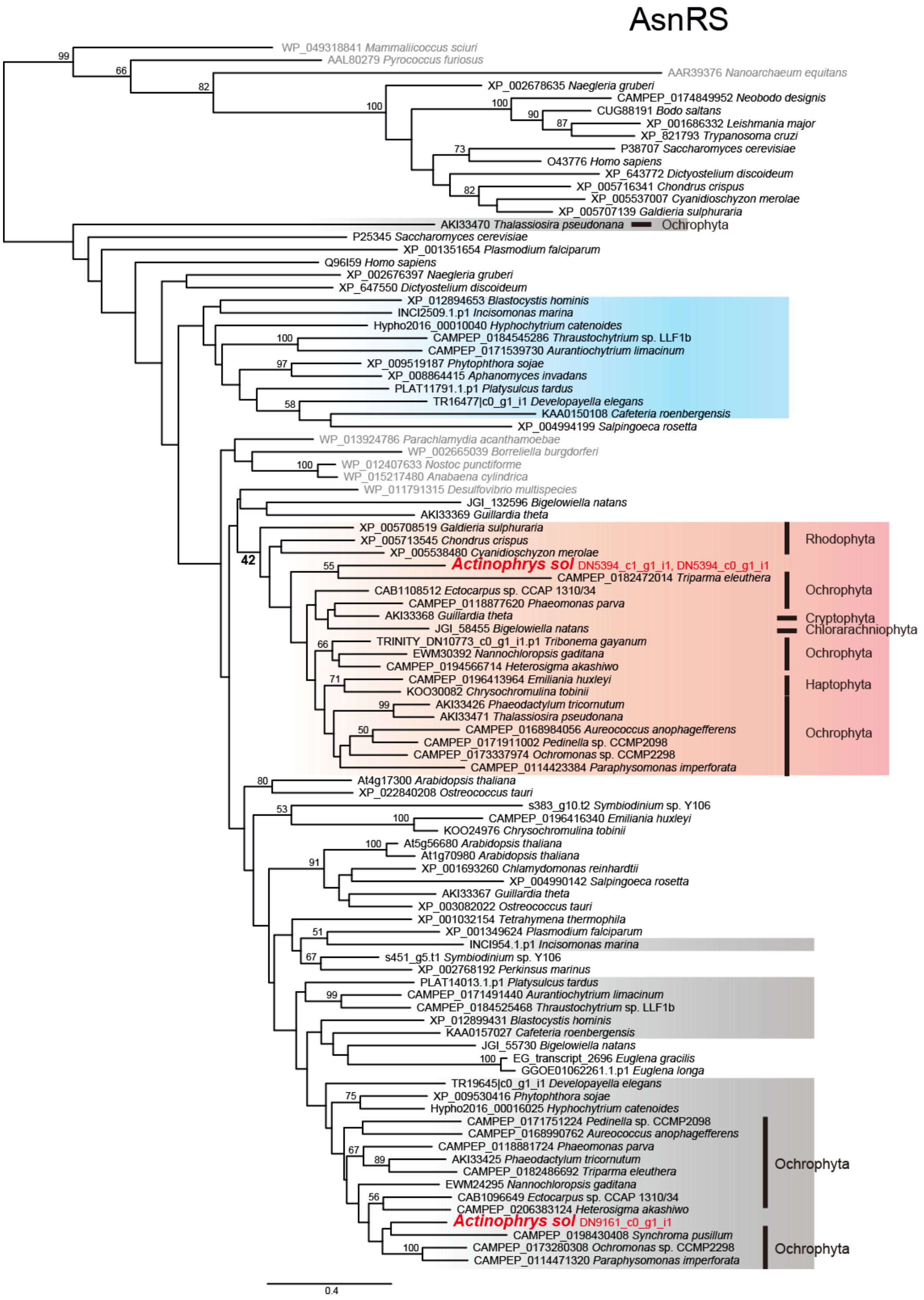

Fig. S25. Maximum likelihood tree of tRNA synthase for asparagine (AsnRS). Maximum likelihood bootstrap values $\geq$ 50% are shown on each node. Light red, light blue, and light grey highlights indicate the PL-clade, mitochondrial sequences of the stramenopiles, and cytosolic sequences of the stramenopiles, respectively. Prokaryotic and eukaryotic taxa are in grey and black, respectively. *Actinophrys sol* is highlighted in red.

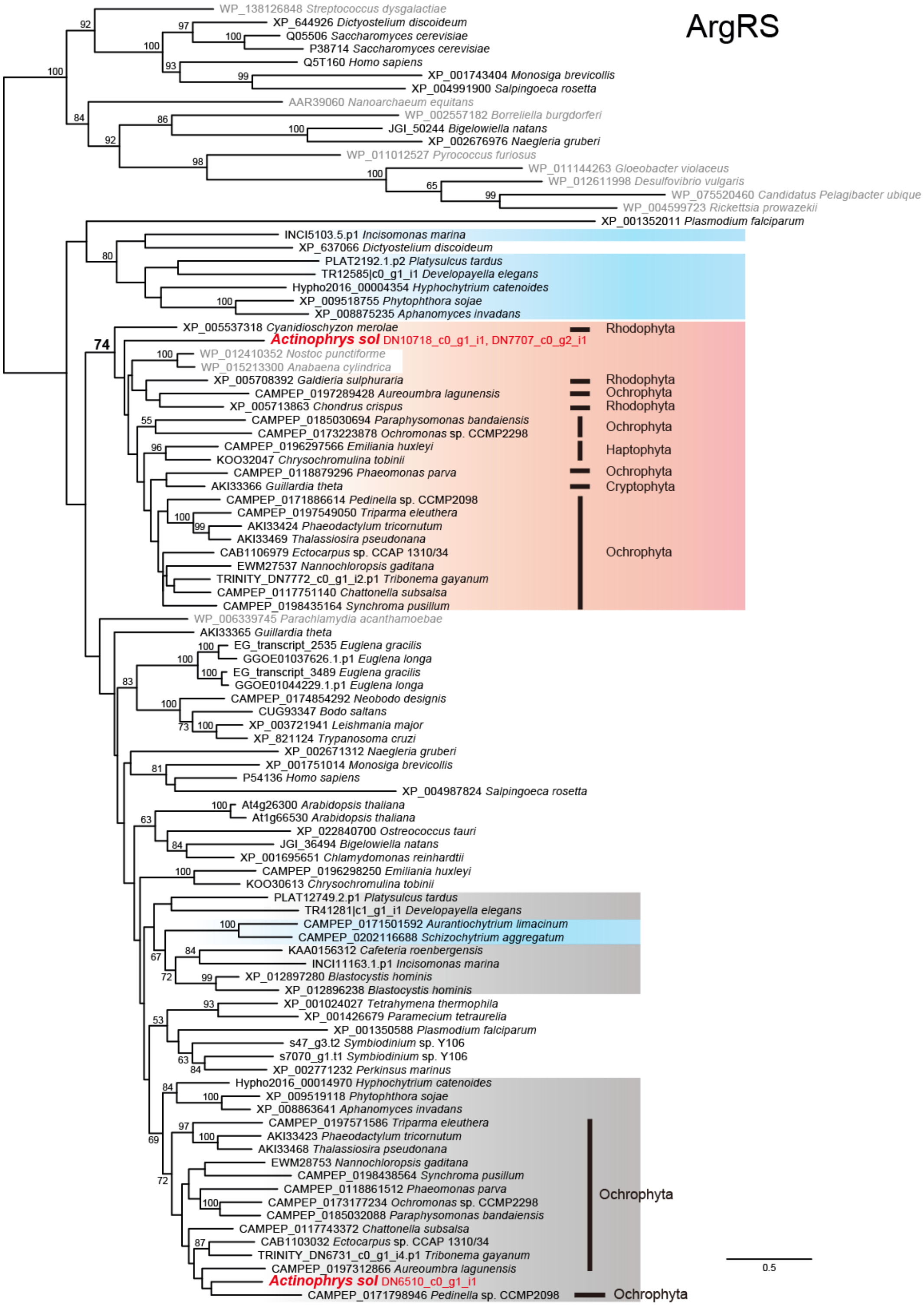

Fig. S26. Maximum likelihood tree of tRNA synthase for arginine (ArgRS). Maximum likelihood bootstrap values $\geq$ 50% are shown on each node. Light red, light blue, and light grey highlights indicate the PL-clade, mitochondrial sequences of the stramenopiles, and cytosolic sequences of the stramenopiles, respectively. Prokaryotic and eukaryotic taxa are in grey and black, respectively. *Actinophrys sol* is highlighted in red.

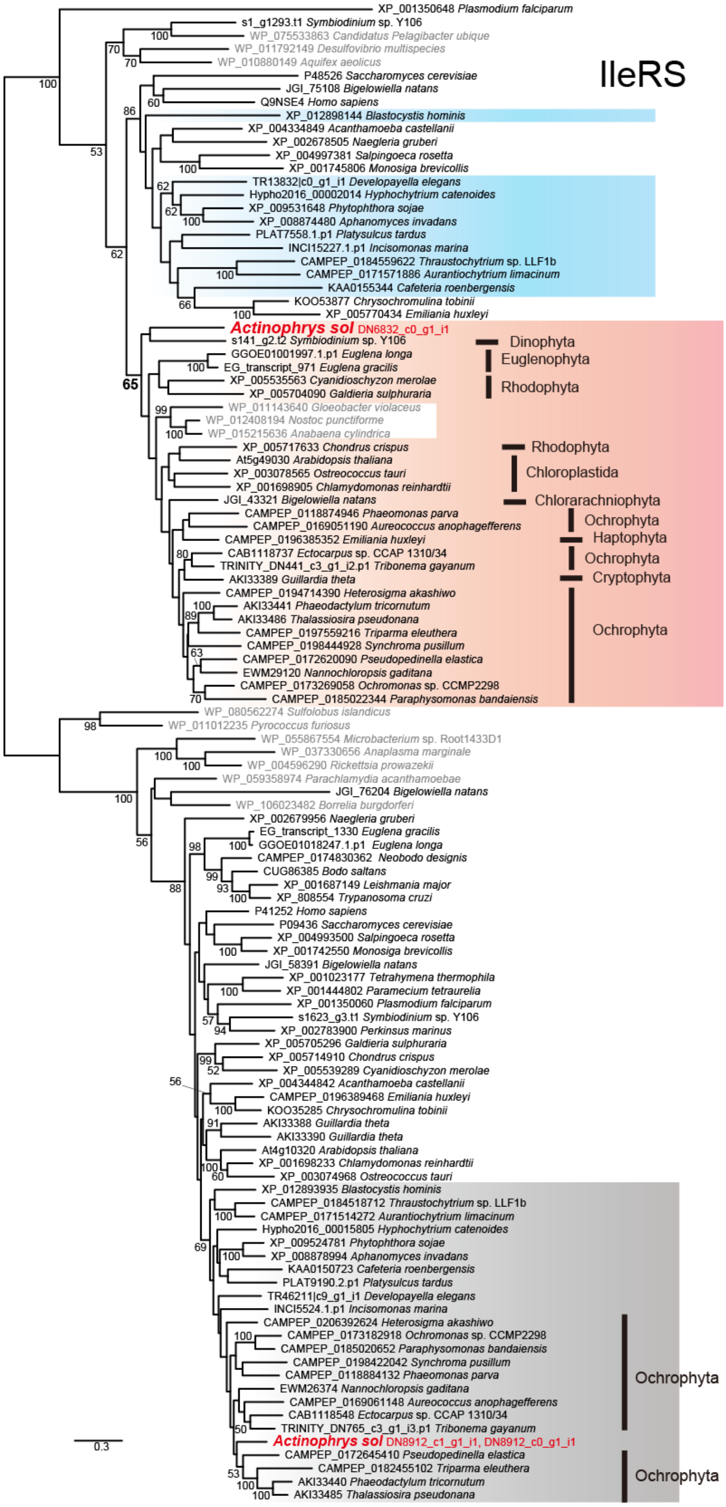

Fig. S27. Maximum likelihood tree of tRNA synthase for isoleucine (IleRS). Maximum likelihood bootstrap values $\geq$ 50% are shown on each node. Light red, light blue, and light grey highlights indicate the PL-clade, mitochondrial sequences of the stramenopiles, and cytosolic sequences of the stramenopiles, respectively. Prokaryotic and eukaryotic taxa are in grey and black, respectively. *Actinophrys sol* is highlighted in red.

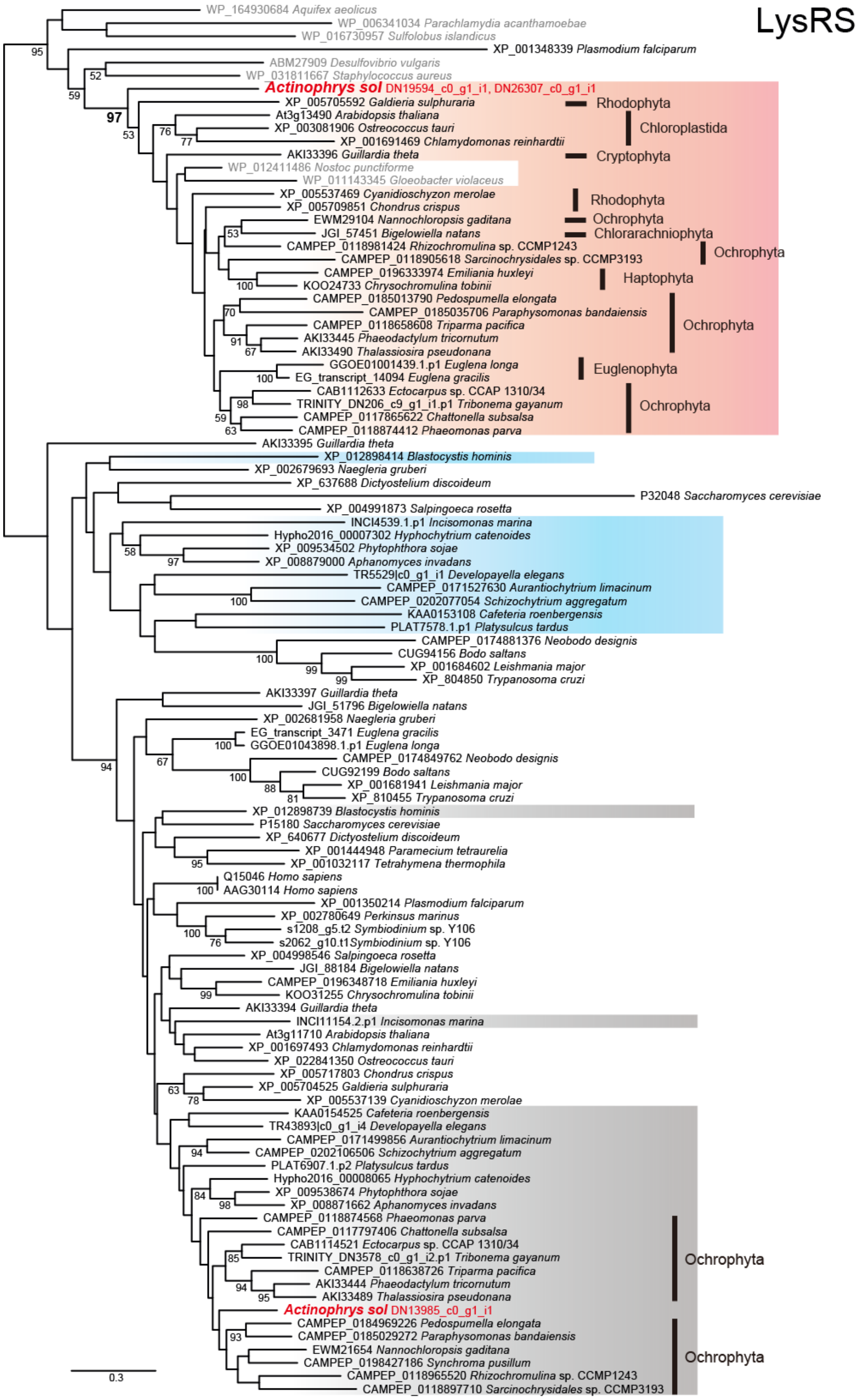

Fig. S28. Maximum likelihood tree of tRNA synthase for lysine (LysRS). Maximum likelihood bootstrap values ≥ 50% are shown on each node. Light red, light blue, and light grey highlights indicate the PL-clade, mitochondrial sequences of the stramenopiles, and cytosolic sequences of the stramenopiles, respectively. Prokaryotic and eukaryotic taxa are in grey and black, respectively. *Actinophrys sol* is highlighted in red.

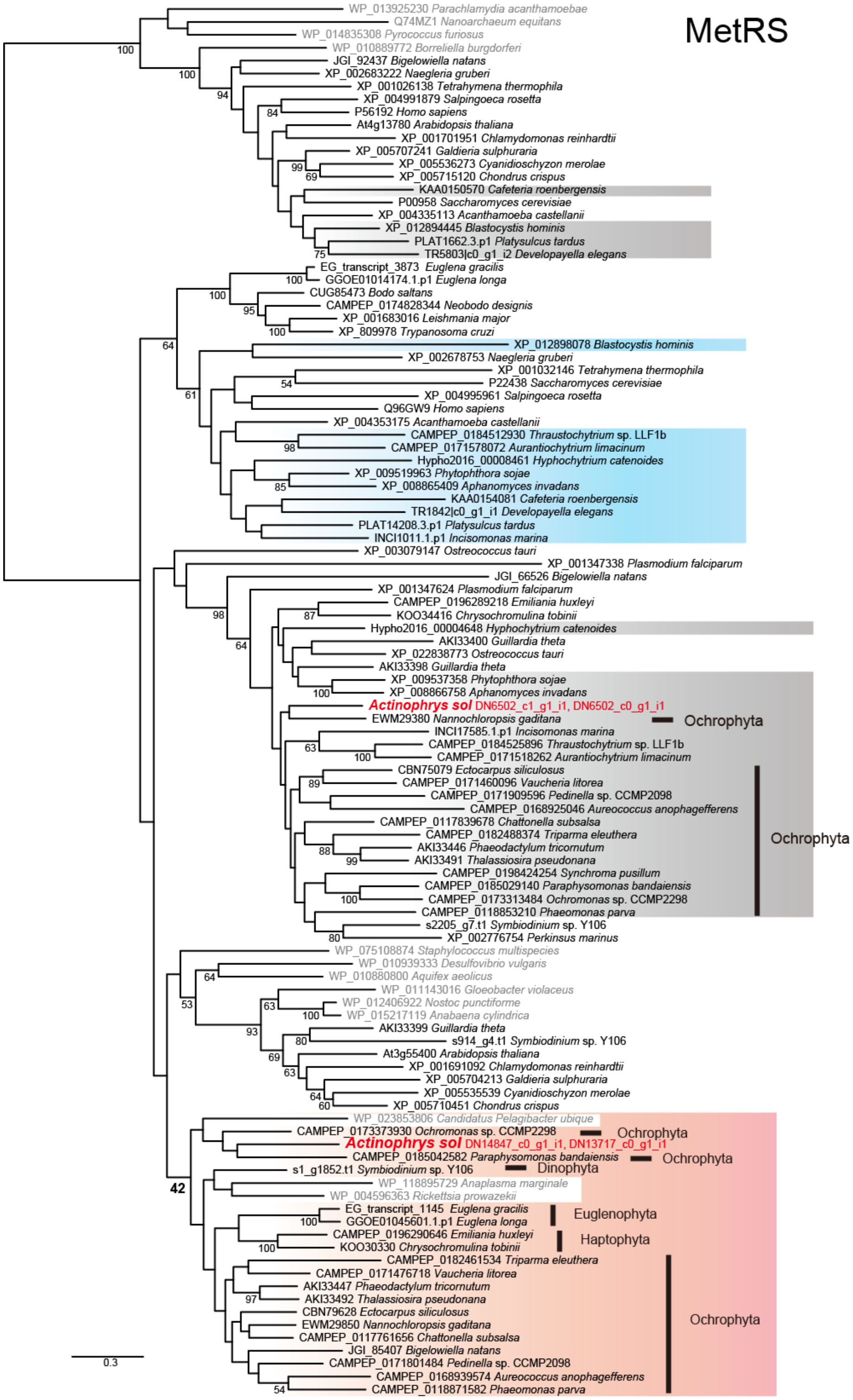

Fig. S29. Maximum likelihood tree of tRNA synthase for methionine (MetRS). Maximum likelihood bootstrap values ≥ 50% are shown on each node. Light red, light blue, and light grey highlights indicate the PL-clade, mitochondrial sequences of the stramenopiles, and cytosolic sequences of the stramenopiles, respectively. Prokaryotic and eukaryotic taxa are in grey and black, respectively. *Actinophrys sol* is highlighted in red.

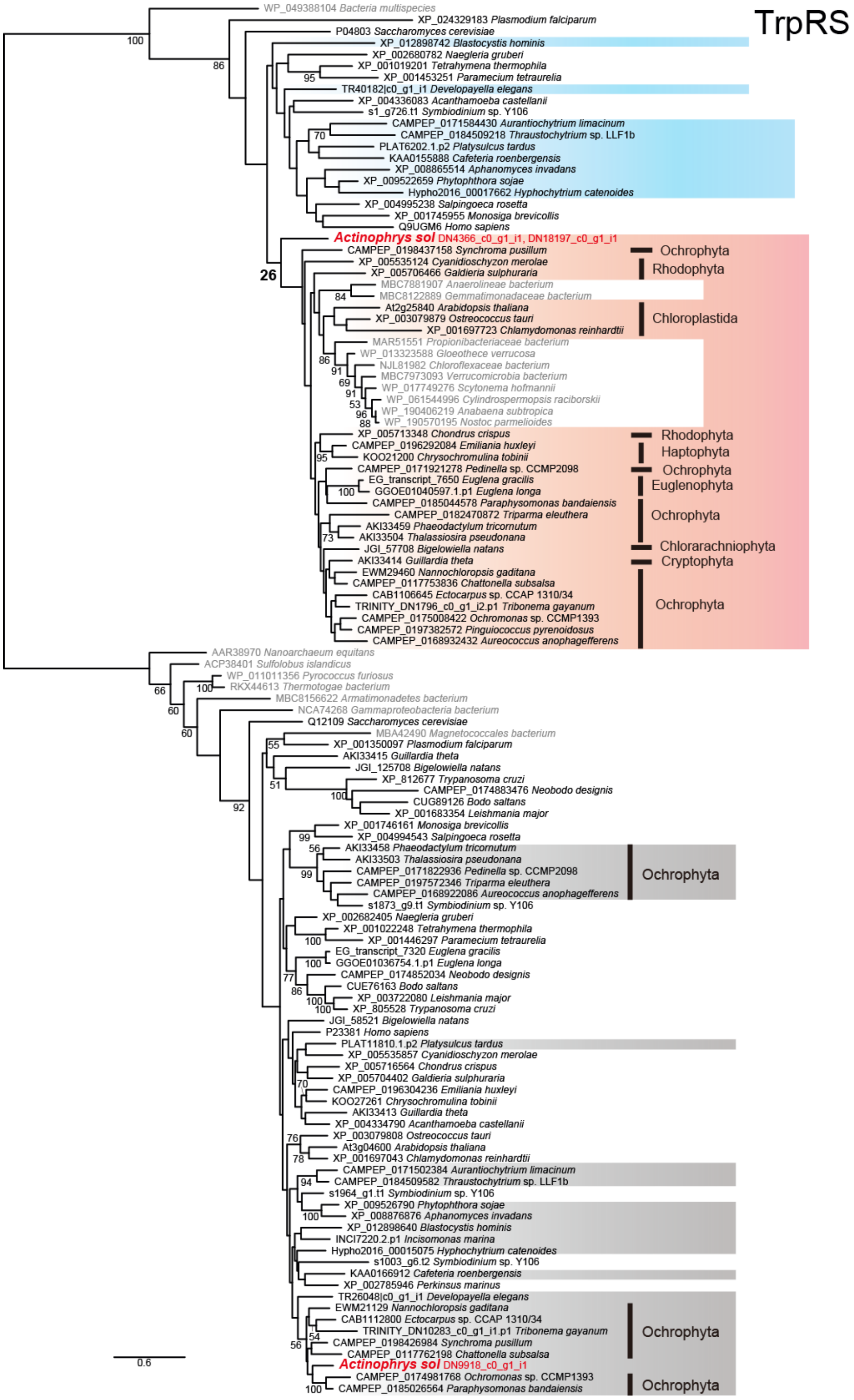

Fig. S30. Maximum likelihood tree of tRNA synthase for tryptophane (TrpRS). Maximum likelihood bootstrap values ≥ 50% are shown on each node. Light red, light blue, and light grey highlights indicate the PL-clade, mitochondrial sequences of the stramenopiles, and cytosolic sequences of the stramenopiles, respectively. Prokaryotic and eukaryotic taxa are in grey and black, respectively. *Actinophrys sol* is highlighted in red.

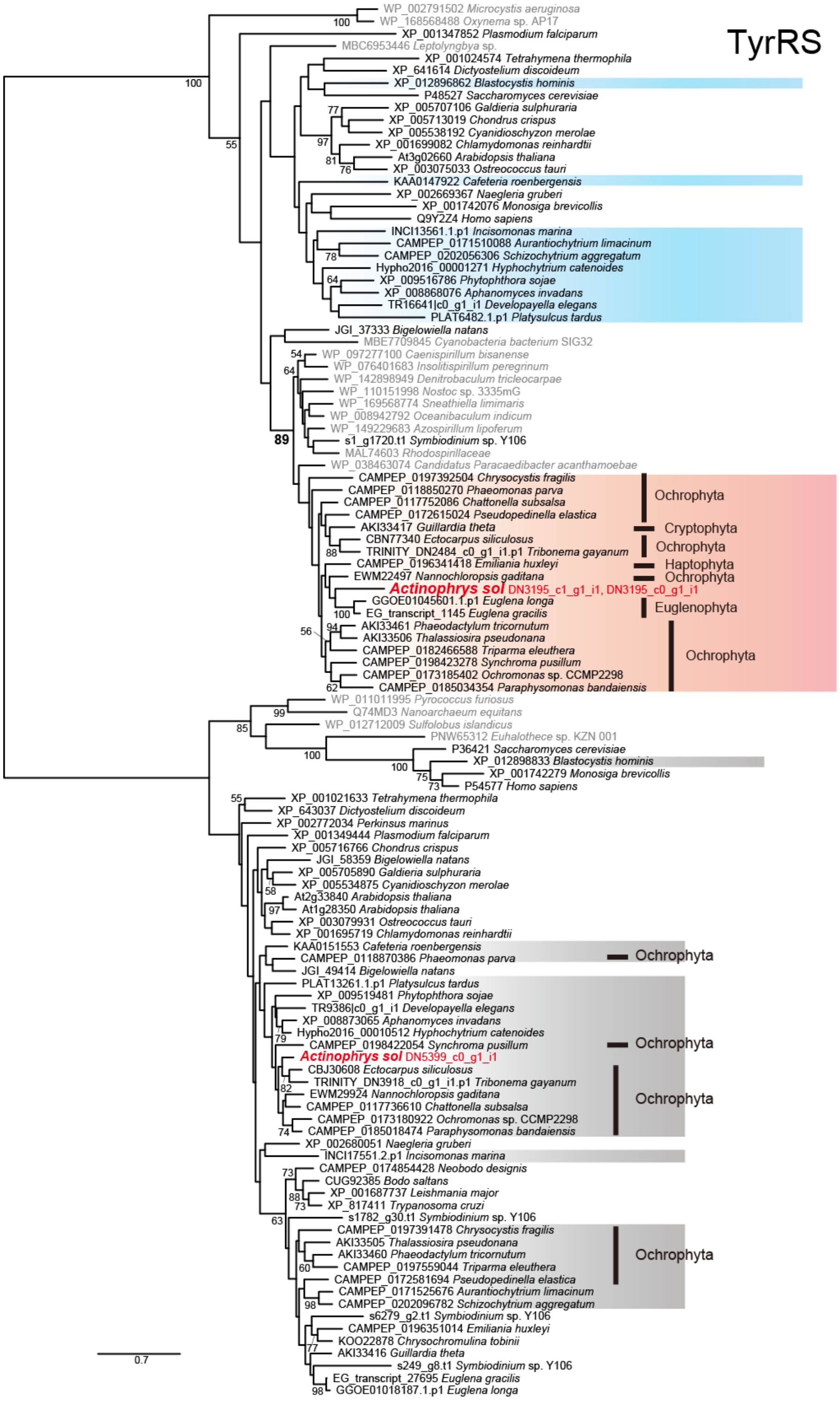

Fig. S31. Maximum likelihood tree of tRNA synthase for tyrosine (TyrRS). Maximum likelihood bootstrap values $\geq$ 50% are shown on each node. Light red, light blue, and light grey highlights indicate the PL-clade, mitochondrial sequences of the stramenopiles, and cytosolic sequences of the stramenopiles, respectively. Prokaryotic and eukaryotic taxa are in grey and black, respectively. *Actinophrys sol* is highlighted in red.

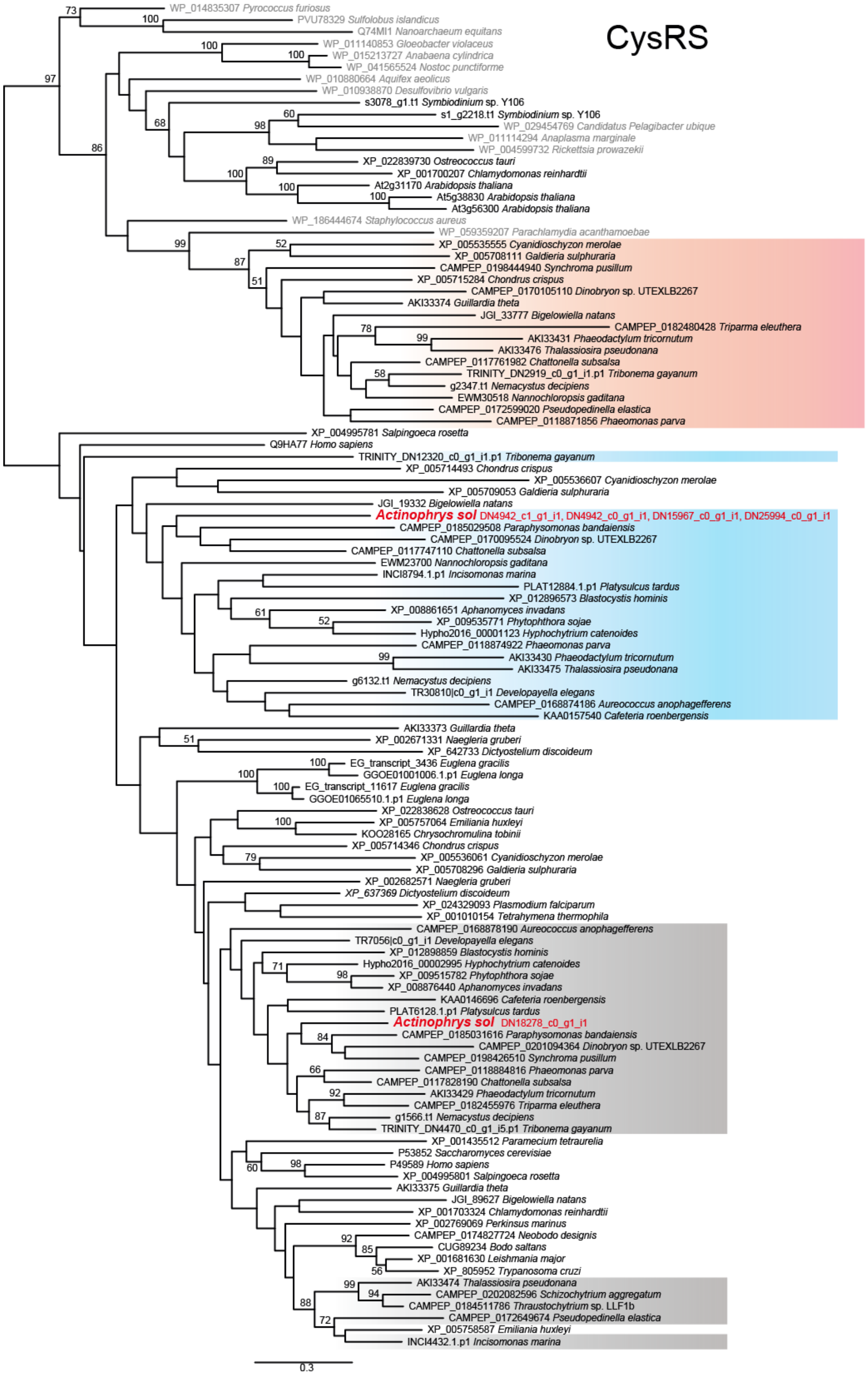

Fig. S32. Maximum likelihood tree of tRNA synthase for cysteine (CysRS). Maximum likelihood bootstrap values ≥ 50% are shown on each node. Light red, light blue, and light grey highlights indicate the PL-clade, mitochondrial sequences of the stramenopiles, and cytosolic sequences of the stramenopiles, respectively. Prokaryotic and eukaryotic taxa are in grey and black, respectively. No sequence belonging to the PL-clade but cytosolic and endogenous mitochondrial sequences were identified in *A. sol*. *Actinophrys sol* is highlighted in red.

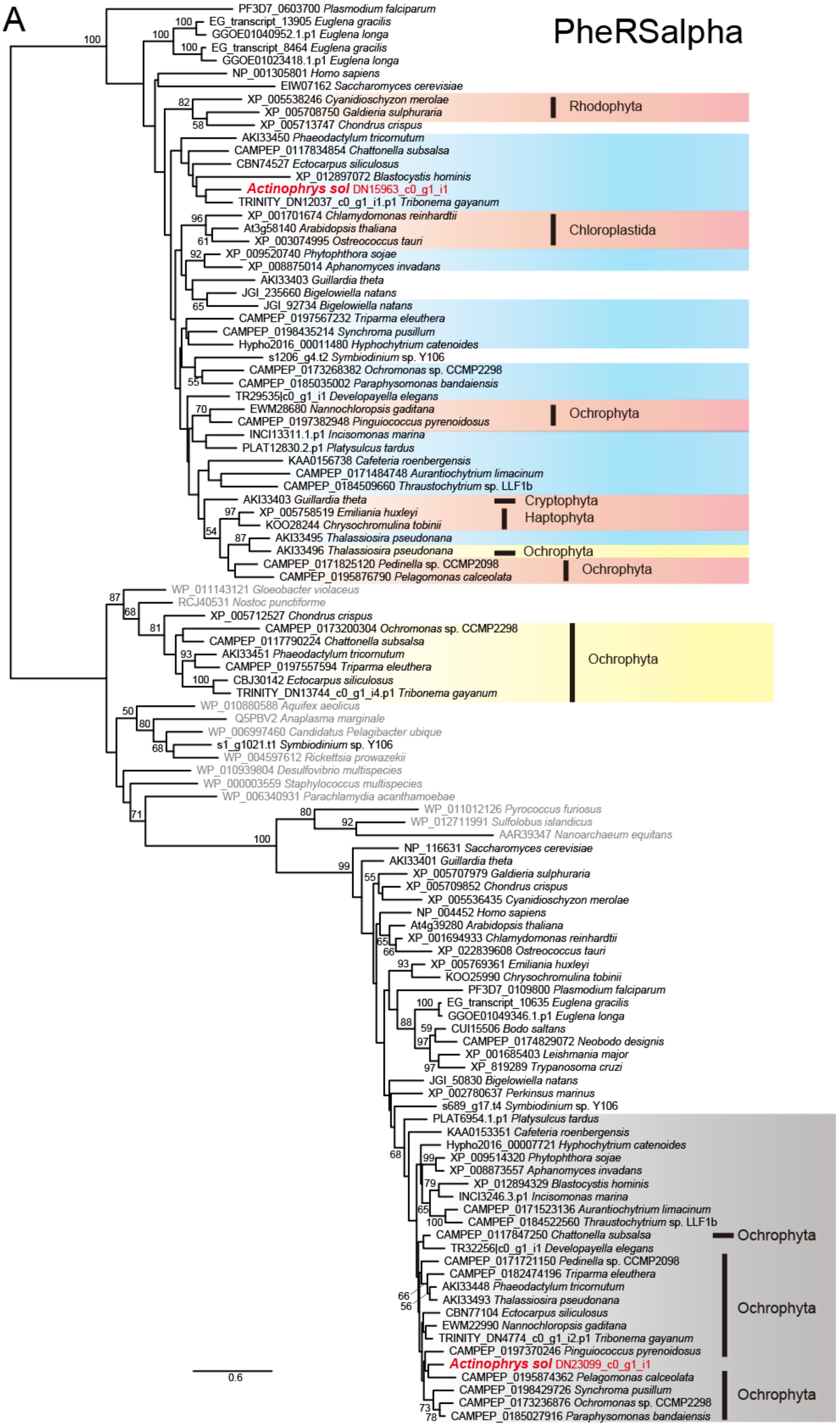
A
PheRSalpha
PF3D7_0603700 Plasmodium falciparum
EG_transcript_13905 Euglena gracilis
GGOE01040952.1.p1 Euglena longa
EG_transcript_8464 Euglena gracilis
GGOE01023418.1.p1 Euglena longa
NP_001305801 Homo sapiens
EIW07162 Saccharomyces cerevisiae
XP_005538246 Cyanidioschyzon merolae
XP_005708750 Galdieria sulphuraria
XP_005713747 Chondrus crispus
Rhodophyta
AKI33450 Phaeodactylum tricornutum
CAMPEP_0117834854 Chattonella subsalsa
CBN74527 Ectocarpus siliculosus
XP_012897072 Blastocystis hominis
Actinophrys sol DN15963_c0_g1_i1
TRINITY_DN12037_c0_g1_i1.p1 Tribonema gayanum
XP_001701674 Chlamydomonas reinhardtii
At3g58140 Arabidopsis thaliana
XP_003074995 Ostreococcus tauri
Chloroplastida
XP_009520740 Phytophthora sojae
XP_008875014 Aphanomyces invadans
AKI33403 Guillardia theta
JGI_235660 Bigelowiella natans
JGI_92734 Bigelowiella natans
CAMPEP_0197567232 Triparma eleuthera
CAMPEP_0198435214 Synchroma pusillum
Hypho2016_00011480 Hyphochytrium catenoides
s1206_g4.t2 Symbiodinium sp. Y106
CAMPEP_0173268382 Ochromonas sp. CCMP2298
CAMPEP_0185035002 Paraphysomonas bandaiensis
TR29535|c0_g1_i1 Developayella elegans
EWM28680 Nannochloropsis gaditana
CAMPEP_0197382948 Pinguiococcus pyrenoidosus
Ochrophyta
INCI13311.1.p1 Incisomonas marina
PLAT12830.2.p1 Platysulcus tardus
KAA0156738 Cafeteria roenbergensis
CAMPEP_0171484748 Aurantiochytrium limacinum
CAMPEP_0184509660 Thraustochytrium sp. LLF1b
AKI33403 Guillardia theta
Cryptophyta
XP_005758519 Emiliania huxleyi
KOO28244 Chrysochromulina tobinii
Haptophyta
AKI33495 Thalassiosira pseudonana
AKI33496 Thalassiosira pseudonana
Ochrophyta
CAMPEP_0171825120 Pedinella sp. CCMP2098
CAMPEP_0195876790 Pelagomonas calceolata
Ochrophyta
WP_011143121 Gloeobacter violaceus
RCJ40531 Nostoc punctiforme
XP_005712527 Chondrus crispus
CAMPEP_0173200304 Ochromonas sp. CCMP2298
CAMPEP_0117790224 Chattonella subsalsa
AKI33451 Phaeodactylum tricornutum
CAMPEP_0197557594 Triparma eleuthera
CBJ30142 Ectocarpus siliculosus
TRINITY_DN13744_c0_g1_i4.p1 Tribonema gayanum
Ochrophyta
WP_010880588 Aquifex aeolicus
Q5PBV2 Anaplasma marginale
WP_006997460 Candidatus Pelagibacter ubique
s1_g1021.t1 Symbiodinium sp. Y106
WP_004597612 Rickettsia prowazekii
WP_010939804 Desulfovibrio multispecies
WP_000003559 Staphylococcus multispecies
WP_006340931 Parachlamydia acanthamoebae
WP_011012126 Pyrococcus furiosus
WP_012711991 Sulfolobus islandicus
AAR39347 Nanoarchaeum equitans
NP_116631 Saccharomyces cerevisiae
AKI33401 Guillardia theta
XP_005707979 Galdieria sulphuraria
XP_005709852 Chondrus crispus
XP_005536435 Cyanidioschyzon merolae
NP_004452 Homo sapiens
At4g39280 Arabidopsis thaliana
XP_001694933 Chlamydomonas reinhardtii
XP_022839608 Ostreococcus tauri
XP_005769361 Emiliania huxleyi
KOO25990 Chrysochromulina tobinii
PF3D7_0109800 Plasmodium falciparum
EG_transcript_10635 Euglena gracilis
GGOE01049346.1.p1 Euglena longa
CUI15506 Bodo saltans
CAMPEP_0174829072 Neobodo designis
XP_001685403 Leishmania major
XP_819289 Trypanosoma cruzi
JGI_50830 Bigelowiella natans
XP_002780637 Perkinsus marinus
s689_g17.t4 Symbiodinium sp. Y106
PLAT6954.1.p1 Platysulcus tardus
KAA0153351 Cafeteria roenbergensis
Hypho2016_00007721 Hyphochytrium catenoides
XP_009514320 Phytophthora sojae
XP_008873557 Aphanomyces invadans
XP_012894329 Blastocystis hominis
INCI3246.3.p1 Incisomonas marina
CAMPEP_0171523136 Aurantiochytrium limacinum
CAMPEP_0184522560 Thraustochytrium sp. LLF1b
CAMPEP_0117847250 Chattonella subsalsa
Ochrophyta
TR32256|c0_g1_i1 Developayella elegans
CAMPEP_0171721150 Pedinella sp. CCMP2098
CAMPEP_0182474196 Triparma eleuthera
AKI33448 Phaeodactylum tricornutum
AKI33493 Thalassiosira pseudonana
CBN77104 Ectocarpus siliculosus
EWM22990 Nannochloropsis gaditana
TRINITY_DN4774_c0_g1_i2.p1 Tribonema gayanum
CAMPEP_0197370246 Pinguiococcus pyrenoidosus
Ochrophyta
Actinophrys sol DN23099_c0_g1_i1
CAMPEP_0195874362 Pelagomonas calceolata
CAMPEP_0198429726 Synchroma pusillum
CAMPEP_0173236876 Ochromonas sp. CCMP2298
CAMPEP_0185027916 Paraphysomonas bandaiensis
Ochrophyta
0.6

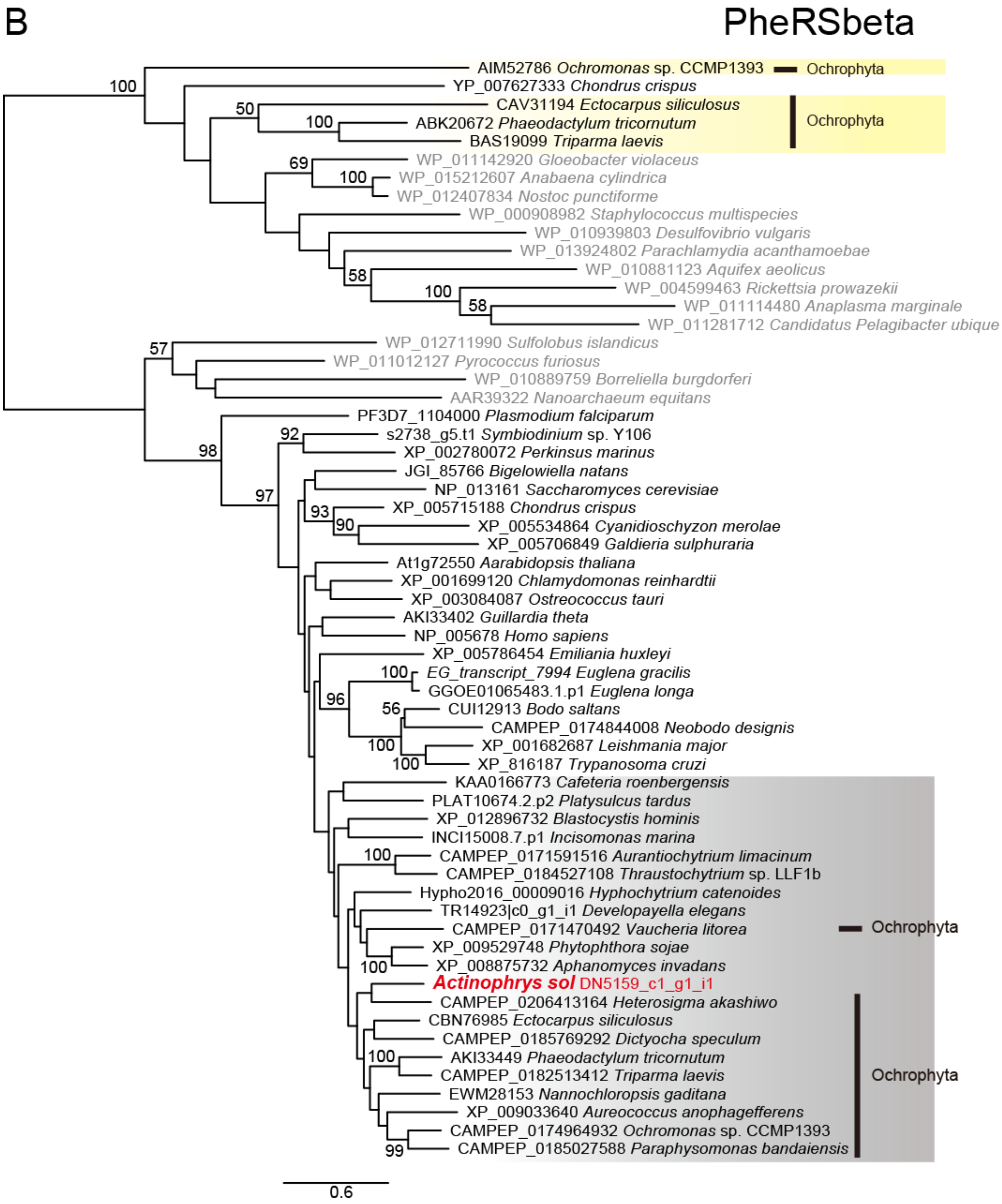

Fig. S33. Maximum likelihood tree of tRNA synthase for phenylalanine (PheRS). A. alpha subunit, B. beta subunit. Maximum likelihood bootstrap values $\geq$ 50% are shown on each node. Light red, light yellow, light blue, and light grey highlights indicate dual-targeted sequences, plastid sequences of the stramenopiles, mitochondrial sequences of the stramenopiles, and cytosolic sequences of the stramenopiles, respectively. Dual-targeted, plastid targeted, and mitochondrial targeted sequences form the monophyletic clade in alpha subunit and, in beta subunit, there is no mitochondrial targeted sequences as reported in Gile et al. (2015). Prokaryotic and eukaryotic taxa are in grey and black, respectively. *Actinophrys sol* is highlighted in red.

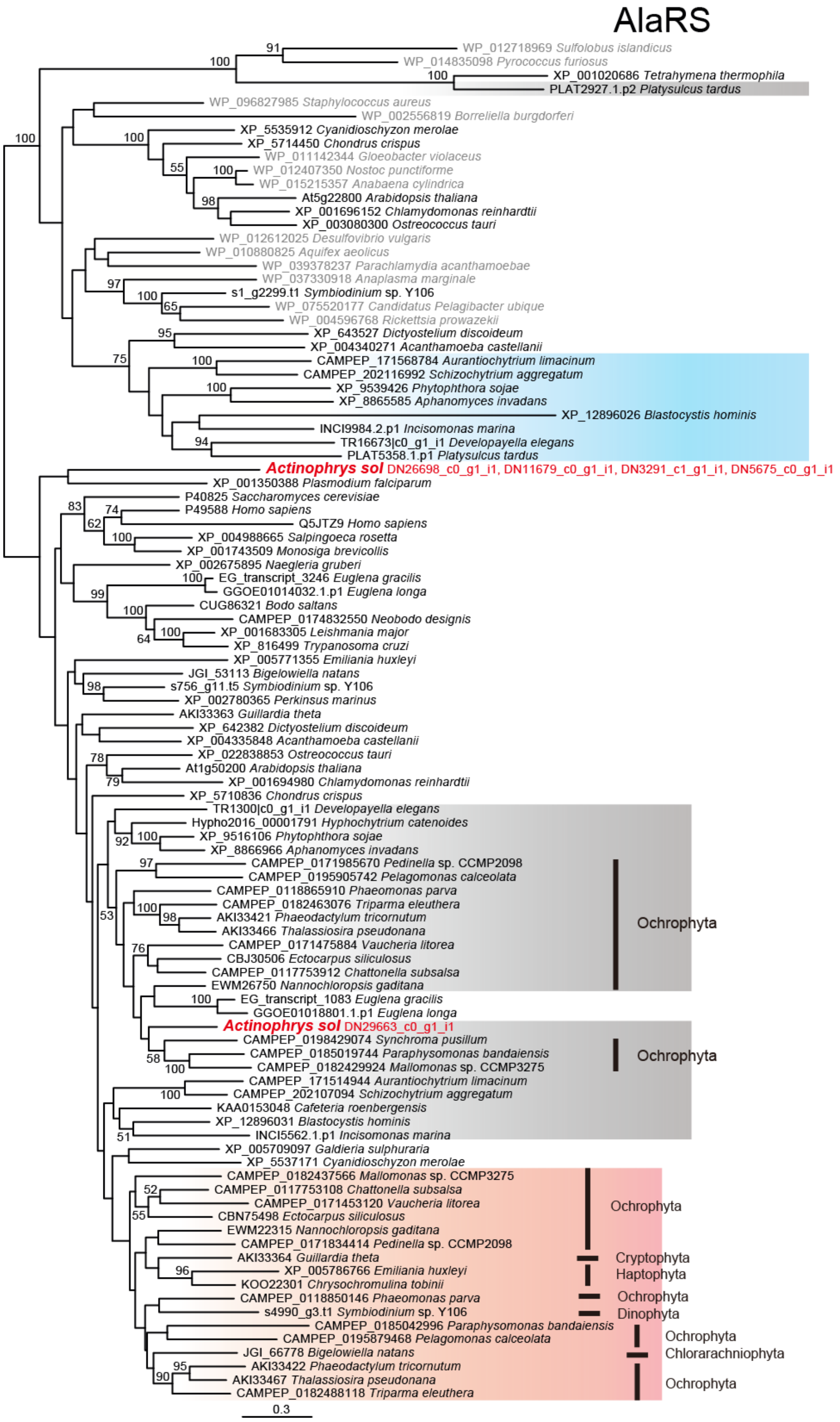

Fig. S34. Maximum likelihood tree of tRNA synthase for alanine (AlaRS). Maximum likelihood bootstrap values ≥ 50% are shown on each node. Light red, light blue, and light grey highlights indicate the PL-clade, mitochondrial sequences of the stramenopiles, and cytosolic sequences of the stramenopiles, respectively. Prokaryotic and eukaryotic taxa are in grey and black, respectively. *Actinophrys sol* is highlighted in red.

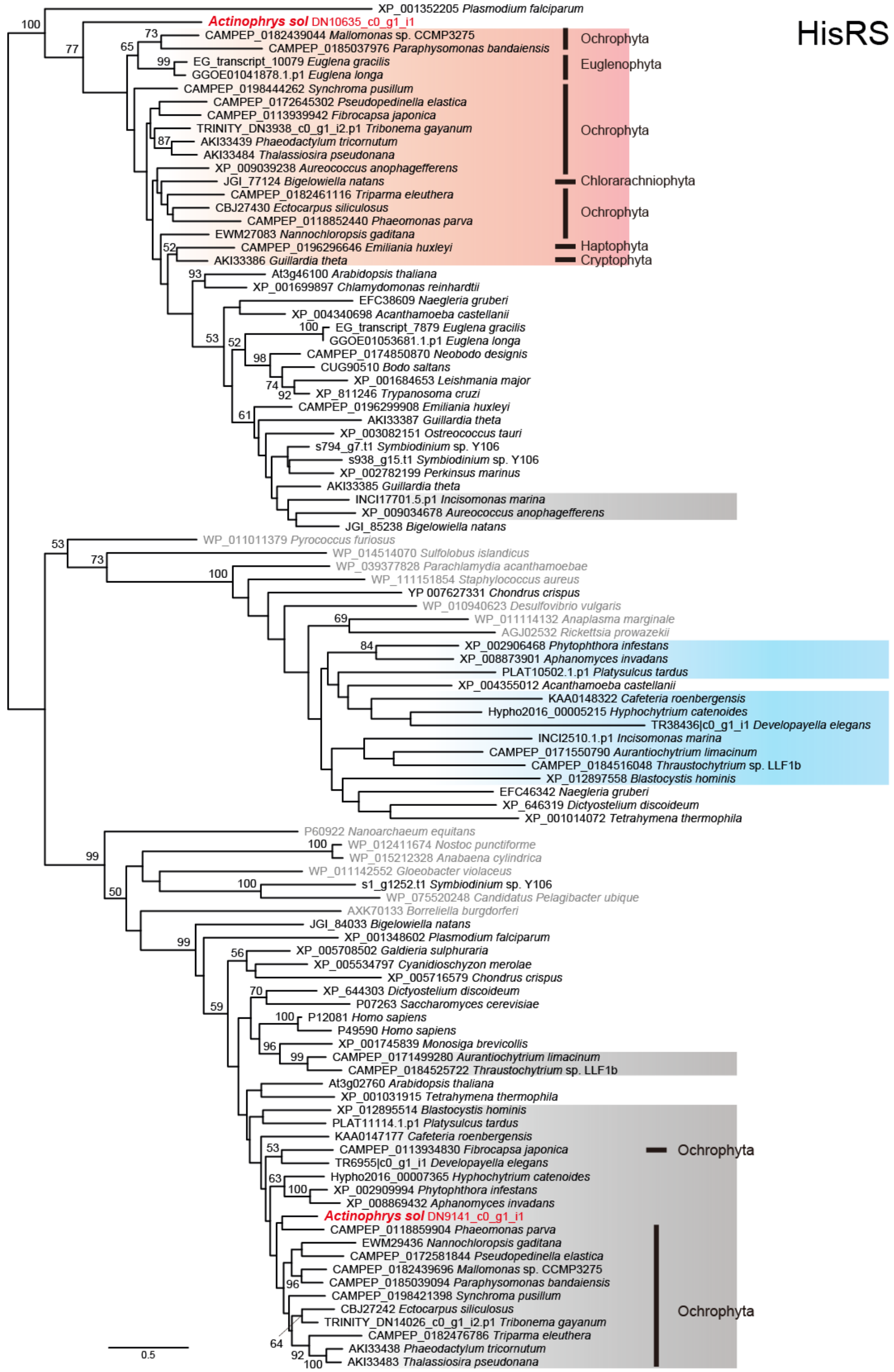

Fig. S35. Maximum likelihood tree of tRNA synthase for histidine (HisRS). Maximum likelihood bootstrap values $\geq$ 50% are shown on each node. Light red, light blue, and light grey highlights indicate the PL-clade, mitochondrial sequences of the stramenopiles, and cytosolic sequences of the stramenopiles, respectively. Prokaryotic and eukaryotic taxa are in grey and black, respectively. *Actinophrys sol* is highlighted in red.

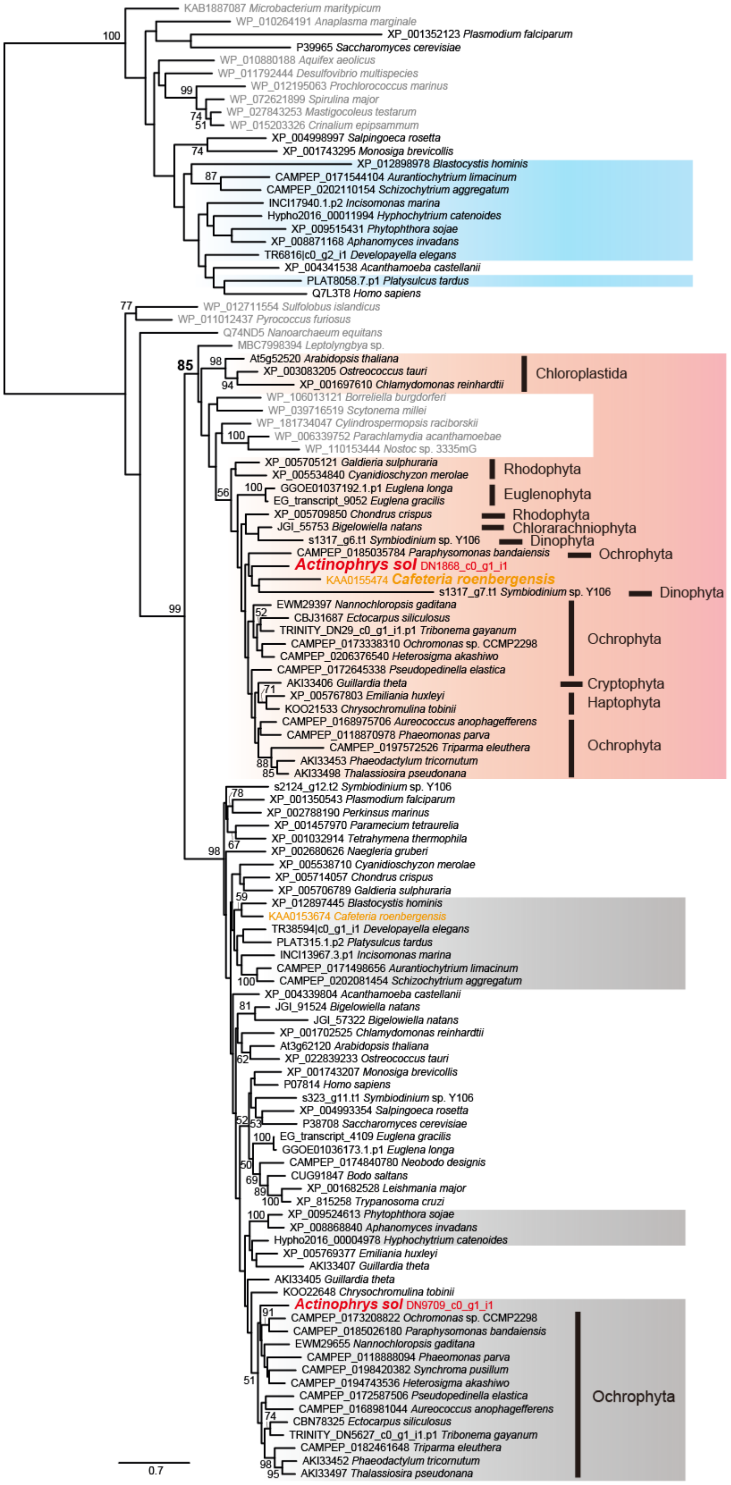

Fig. S36. Maximum likelihood tree of tRNA synthase for proline (ProRS). Maximum likelihood bootstrap values ≥ 50% are shown on each node. Light red, light blue, and light grey highlights indicate the PL-clade, mitochondrial sequences of the stramenopiles, and cytosolic sequences of the stramenopiles, respectively. Prokaryotic and eukaryotic taxa are in grey and black, respectively. *Actinophrys sol* and *Cafeteria roenbergensis* are highlighted in red and orange, respectively.

Supplementary references

Altschul SF, et al. 1997. Gapped BLAST and PSI-BLAST: a new generation of protein database search programs. *Nucleic Acids Res.* 25:3389-3402.

Bankevich A, et al. 2012. SPAdes: a new genome assembly algorithm and its applications to single-cell sequencing. *J. Comput. Biol*. 19:455-477.

Bannai H, Tamada Y, Maruyama O, Nakai K, Miyano S. 2002. Extensive feature detection of N-terminal protein sorting signal. *Bioinformatics* 18:298-305.

Bendtsen JD, Nielsen H, von Heijne G, Brunak S. 2004. Improved prediction of signal peptides: SignalP 3.0. *J. Mol. Biol.* 340:783-795.

Burger G, Gray MW, Forget L, Lang BF. 2013. Strikingly bacteria-like and gene-rich mitochondrial genomes throughout jakobid protists. *Genome Biol. Evol.* 5:418–438.

Claros MG, Vincens P. 1996. Computational method to predict mitochondrially imported proteins and their targeting sequences. *Eur. J. Biochem*. 241:779-786.

Dorrell RG, et al. 2019. Principles of plastid reductive evolution illuminated by nonphotosynthetic chrysophytes. *Proc. Natl. Acad. Sci. U.S.A.* 116:6914-6923.

Emanuelsson O, Nielsen H, Brunak S, von Heijne G. 2000. Predicting subcellular localization of proteins based on their N-terminal amino acid sequence. *J. Mol. Biol.* 300:1005-1016.

Fukasawa Y, Tsuji J, Fu SC, Tomii K, Horton P, Imai K. 2015. MitoFates: improved prediction of mitochondrial targeting sequences and their cleavage sites. *Mol. Cell. Proteomics* 14:1113-1126.

Gile GH, Moog D, Slamovits CH, Maier UG, Archibald JM. 2015. Dual organellar targeting of aminoacyl-tRNA synthetases in diatoms and cryptophytes. *Genome Biol. Evol.* 7:1728–1742.

Gruber A, Rocap G, Kroth PG, Armbrust EV, Mock T. 2015. Plastid proteome prediction for diatoms and other algae with secondary plastids of the red lineage. *Plant J.* **81**, 519-528.

Haas BJ, et al. 2013. De novo transcript sequence reconstruction from RNA-seq using the Trinity platform for reference generation and analysis. *Nat. Protoc*. 8:1494–1512.

Hall TA. 1999. BioEdit: a user-friendly biological sequence alignment editor and analysis program for windows 95/98/NT. *Nucleic Acids Symp. Ser.* 41:95–98.

Kamikawa R, et al. 2015. Proposal of a twin arginine translocator system-mediated constraint against loss of ATP synthase complex in nonphotosynthetic plastid genomes. *Mol. Biol. Evol.* 32:2598-2604.

Kanehisa M, Goto S, Sato Y, Furumichi M, Tanabe M. 2012. KEGG for integration and interpretation of large-scale molecular data sets. *Nucleic Acids. Res*. 40(database issue):D109–D114.

Katoh K, Standley DM. 2013. MAFFT multiple sequence alignment software version 7: improvements in performance and usability. *Mol. Biol. Evol.* 30:772–780.

Kayama M, et al. 2020. Highly reduced plastid genomes of the non-photosynthetic dictyochophyceans *Pteridomonas* spp. (Ochrophyta, SAR) are retained for tRNA-Glu-based organellar heme biosynthesis. *Front. Plant Sci.* 11:602455

Keeling PJ, et al. 2014. The Marine Microbial Eukaryote Transcriptome Sequencing Project (MMETSP): illuminating the functional diversity of eukaryotic life in the oceans through transcriptome sequencing. *PLoS Biol.* 12:e1001889.

Miyahara M, Aoi M, Inoue-Kashino N, Kashino Y, Ifuku K. 2013. Highly efficient transformation of the diatom *Phaeodactylum tricornutum* by multi-pulse electroporation. *Biosci. Biotechnol. Biochem*. 77: 874-876.

Moriya Y, Itoh M, Okuda S, Yoshizawa AC, Kanehisa M. 2007. KAAS: An automatic genome annotation and pathway reconstruction server. *Nucleic Acids. Res.* 35(Web server issue):W182-W185.

Nguyen LT, Schmidt HA, von Haeseler A, Minh BQ. 2015. IQ-TREE: a fast and effective stochastic algorithm for estimating maximum-likelihood phylogenies. *Mol. Biol. Evol.* 32:268–274.

Petersen TN, Brunak S, von Heijne G, Nielsen H. 2011. SignalP 4.0: discriminating signal peptides from transmembrane regions. *Nat. Methods* 8:785-786.

Richter DJ, Berney C, Strassert JFH, Burki F, de Vargas C. 2021. EukProt: a database of genome-scale predicted proteins across the diversity of eukaryotic life. *bioRxiv* (preprint) doi: https://doi.org/10.1101/2020.06.30.180687 (accessed 16th May 2021)

Simão FA, Waterhouse RM, Ioannidis P, Kriventseva EV, Zdobnov EM. 2015. BUSCO: assessing genome assembly and annotation completeness with single-copy orthologs. *Bioinformatics* 31:3210-3212.

Small I, Peeters N, Legeai F, Lurin C. 2004. Predotar: A tool for rapidly screening proteomes for N-terminal targeting sequences. *Proteomics* 4:1581-1590.

Shen W, Le S, Li Y, Hu F. 2016. SeqKit: A cross-platform and ultrafast toolkit for FASTA/Q manipulation. *PLoS One* 11:e0163962.

Tice AK, et al. 2021. PhyloFisher: A phylogenomic package for resolving eukaryotic relationships. *PLoS Biol*. 19:e3001365.
